# Transition-metal-catalyst-free electroreductive alkene hydroarylation with aryl halides under visible-light irradiation

**DOI:** 10.3762/bjoc.20.116

**Published:** 2024-06-10

**Authors:** Kosuke Yamamoto, Kazuhisa Arita, Masami Kuriyama, Osamu Onomura

**Affiliations:** 1 Graduate School of Biomedical Sciences, Nagasaki University, 1-14 Bunkyo-machi, Nagasaki 852-8521, Japan

**Keywords:** aryl halides, C–C bond formation, electroreduction, radicals, visible light

## Abstract

The radical hydroarylation of alkenes is an efficient strategy for accessing linear alkylarenes with high regioselectivity. Herein, we report the electroreductive hydroarylation of electron-deficient alkenes and styrene derivatives using (hetero)aryl halides under mild reaction conditions. Notably, the present hydroarylation proceeded with high efficiency under transition-metal-catalyst-free conditions. The key to success is the use of 1,3-dicyanobenzene as a redox mediator and visible-light irradiation, which effectively suppresses the formation of simple reduction, i.e., hydrodehalogenation, products to afford the desired products in good to high yields. Mechanistic investigations proposed that a reductive radical-polar crossover pathway is likely to be involved in this transformation.

## Introduction

**Scheme 1:**
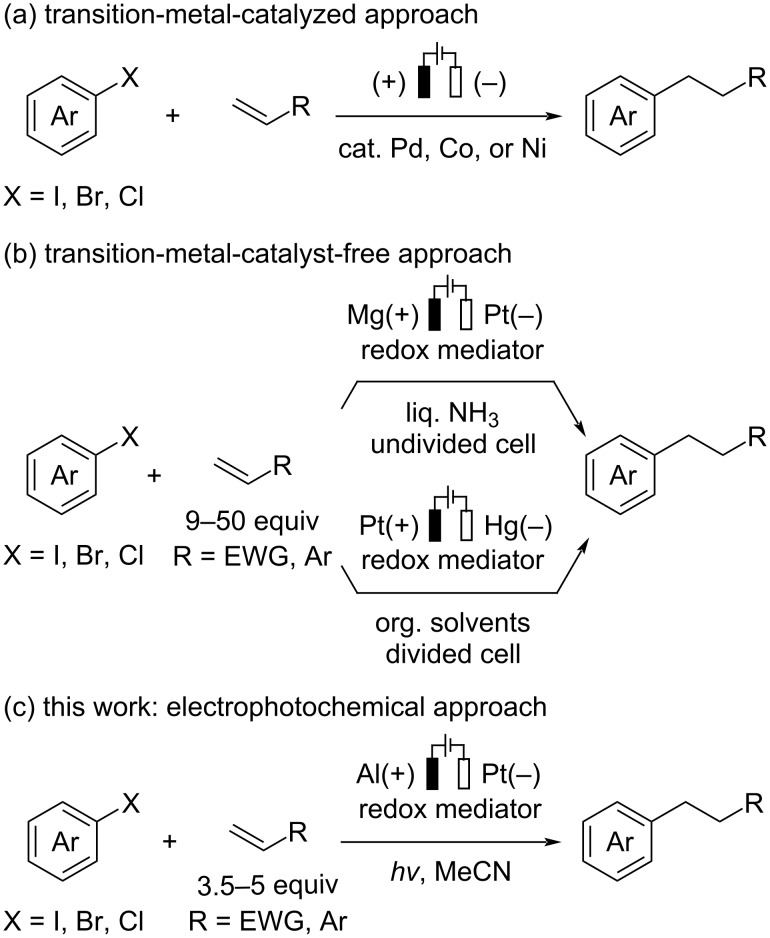
Electrochemical hydroarylation of alkenes with aryl halides.

Alkene hydroarylation is an attractive method for the construction of alkylarenes, which serve as versatile building blocks in organic syntheses. To achieve this transformation with high efficiency and predictable regioselectivity, numerous efforts have been made to develop transition-metal-catalyzed reactions based on a C–H activation strategy [1–4] or the reductive coupling of aryl halides with a hydride donor [5–8]. On the other hand, aryl radical-involved hydroarylation would be a promising alternative for the synthesis of alkylarenes with high anti-Markovnikov selectivity [9–10]. Aryl halides have received increased attention as ideal radical precursors because of their beneficial features, such as higher chemical stability and wide commercial availability, compared with other precursors, e.g., diazonium salts [11]. Classical approaches toward aryl radical species from the corresponding halides would involve halogen abstraction or single-electron reduction processes using chemical reagents; however, these methods have some drawbacks, such as reagent toxicity/stability and limited substrate scope [12–14]. While recent advances in photochemistry have remarkably expanded the synthetic utility of (hetero)aryl radicals in organic synthesis [15–20], visible-light-mediated alkene hydroarylation commonly requires external reductants and/or hydrogen atom sources to complete the catalytic cycle [21–25]. Over the past few decades, electrochemistry has proven to be an environmentally benign and convenient approach for accessing open-shell intermediates through a single-electron transfer process [26–31]. In particular, electroreductive transformations have recently received renewed attention from modern synthetic chemists as a safer protocol than conventional methods using chemical reductants such as metal hydride species [32–36]. In this context, the electrochemical single-electron reduction of aryl iodides, bromides, and activated (bearing at least one electron-withdrawing group) aryl chlorides has been demonstrated as a useful method to generate aryl radical species under mild reaction conditions [37]. Although the additional electron transfer to form the corresponding anions is a highly favorable pathway due to the more positive reduction potential of radicals than that of the starting halides [38], employing redox mediators enables the generated aryl radicals to participate in radical arylation reactions by preventing overreduction [39]. While the metal-catalyst-free radical cyclization of alkene-tethered aryl halides has been well documented in the literature [40–43], the efficient intermolecular hydroarylation of alkenes still relies on the use of transition-metal catalysts, including Pd [44], Ni [45], and Co [46] (Scheme 1a). The pioneering work by Savéant et al. demonstrated that electron-deficient (hetero)aromatics acted as efficient mediators for the metal-catalyst-free electroreductive hydroarylation of alkenes with some activated chloro-, bromo-, and iodoarenes, but the use of a Hg pool cathode and/or liquid NH_3_ solvent would be problematic in terms of environmental and practical perspectives (Scheme 1b) [47]. Therefore, it is desirable to develop an efficient electroreductive protocol for alkene hydroarylation with a broad substrate scope under mild reaction conditions. Recently, the groups of Lin and Lambert [48] and Wickens [49] independently demonstrated that aryl chlorides with highly negative reduction potentials engaged in C–X (X = P, Sn, B) and C–C bond formation reactions involving aryl radical species by integrating photochemistry and electrochemistry [50–53]. Furthermore, odd-numbered [*n*]cumulenes have proven to be effective redox mediators for electroreductive radical borylation of unactivated aryl chlorides without visible-light irradiation by the group of Milner [54]. Herein, we report transition-metal-catalyst-free electroreductive alkene hydroarylation with (hetero)aryl halides using 1,3-dicyanobenzene as a redox mediator under visible-light irradiation.

## Results and Discussion

We began the investigation of the electroreductive hydroarylation using methyl 4-chlorobenzoate (**1a**) and methyl acrylate (**2a**) as model substrates (Table 1). After extensive efforts to screen the reaction parameters to achieve the desired transformation with high efficiency, we found that the electroreductive coupling of **1a** with **2a** proceeded smoothly to afford **3aa** in 82% yield under the conditions using an undivided cell equipped with Al(+)/Pt(−) electrodes in the presence of H_2_O and 5 mol % of 1,3-dicyanobenzene (1,3-DCB) [55] under visible-light irradiation at 0 °C (Table 1, entry 1) [56]. Ammonium salts containing other counter anions also afforded **3aa** in slightly lower yields (Table 1, entries 2 and 3). Changing the sacrificial anode or cathode did not improve the reaction efficiency (Table 1, entries 4–7). The effects of a series of redox mediators on the reaction outcomes were examined, and none gave a better reaction outcome than 1,3-DCB (Table 1, entries 8–12). Control experiments revealed that both visible-light irradiation and the presence of 1,3-DCB were essential for achieving the hydroarylation in high efficiency (Table 1, entries 13–16). Furthermore, the different current density conditions provided the desired product in slightly decreased yields (Table 1, entries 17 and 18), and this transformation did not proceed in the absence of electric current (Table 1, entry 19).

**Table 1:** Evaluation of reaction conditions.^a^

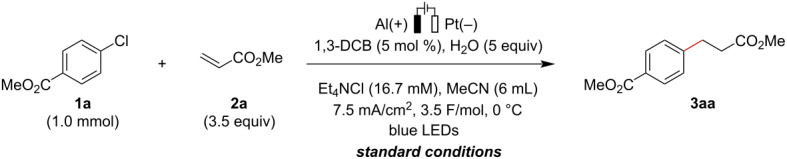		

entry	variation from the standard conditions	yield (%)^b^

1	none	82
2	Et_4_NBr instead of Et_4_NCl	72
3	Et_4_NOTs instead of Et_4_NCl	76
4	Mg anode	50
5	Zn anode	44
6	Ni cathode	55
7	graphite cathode	50
8	1,2-DCB instead of 1,3-DCB	68
9	1,4-DCB instead of 1,3-DCB	75
10	9,10-DCA instead of 1,3-DCB	69
11	phenanthrene (1 equiv) instead of 1,3-DCB, without blue LEDs	60
12	9,9-diethylfluorene (1 equiv) instead of 1,3-DCB, without blue LEDs	65
13	without blue LEDs	55
14	without 1,3-DCB	46
15	without blue LEDs and 1,3-DCB	64
16	without blue LEDs and 1,3-DCB, 2.5 mA/cm^2^	63
17	15 mA/cm^2^	77
18	5 mA/cm^2^	73
19	without electric current	n.r.

^a^Reaction conditions: **1a** (1.0 mmol), **2a** (3.5 mmol), 1,3-DCB (5 mol %), Et_4_NCl (0.1 mmol), H_2_O (5.0 mmol), MeCN (6 mL), Al(+)-Pt(−), 7.5 mA/cm^2^, 3.5 F/mol, 0 °C, blue LEDs. ^b^Isolated yield. DCB, dicyanobenzene; DCA, dicyanoanthracene; n.r., no reaction.

**Scheme 2:**
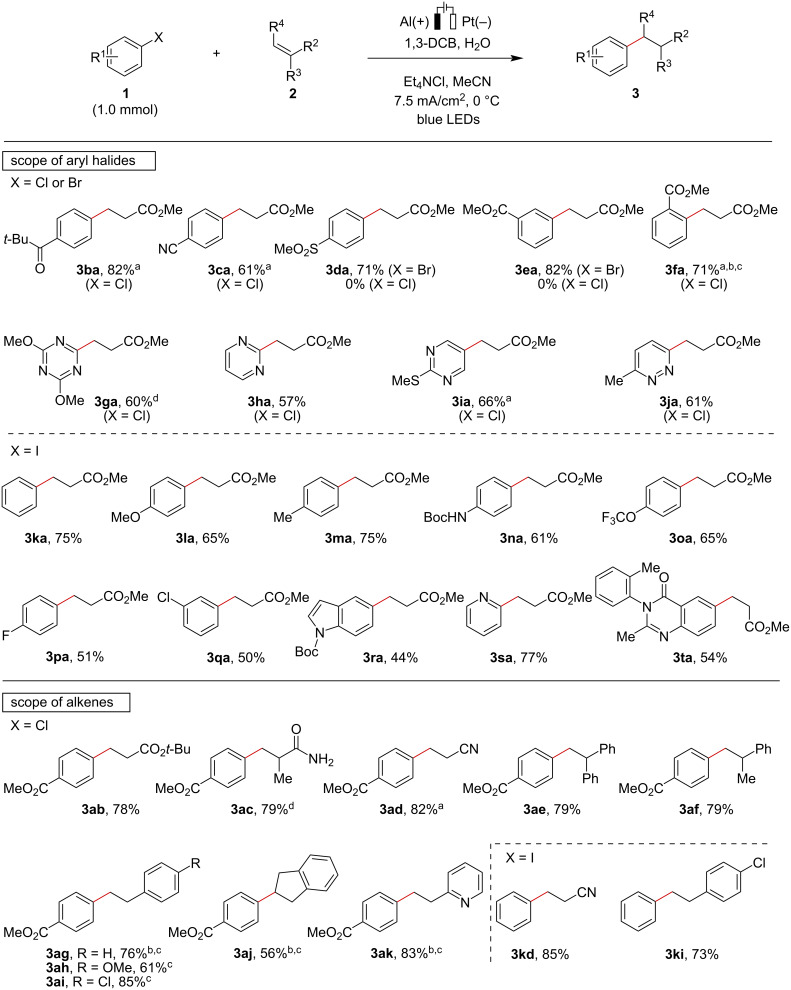
Substrate scope. Reaction conditions for **1** (X = Cl, Br): **1** (1.0 mmol), **2** (3.5 mmol), 1,3-DCB (5 mol %), H_2_O (5.0 mmol), Et_4_NCl (0.1 mmol), MeCN (6 mL), Al(+)-Pt(−), 7.5 mA/cm^2^, 3.5 F/mol, 0 °C, blue LEDs; reaction conditions for **1** (X = I): **1** (1.0 mmol), **2** (5.0 mmol), 1,3-DCB (50 mol %), H_2_O (5.0 mmol), Et_4_NCl (0.1 mmol), MeCN (3 mL), Al(+)-Pt(−), 7.5 mA/cm^2^, 4.5 F/mol, 0 °C, blue LEDs. ^a^4.5 F/mol. ^b^**2** (5 equiv). ^c^MeCN (3 mL). ^d^5 F/mol. 1,3-DCB, 1,3-dicyanobenzene.

With the optimized conditions in hand, the scope of aryl halides and alkenes was investigated (Scheme 2). The reaction of *para*-substituted aryl chlorides bearing pivaloyl and cyano groups proceeded smoothly to provide the desired coupling products in good to high yields (**3ba**, **3ca**). Products bearing *p*-methylsulfonyl (**3da**) and *m*-methoxycarbonyl (**3ea**) groups were obtained from the corresponding aryl bromides instead of chlorides under otherwise identical reaction conditions. The steric hindrance of the *ortho*-substituent did not have a large influence on the reaction efficiency, affording **3fa** in 71% yield. Some heteroaryl chlorides including triazine, pyrimidine, and pyridazine skeletons were also effectively coupled with methyl acrylate to provide the desired products in good yields (**3ga**–**ja**). While unsubstituted chlorobenzene and bromobenzene were completely inert in this transformation, iodobenzene was successfully converted to the corresponding product **3ka** under slightly modified reaction conditions (see Table S1 in Supporting Information File 1 for optimization details). Under the modified conditions, aryl iodides with various electron-donating groups including a methoxy group were transformed into the products in good yields (**3la**–**na**). 4-(Trifluoromethoxy)iodobenzene also well participated in this reaction, affording **3oa** in 65% yield. Aryl iodides having fluoro and chloro substituents underwent selective C–I bond cleavage to provide monoalkylated products **3pa** and **3qa**, respectively. In addition to the successful transformations of heteroaryl iodides with indole or pyridine cores (**3ra**, **3sa**), the electroreductive synthesis of methaqualone derivatives was also achieved (**3ta**).

Pleasingly, a series of electron-deficient alkene and styrene derivatives were found to be suitable coupling partners. Aryl chloride **1a** reacted with *tert*-butyl acrylate (**2b**) without any difficulty, providing **3ab** in a high yield. Methacrylamide (**2c**) and acrylonitrile (**2d**) were transformed into the corresponding products in high yields, but with slightly lower Faradaic efficiency (**3ac**, **3ad**). In addition to styrene derivatives bearing α-substituents and electronically diverse functionalities, indene and 2-vinylpyridine were all compatible with the present electroreductive hydroarylation (**3ae**–**ak**). The reaction of iodobenzene with acrylonitrile and 4-chlorostyrene proceeded smoothly to afford **3kd** and **3ki** in 85% and 73% yields, respectively.

**Scheme 3:**
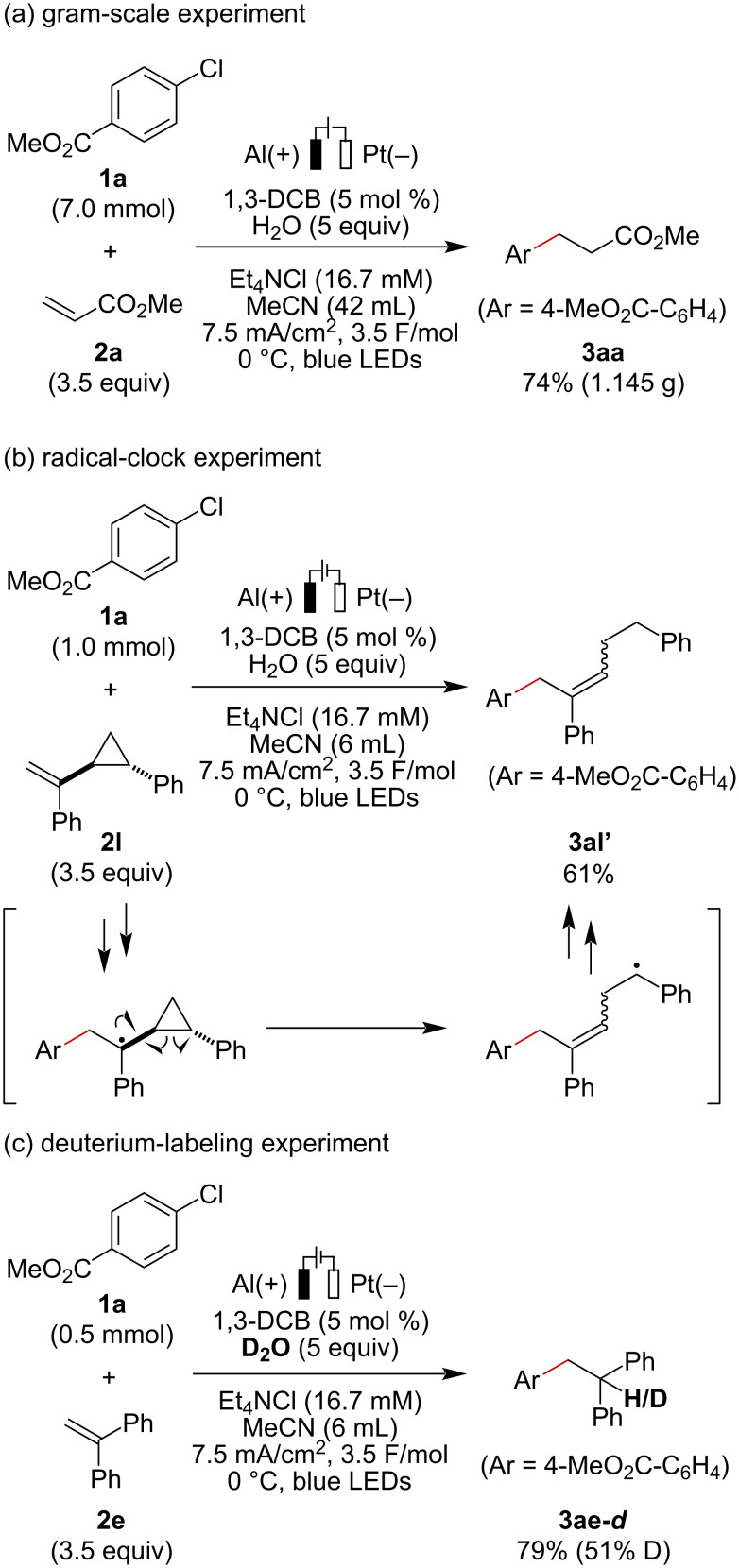
Gram-scale reaction and control experiments.

In order to demonstrate the scalability of this transformation, a gram-scale reaction was performed (Scheme 3a). The hydroarylation of **2a** with **1a** was successfully carried out in a simple glass beaker under the standard reaction conditions, providing the corresponding product **3aa** in 74% yield. Several control experiments were conducted to gain insight into the reaction mechanism of the electroreductive process. The hydroarylation of cyclopropane-substituted styrene **2l** resulted in the formation of ring-opening product **3al’**, and the simple hydroarylation product was not observed (Scheme 3b). This result strongly supported the involvement of radical intermediates in the present transformation. Next, a deuterium-labeling experiment was conducted to elucidate the H-source of this reaction (Scheme 3c). The reaction of **1a** and **2e** in MeCN with D_2_O provided the coupling product with 51% deuterium incorporation, indicating that carbanion species would be generated in this reaction and that H_2_O would serve as a major proton source. Et_4_NCl may also provide protons to form the coupling product [47]. Taken these results together, the present electroreductive reaction would proceed through a reductive radical-polar crossover pathway [57].

**Scheme 4:**
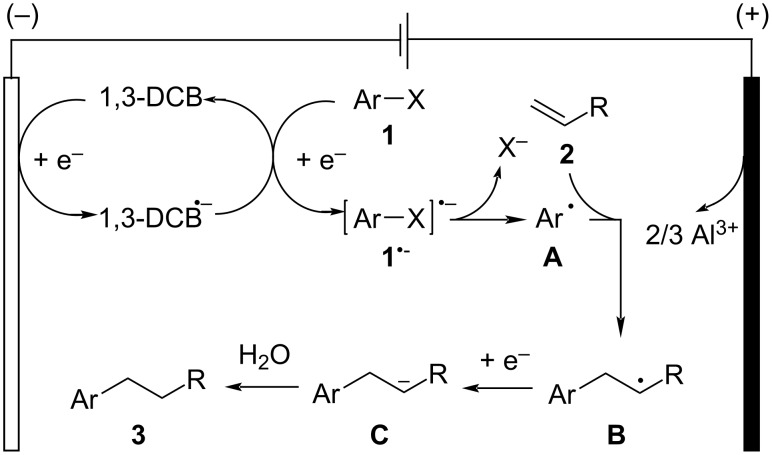
Plausible mechanism.

On the basis of mechanistic investigations and a literature report [47], a plausible mechanism for this electroreductive hydroarylation is depicted in Scheme 4. 1,3-DCB (*E*_p/2_ = −1.9 V vs SCE in MeCN) [58] undergoes single-electron reduction at the cathode to generate the radical anion species (1,3-DCB^•−^), which might act as a mediator to produce the radical anion **1**^•−^ through the homogeneous electron transfer in the bulk solution (representative reduction peak potential: **1a**, *E*_p_ = −1.98 V vs SCE in MeCN [59]; **1c**, *E*_p_ = −2.06 V vs SCE in MeCN [47]). In the case of the reaction with electron-deficient alkenes, e.g., methyl acrylate (*E*_1/2_ = −2.1 V vs SCE) [60], reduction of alkenes is one of the competitive processes. Subsequent fragmentation of radical anion **1**^•−^ to form aryl radical species **A**, which then reacts with alkene **2** to provide alkyl radical species **B**. Further single-electron reduction by 1,3-DCB^•−^ or at the cathode followed by protonation of **B** provides hydroarylation product **3**. Meanwhile, the sacrificial anode is oxidized to form Al cations. Although the exact role of visible-light irradiation in the electroreductive hydroarylation is unclear, the generation of photoexcited radical anion species as potential reductants might be included in the present transformation.

## Conclusion

In conclusion, we have developed a transition-metal-catalyst-free electroreductive hydroarylation of alkenes with aryl halides, including aryl chlorides, by employing 1,3-DCB under visible-light irradiation. The present transformation proceeded smoothly in a common organic solvent without transition-metal catalysts, and hydroarylation products were obtained from a variety of electron-deficient alkenes and styrene derivatives in good to high yields. A large-scale reaction was successfully carried out, highlighting the potential synthetic utility of the present transformation. The mechanistic study proposed that a reductive radical-polar crossover pathway would be involved in the present transformation.

## Supporting Information

File 1 Experimental procedures, characterization data, and copies of NMR spectra of the products.

## Data Availability

All data that supports the findings of this study is available in the published article and/or the supporting information to this article.
