# Supplementary material for: Transition-metal-catalyst-free electroreductive alkene hydroarylation with aryl halides under visible-light irradiation
Source: Beilstein J Org Chem. 2024 Jun 10;20:1327–33. doi: 10.3762/bjoc.20.116 (PMC11181238; doi:10.3762/bjoc.20.116)
Supplement: File 1 — Experimental procedures, characterization data, and copies of NMR spectra of the products. [file Beilstein_J_Org_Chem-20-1327-s001.pdf]

BEILSTEIN JOURNAL OF ORGANIC CHEMISTRY

Supporting Information

for

Transition-metal-catalyst-free electroreductive alkene hydroarylation with aryl halides under visible-light irradiation

Kosuke Yamamoto, Kazuhisa Arita, Masami Kuriyama and Osamu Onomura

Beilstein J. Org. Chem. **2024,** *20,* 1327–1333. doi:10.3762/bjoc.20.116

Experimental procedures, characterization data, and copies of NMR spectra of the products

License and Terms: This is a supporting information file under the terms of the Creative Commons Attribution License (https://creativecommons.org/licenses/by/4.0). Please note that the reuse, redistribution and reproduction in particular requires that the author(s) and source are credited and that individual graphics may be subject to special legal provisions.
The license is subject to the *Beilstein Journal of Organic Chemistry* terms and conditions: (https://www.beilstein-journals.org/bjoc/terms)

Table of Contents

1. General information S1

2. Experimental procedures and characterization data

   2.1. Optimization of reaction conditions for aryl iodide **1k** S1

   2.2. General procedure for the electroreductive hydroarylation of alkenes S2-S12

   2.3. Gram-scale experiment S13-S14

   2.4. Radical-clock experiment S14

   2.5. Deuterium-labeling experiment S15

3. References S16

4. ^{1}H, $^{13}C\{^{1}H\}$, and ^{19}F NMR spectra S17-S52

1. General information

Unless otherwise noted, all reactions were performed under air atmosphere. Electrochemical reactions were carried out using a DC power supply (GP 050-2, *Takasago Ltd.*) with a Coulomb/Amperehour meter (HF-201A, *Hokuto Denko Corporation*). A *Kessil* A160WE Tuna Blue LED lamp (40W) was used for light irradiation. The products were isolated by flash column chromatography (CHROMATOREX 60B, *Fuji silysia*). Melting points (mp) were measured with a *YANAKO* MP-J3 Micro Melting Point Apparatus and reported without correction. Infrared (IR) spectra were recorded on a *SHIMADZU* IRAffinity-1 spectrometer and expressed as frequency of absorption (cm^{-1}). ^1H, $^{13}C\{^1H\}$, and ^{19}F NMR spectra were recorded on a *JEOL* JNM-ECZ400R (400 MHz for ^1H NMR, 100 MHz for $^{13}C\{^1H\}$ NMR, and 376 MHz for ^{19}F NMR). Chemical shift values are expressed in parts per million (ppm) relative to internal TMS (δ 0.00 ppm for ^1H NMR) and $CDCl_3$ (δ 77.0 ppm for $^{13}C\{^1H\}$ NMR). Abbreviations are as follows: s, singlet; d, doublet; t, triplet; q, quartet; m, multiplet; br, broad. High-resolution mass spectra (HRMS) were recorded on a *JEOL* JMS-T100TD spectrometer using the electrospray ionization (ESI) technique.

Commercially available chemicals were purchased from Sigma-Aldrich, Tokyo Chemical Industry Co., Ltd., Nacalai Tesque, Inc., and FUJIFILM Wako Pure Chemical Corporation and used as received unless otherwise noted. Anhydrous acetonitrile was purchased from FUJIFILM Wako Pure Chemical Corporation (*super dehydrated* grade) and used as received. *Super dehydrated* grade of acetonitrile was dried over 4Å molecular sieves prior to use for a deuterium-labeling experiment.

2. Experimental procedures and characterization data

2.1. Optimization of reaction conditions for aryl iodide 1k

Table S1. Optimization of the reaction conditions

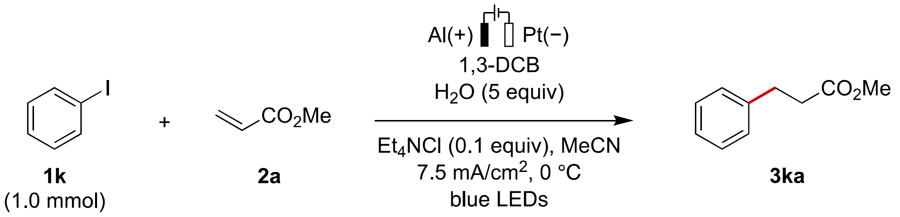

entry	1,3-DCB (mol %)	**2a** (equiv)	MeCN (mL)	charge (F/mol)	yield (%)[a]
1	5	3.5	6	3.5	25
2	50	3.5	6	3.5	61 (8)[b]
3	100	3.5	6	3.5	63
4	50	5	6	3.5	61 (16)[b]
5	50	5	6	4.5	75
6	50	5	3	4.5	75
7[c]	50	5	3	4.5	42

[a]Isolated yield. [b]NMR yield of unreacted **1k** in parentheses.
[c]No LEDs.

2.2. General procedure for the electroreductive hydroarylation of alkenes

General procedure A (for aryl chlorides and bromides)

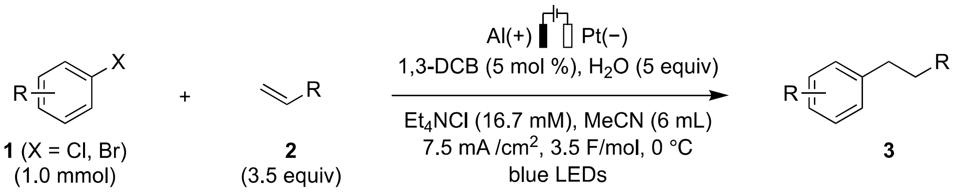

A cylinder-type undivided cell with a stir bar was charged with Et_4NCl (16.6 mg, 0.1 mmol), 1,3-dicyanobenzene (1,3-DCB, 6.4 mg, 0.05 mmol), alkene **2** (3.5 mmol), and aryl halide **1** (1.0 mmol). Anhydrous MeCN (6.0 mL) and H_2O (90 μL) were successively added. The reaction vessel was equipped with a Pt plate cathode (immersed surface area: 1 × 2 cm^2) and an Al plate anode (immersed surface area: 1 × 2 cm^2) under air, then 3.5 F/mol of electricity was passed through at a constant current condition (7.5 mA/cm^2) under visible-light irradiation (approximate distance from the cathode: 2.5 cm) at 0 °C in an ice bath (Figure S1). Most of solvent was removed under reduced pressure, and the residue was diluted with AcOEt. The organic layer was washed with H_2O, dried over Na_2SO_4, filtered, and concentrated under reduced pressure. The residue was purified by flash silica gel column chromatography to afford the desired product **3**.

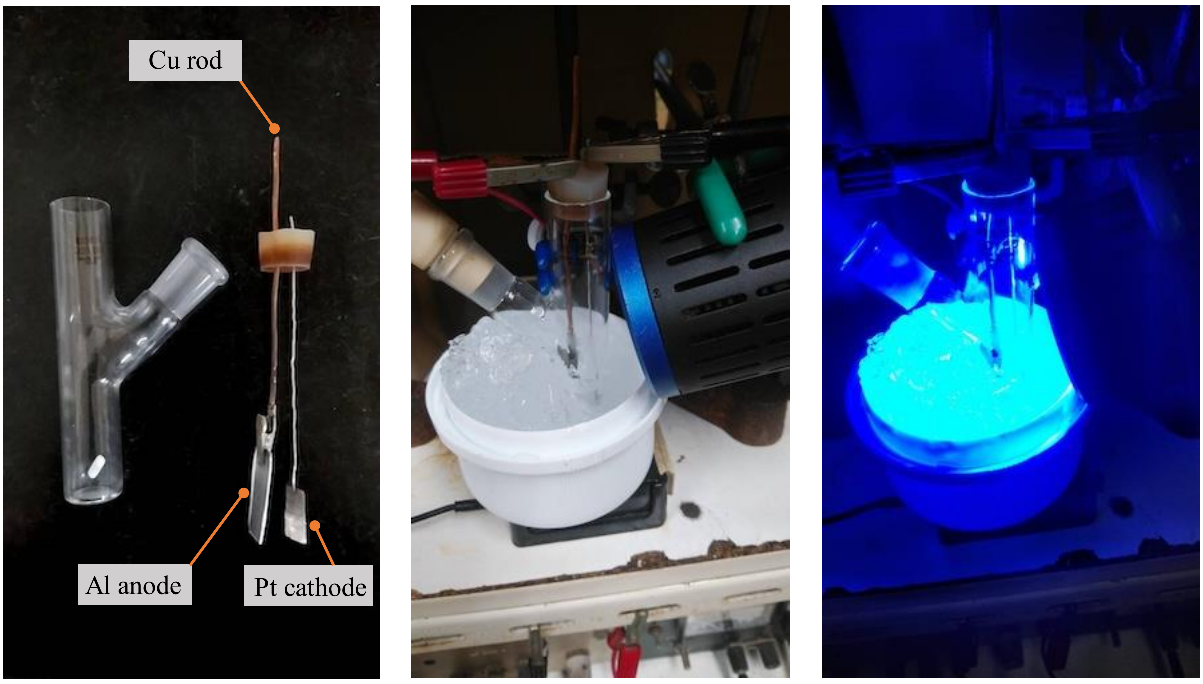

Figure S1. A reaction vessel and a silicone stopper equipped with Al and Pt electrodes (left) and a typical reaction setup for the electroreductive hydroarylation under visible-right irradiation (middle and right).

General procedure B (for aryl iodides)

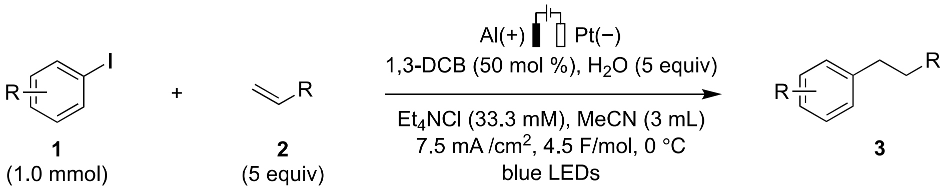

A cylinder-type undivided cell with a stir bar was charged with Et_4NCl (16.6 mg, 0.1 mmol), 1,3-dicyanobenzene (1,3-DCB, 64.1 mg, 0.5 mmol), alkene **2** (5.0 mmol), and aryl iodide **1** (1.0 mmol). Anhydrous MeCN (3.0 mL) and H_2O (90 μL) were successively added. The reaction vessel was equipped with a Pt plate cathode (immersed surface area: 1 × 2 cm^2) and an Al plate anode (immersed surface area: 1 × 2 cm^2) under air, then 4.5 F/mol of electricity was passed through at a constant current condition (7.5 mA/cm^2) under visible-light irradiation (approximate distance from the cathode: 2.5 cm) at 0 °C in an ice bath. Most of solvent was removed under reduced pressure, and the residue was diluted with AcOEt. The organic layer was washed with H_2O, dried over Na_2SO_4, filtered, and concentrated under reduced pressure. The residue was purified by flash silica gel column chromatography to afford the desired product **3**.

Methyl 4-(3-methoxy-3-oxopropyl)benzoate (3aa)

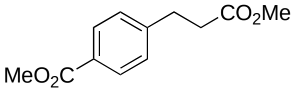

The title compound was obtained from methyl 4-chlorobenzoate (171 mg, 1.0 mmol) and methyl acrylate (301 mg, 3.5 mmol). Silica gel column chromatography (hexane/AcOEt = 8:1) gave **3aa** (183.3 mg, 0.825 mmol, 82%) as a colorless oil. ^{1}H NMR (400 MHz, $CDCl_3$): δ 7.98-7.95 (m, 2H), 7.28-7.26 (m, 2H), 3.90 (s, 3H), 3.67 (s, 3H), 3.01 (t, J = 7.7 Hz, 2H), 2.66 (t, J = 7.8 Hz, 2H); ^{13}C{^{1}H} NMR (100 MHz, $CDCl_3$): δ 172.8, 166.8, 145.8, 129.7, 128.2, 128.1, 51.8, 51.5, 35.0, 30.7.
The ^{1}H and ^{13}C{^{1}H} NMR spectra are in accordance with those reported in the literature.[1]

Methyl 3-(4-pivaloylphenyl)propanoate (3ba)

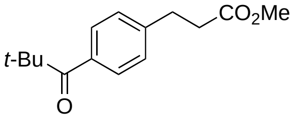

The title compound was obtained from 1-(4-chlorophenyl)-2,2-dimethyl-1-propanone (197 mg, 1.0 mmol) and methyl acrylate (301 mg, 3.5 mmol) with 4.5 F/mol of electricity. Silica gel column chromatography (hexane/AcOEt = 20:1) gave **3ba** (202.7 mg, 0.816 mmol, 82%) as a colorless oil. ^{1}H NMR (400 MHz, $CDCl_3$): δ 7.69-7.66 (m, 2H), 7.23 (d, J = 8.2 Hz, 2H), 3.68 (s, 3H), 2.99 (t, J = 7.8 Hz, 2H), 2.65 (t, J = 7.8 Hz, 2H), 1.35 (s, 9H); ^{13}C{^{1}H} NMR (100 MHz, $CDCl_3$): δ 208.1, 172.8, 143.7, 136.1, 128.3, 127.8, 51.5, 43.9, 35.0, 30.5, 27.9; IR (ATR): 2953, 1736, 1670, 1607, 1275, 1171, 959 cm^{-1}; HRMS (ESI) *m/z*: [M+Na]$^+$ calcd for $C_{15}H_{20}NaO_3$ 271.1310, found 271.1316.

Methyl 3-(4-cyanophenyl)propanoate (3ca)

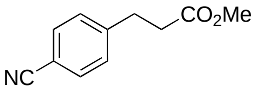

The title compound was obtained from 4-chlorobenzonitrile (138 mg, 1.0 mmol) and methyl acrylate (301 mg, 3.5 mmol) with 4.5 F/mol of electricity. Silica gel column chromatography (hexane/AcOEt = 4:1) gave **3ca** (114.6 mg, 0.606 mmol, 61%) as a white solid. ^{1}H NMR (400 MHz, $CDCl_3$): δ 7.60-7.58 (m, 2H), 7.33-7.31 (m, 2H), 3.67 (s, 3H), 3.01 (t, J = 7.6 Hz, 2H), 2.66 (t, J = 7.6 Hz, 2H); $^{13}C\{^1H\}$ NMR (100 MHz, $CDCl_3$): δ 172.6, 146.0, 132.3, 129.1, 118.8, 110.2, 51.7, 34.7, 30.8.
The ^{1}H and $^{13}C\{^1H\}$ NMR spectra are in accordance with those reported in the literature.[2]

Methyl 3-(4-(methylsulfonyl)phenyl)propanoate (3da)

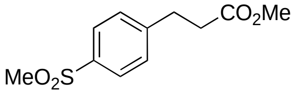

The title compound was obtained from 4-bromophenyl methyl sulfone (235 mg, 1.0 mmol) and methyl acrylate (301 mg, 3.5 mmol). Silica gel column chromatography (hexane/AcOEt = 2:1) gave **3da** (170.8 mg, 0.705 mmol, 71%) as a white solid. mp 67–68 °C; ^{1}H NMR (400 MHz, $CDCl_3$): δ 7.89-7.85 (m, 2H), 7.42-7.40 (m, 2H), 3.68 (s, 3H), 3.051 (t, J = 7.7 Hz, 2H), 3.046 (s, 3H), 2.68 (t, J = 7.7 Hz, 2H); $^{13}C\{^1H\}$ NMR (100 MHz, $CDCl_3$): δ 172.4, 146.8, 138.3, 129.1, 127.3, 51.5, 44.2, 34.6, 30.3; IR (ATR): 2911, 1730, 1597, 1290, 1141, 1087 cm^{-1}; HRMS (ESI) *m/z*: $[M+Na]^+$ calcd for $C_{11}H_{14}NaO_4S$ 265.0511, found 265.0513.
The ^{1}H and $^{13}C\{^1H\}$ NMR spectra are in accordance with those reported in the literature.[3]

Methyl 3-(3-methoxy-3-oxopropyl)benzoate (3ea)

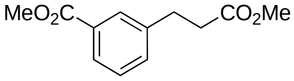

The title compound was obtained from methyl 3-bromobenzoate (215 mg, 1.0 mmol) and methyl acrylate (301 mg, 3.5 mmol). Silica gel column chromatography (hexane/AcOEt = 20:1) gave **3ea** (182.3 mg, 0.820 mmol, 82%) as a yellow oil. ^{1}H NMR (400 MHz, $CDCl_3$): δ 7.90-7.87 (m, 2H), 7.42-7.34 (m, 2H), 3.91 (s, 3H), 3.67 (s, 3H), 3.00 (t, J = 7.8 Hz, 2H), 2.66 (t, J = 7.8 Hz, 2H); $^{13}C\{^1H\}$ NMR (100 MHz, $CDCl_3$): δ 172.9, 166.9, 140.7, 132.9, 130.2, 129.2, 128.4, 127.5, 52.0, 51.5, 35.3, 30.5; IR (ATR): 2953, 1719, 1435, 1281, 1198 cm^{-1}; HRMS (ESI) *m/z*: $[M+Na]^+$ calcd for $C_{12}H_{14}NaO_4$ 245.0790, found 245.0778.

Methyl 2-(3-methoxy-3-oxopropyl)benzoate (3fa)

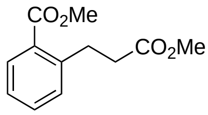

The title compound was obtained from methyl 2-chlorobenzoate (171 mg, 1.0 mmol) and methyl

acrylate (431 mg, 5.0 mmol) with 4.5 F/mol of electricity using MeCN (3.0 mL). Silica gel column chromatography (hexane/AcOEt/CH_2Cl_2 = 12:2:1) gave **3fa** (156.9 mg, 0.706 mmol, 71%) as a colorless oil. ^{1}H NMR (400 MHz, $CDCl_3$): δ 7.93-7.91 (m, 1H), 7.46-7.42 (m, 1H), 7.30-7.26 (m, 2H), 3.90 (s, 3H), 3.67 (s, 3H), 3.28 (t, J = 7.8 Hz, 2H), 2.68 (t, J = 7.8 Hz, 2H); $^{13}C\{^1H\}$ NMR (100 MHz, $CDCl_3$): δ 173.2, 167.4, 142.3, 132.1, 131.0, 130.7, 129.1, 126.3, 51.8, 51.3, 35.4, 29.7.
The ^{1}H and $^{13}C\{^1H\}$ NMR spectra are in accordance with those reported in the literature.[4]

Methyl 3-(4,6-dimethoxy-1,3,5-triazin-2-yl)propanoate (3ga)

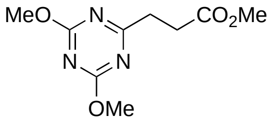

The title compound was obtained from 2-chloro-4,6-dimethoxy-1,3,5-triazine (176 mg, 1.0 mmol) and methyl acrylate (301 mg, 3.5 mmol) with 5 F/mol of electricity. Silica gel column chromatography (hexane/AcOEt = 3:1) gave **3ga** (135.6mg, 0.597 mmol, 60%) as a colorless oil. ^{1}H NMR (400 MHz, $CDCl_3$): δ 4.02 (s, 6H), 3.69 (s, 3H), 3.10 (t, J = 7.1 Hz, 2H), 2.85 (t, J = 7.0 Hz, 2H); $^{13}C\{^1H\}$ NMR (100 MHz, $CDCl_3$): δ 181.2, 172.9, 172.1, 54.9, 51.5, 32.8, 30.0; IR (ATR): 2959, 1728, 1543, 1340, 1277, 1171, 1105 cm^{-1}; HRMS (ESI) *m/z*: [M+Na]$^+$ calcd for $C_9H_{13}N_3NaO_4$ 250.0804, found 250.0797.

Methyl 3-(pyrimidin-2-yl)propanoate (3ha)

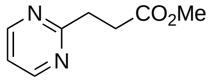

The title compound was obtained from 2-chloropyrimidine (115 mg, 1.0 mmol) and methyl acrylate (301 mg, 3.5 mmol). Silica gel column chromatography (hexane/AcOEt = 4:1) gave **3ha** (94.6 mg, 0.569 mmol, 57%) as a colorless oil. ^{1}H NMR (400 MHz, $CDCl_3$): δ 8.66 (d, J = 5.0 Hz, 2H), 7.14 (t, J = 4.9 Hz, 1H), 3.69 (s, 3H), 3.32 (t, J = 7.2 Hz, 2H), 2.90 (t, J = 7.2 Hz, 2H); $^{13}C\{^1H\}$ NMR (100 MHz, $CDCl_3$): δ 173.3, 169.2, 156.8, 118.6, 51.5, 33.6, 31.3; IR (ATR): 2953, 1730, 1562, 1427, 1368, 1167 cm^{-1}; HRMS (ESI) *m/z*: [M+Na]$^+$ calcd for $C_8H_{10}N_2NaO_2$ 189.0640, found 189.0643.

Methyl 3-(2-(methylthio)pyrimidin-5-yl)propanoate (3ia)

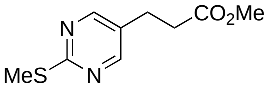

The title compound was obtained from 5-chloro-2-(methylthio)pyrimidine (161 mg, 1.0 mmol) and methyl acrylate (301 mg, 3.5 mmol) with 4.5 F/mol of electricity. Silica gel column chromatography (hexane/AcOEt = 4:1) gave **3ia** (140.0 mg, 0.660 mmol, 66%) as a yellow solid. mp 37–38 °C; ^{1}H NMR (400 MHz, $CDCl_3$): δ 8.41 (s, 2H), 3.68 (s, 3H), 2.87 (t, J = 7.3 Hz, 2H), 2.63 (t, J = 7.3 Hz, 2H), 2.56 (s, 3H); $^{13}C\{^1H\}$ NMR (100 MHz, $CDCl_3$): δ 172.1, 170.3, 156.9, 128.0, 51.6, 34.5, 24.7, 13.8; IR (ATR): 2918, 1728, 1581, 1537, 1389, 1206, 1167 cm^{-1}; HRMS (ESI) *m/z*: [M+Na]$^+$ calcd for $C_9H_{12}N_2NaO_2S$ 235,0517, found 235.0515.

Methyl 3-(6-methylpyridazin-3-yl)propanoate (3ja)

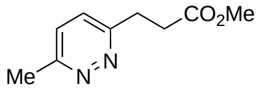

The title compound was obtained from 3-chloro-6-methylpyridazine (129 mg, 1.0 mmol) and methyl acrylate (301 mg, 3.5 mmol). Silica gel column chromatography (AcOEt 100%) gave **3ja** (110.6 mg, 0.614 mmol, 61%) as a colorless viscous oil which solidified in a freezer. ^{1}H NMR (400 MHz, $CDCl_3$): δ 7.28 (d, J = 8.7 Hz, 1H), 7.23 (d, J = 8.5 Hz, 1H), 3.67 (s, 3H), 3.23 (t, J = 7.2 Hz, 2H), 2.93 (t, J = 7.2 Hz, 2H), 2.68 (s, 3H); ^{13}C{^{1}H} NMR (100 MHz, $CDCl_3$): δ 173.1, 159.1, 158.0, 126.8, 126.6, 51.5, 32.4, 30.4, 21.8; IR (ATR): 1953, 1728, 1443, 1361, 1206, 1161 cm^{-1}; HRMS (ESI) *m/z*: $[M+Na]^+$ calcd for $C_9H_{12}N_2NaO_2$ 203.0797, found 203.0791.

Methyl 3-phenylpropanoate (3ka)

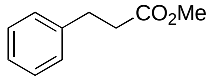

The title compound was obtained from iodobenzene (204 mg, 1.0 mmol) and methyl acrylate (431 mg, 5.0 mmol). Silica gel column chromatography (hexane/AcOEt = 20:1) gave **3ka** (123.4mg, 0.752 mmol, 75%) as a colorless oil. ^{1}H NMR (400 MHz, $CDCl_3$): δ 7.31-7.27 (m, 2H), 7.22-7.19 (m, 3H), 3.67 (s, 3H), 2.96 (t, J = 7.9 Hz, 2H), 2.66-2.62 (m, 2H); ^{13}C{^{1}H} NMR (100 MHz, $CDCl_3$): δ 173.2, 140.4, 128.4, 128.2, 126.2, 51.5, 35.6, 30.8.
The ^{1}H and ^{13}C{^{1}H} NMR spectra are in accordance with those reported in the literature.[2]

Methyl 3-(4-methoxyphenyl)propanoate (3la)

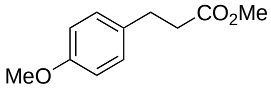

The title compound was obtained from 4-iodoanisole (234 mg, 1.0 mmol) and methyl acrylate (431 mg, 5.0 mmol). Silica gel column chromatography (hexane/AcOEt = 20:1) gave **3la** (126.8 mg, 0.653 mmol, 65%) as a white solid. ^{1}H NMR (400 MHz, $CDCl_3$): δ 7.14-7.10 (m, 2H), 6.85-6.82 (m, 2H), 3.79 (s, 3H), 3.67 (s, 3H), 2.89 (t, J = 7.9 Hz, 2H), 2.60 (t, J = 7.8 Hz, 2H); ^{13}C{^{1}H} NMR (100 MHz, $CDCl_3$): δ 173.3, 157.9, 132.4, 129.1, 113.7, 55.0, 51.4, 35.9, 29.9.
The ^{1}H and ^{13}C{^{1}H} NMR spectra are in accordance with those reported in the literature.[2]

Methyl 3-(p-tolyl)propanoate (3ma)

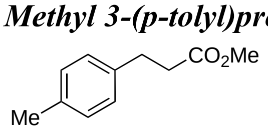

The title compound was obtained from 4-iodotoluene (218 mg, 1.0 mmol) and methyl acrylate (431 mg, 5.0 mmol). Silica gel column chromatography (hexane/AcOEt = 20:1) gave **3ma** (134.0 mg, 0.752 mmol, 75%) as a white solid. ^{1}H NMR (400 MHz, $CDCl_3$): δ 7.12-7.07 (m, 4H), 3.67 (s, 3H), 2.91 (t, J = 7.8 Hz, 2H), 2.61 (t, J = 7.8 Hz, 2H), 2.32 (s, 3H); ^{13}C{^{1}H} NMR (100 MHz, $CDCl_3$): δ 173.4,

137.4, 135.7, 129.1, 128.1, 51.6, 35.8, 30.5, 21.0.
The ^{1}H and ^{13}C{^{1}H} NMR spectra are in accordance with those reported in the literature.[2]

Methyl 3-(4-(tert-butoxycarbonylamino)phenyl)propanoate (3na)

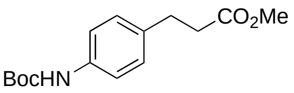

The title compound was obtained from *N*-Boc-4-iodoaniline (319 mg, 1.0 mmol) and methyl acrylate (431 mg, 5.0 mmol). Silica gel column chromatography (hexane/AcOEt = 8:1) gave **3na** (170.4 mg, 0.610 mmol, 61%) as a white solid. mp 65–66 °C; ^{1}H NMR (400 MHz, $CDCl_3$): δ 7.28-7.26 (m, 2H), 7.12 (d, J = 8.5 Hz, 2H), 6.41 (br s, 1H), 3.66 (s, 3H), 2.90 (t, J = 7.8 Hz, 2H), 2.59 (t, J = 7.8 Hz, 2H), 1.51 (s, 9H); ^{13}C{^{1}H} NMR (100 MHz, $CDCl_3$): δ 173.3, 152.8, 136.5, 135.1, 128.7, 118.7, 80.4, 51.6, 35.8, 30.2, 28.3; IR (ATR): 3343, 2980, 1715, 1699, 1525, 1234, 1158 cm^{-1}; HRMS (ESI) *m/z*: $[M+Na]^+$ calcd for $C_{15}H_{21}NNaO_4$ 302.1368, found 302.1362.
The ^{1}H NMR spectrum is in accordance with those reported in the literature.[5]

Methyl 3-(4-(trifluoromethoxy)phenyl)propanoate (3oa)

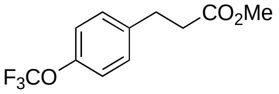

The title compound was obtained from 1-iodo-4-(trifluoromethoxy)benzene (288 mg, 1.0 mmol) and methyl acrylate (431 mg, 5.0 mmol). Silica gel column chromatography (hexane/AcOEt = 20:1) gave **3oa** (161.1 mg, 0.649 mmol, 65%) as a colorless oil. ^{1}H NMR (400 MHz, $CDCl_3$): δ 7.24-7.20 (m, 2H), 7.15-7.12 (m, 2H), 3.67 (s, 3H), 2.96 (t, J = 7.8 Hz, 2H), 2.63 (t, J = 7.7 Hz, 2H); ^{13}C{^{1}H} NMR (100 MHz, $CDCl_3$): δ 173.0, 147.7 (q, J = 1.9 Hz), 139.2, 129.6, 121.0, 120.4 (q, J = 256.6 Hz), 51.6, 35.4, 30.1; ^{19}F NMR (376 MHz, $CDCl_3$): δ −57.8.
The ^{1}H and ^{13}C{^{1}H} NMR spectra are in accordance with those reported in the literature.[6]

Methyl 3-(4-fluorophenyl)propanoate (3pa)

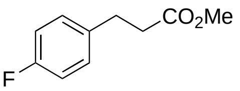

The title compound was obtained from 4-fluoroiodobenzene (222 mg, 1.0 mmol) and methyl acrylate (431 mg, 5.0 mmol). Silica gel column chromatography (hexane/AcOEt = 20:1) gave **3pa** (93.1 mg, 0.511 mmol, 51%) as a colorless oil. ^{1}H NMR (400 MHz, $CDCl_3$): δ 7.18-7.13 (m, 2H), 7.00-6.94 (m, 2H), 3.67 (s, 3H), 2.92 (t, J = 7.7 Hz, 2H), 2.61 (t, J = 7.7 Hz, 2H); ^{13}C{^{1}H} NMR (100 MHz, $CDCl_3$): δ 173.1, 161.4 (d, J = 243.7 Hz), 136.0 (d, J = 3.9 Hz), 129.6 (d, J = 7.7 Hz), 115.1 (d, J = 21.2 Hz), 51.5, 35.7, 30.0; ^{19}F NMR (376 MHz, $CDCl_3$): δ −116.9.
The ^{1}H and ^{13}C{^{1}H} NMR spectra are in accordance with those reported in the literature.[2]

Methyl 3-(3-chlorophenyl)propanoate (3qa)

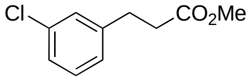

The title compound was obtained from 3-chloroiodobenzene (239 mg, 1.0 mmol) and methyl acrylate (431 mg, 5.0 mmol). Silica gel column chromatography (hexane/AcOEt = 20:1) gave **3qa** (100.0 mg, 0.503 mmol, 50%) as a yellow oil. ^{1}H NMR (400 MHz, $CDCl_3$): δ 7.24-7.17 (m, 3H), 7.10-7.07 (m, 1H), 3.68 (s, 3H), 2.93 (t, J = 7.8 Hz, 2H), 2.63 (t, J = 7.8 Hz, 2H); ^{13}C{^{1}H} NMR (100 MHz, $CDCl_3$): δ 173.0, 142.5, 134.2, 129.7, 128.4, 126.5, 51.7, 35.3, 30.5.
The ^{1}H and ^{13}C{^{1}H} NMR spectra are in accordance with those reported in the literature.[7]

tert-Butyl 5-(3-methoxy-3-oxopropyl)-1H-indole-1-carboxylate (3ra)

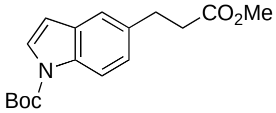

The title compound was obtained from *N*-Boc-5-iodoindole (343 mg, 1.0 mmol) and methyl acrylate (431 mg, 5.0 mmol). Silica gel column chromatography (hexane/AcOEt = 20:1) gave **3ra** (134.0 mg, 0.442 mmol, 44%) as a colorless viscous oil which solidified in a freezer. ^{1}H NMR (400 MHz, $CDCl_3$): δ 8.04 (d, J = 8.2 Hz, 1H), 7.57 (d, J = 3.4 Hz, 1H), 7.38 (d, J = 1.1 Hz, 1H), 7.15 (dd, J = 8.6, 1.7 Hz, 1H), 6.51 (d, J = 3.7 Hz, 1H), 3.66 (s, 3H), 3.04 (t, J = 7.8 Hz, 2H), 2.67 (t, J = 7.9 Hz, 2H), 1.66 (s, 9H); ^{13}C{^{1}H} NMR (100 MHz, $CDCl_3$): δ 173.4, 149.7, 134.8, 133.8, 130.8, 126.1, 124.6, 120.2, 115.0, 107.0, 83.5, 51.5, 36.2, 30.8, 28.1; IR (ATR): 2974, 1728, 1468, 1350, 1157, 1128 cm^{-1}; HRMS (ESI) *m/z*: $[M+Na]^+$ calcd for $C_{17}H_{21}NNaO_4$ 326.1368, found 326.1367.

Methyl 3-(pyridine-2-yl)propanoate (3sa)

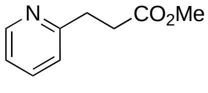

The title compound was obtained from 2-iodopyridine (205 mg, 1.0 mmol) and methyl acrylate (431 mg, 5.0 mmol). Silica gel column chromatography (hexane/AcOEt = 4:1) gave **3sa** (127.9 mg, 0.774 mmol, 77%) as a yellow oil. ^{1}H NMR (400 MHz, $CDCl_3$): δ 8.53 (ddd, J = 4.9, 1.7, 0.9 Hz, 1H), 7.60 (td, J = 7.7, 1.8 Hz, 1H), 7.19 (d, J = 7.8 Hz, 1H), 7.12 (ddd, J = 7.5, 4.9, 0.9 Hz, 1H), 3.67 (s, 3H), 3.12 (t, J = 7.5 Hz, 2H), 2.82 (t, J = 7.5 Hz, 2H); ^{13}C{^{1}H} NMR (100 MHz, $CDCl_3$): δ 173.4, 159.8, 149.2, 136.3, 122.9, 121.3, 51.5, 33.1, 32.7.
The ^{1}H and ^{13}C{^{1}H} NMR spectra are in accordance with those reported in the literature.[8]

Methyl 3-(2-methyl-4-oxo-3-(o-tolyl)-3,4-dihydroquinazolin-6-yl)propanoate (3ta)

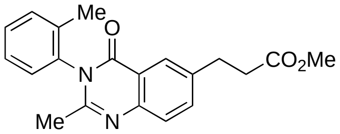

The title compound was obtained from 6-iodo-2-methyl-3-(*o*-tolyl)quinazolin-4(3*H*)-one (376 mg, 1.0 mmol) and methyl acrylate (431 mg, 5.0 mmol). Silica gel column chromatography (hexane/AcOEt = 2:1, then benzene/AcOEt = 4/1) gave **3ta** (180.0 mg, 0.535 mmol, 54%) as a yellow oil. ^{1}H NMR (400 MHz, $CDCl_3$): δ 8.10 (d, J = 0.3 Hz, 1H), 7.66-7.61 (m, 2H), 7.44-7.35 (m, 3H), 7.15 (d, J = 7.3 Hz, 1H), 3.68 (s, 3H), 3.10 (t, J = 7.7 Hz, 2H), 2.71 (t, J = 7.7 Hz, 2H), 2.17 (s, 3H), 2.13 (s, 3H); ^{13}C{^{1}H} NMR (100 MHz, $CDCl_3$): δ 172.9, 161.5, 153.8, 146.2, 139.2, 136.8, 135.3, 135.2, 131.5, 129.5, 127.8, 127.6, 126.9, 125.9, 120.6, 51.7, 35.3, 30.5, 23.8, 17.3; IR (ATR): 2951, 1732, 1676, 1597, 1487, 1269, 1200, 1165 cm^{-1}; HRMS (ESI) *m/z*: [M+Na]$^+$ calcd for $C_{20}H_{20}N_2NaO_3$ 359.1372, found 359.1379.

Methyl 4-(3-(tert-butoxy)-3-oxopropyl)benzoate (3ab)

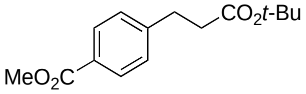

The title compound was obtained from methyl 4-chlorobenzoate (171 mg, 1.0 mmol) and *tert*-butyl acrylate (449 mg, 3.5 mmol). Silica gel column chromatography (hexane/AcOEt = 8:1) gave **3ab** (206.7 mg, 0.782 mmol, 78%) as a colorless oil. ^{1}H NMR (400 MHz, $CDCl_3$): δ 7.97-7.94 (m, 2H), 7.27 (d, J = 8.2 Hz, 5H), 3.90 (s, 3H), 2.96 (t, J = 7.7 Hz, 2H), 2.56 (t, J = 7.7 Hz, 2H), 1.41 (s, 9H); ^{13}C{^{1}H} NMR (100 MHz, $CDCl_3$): δ 171.8, 167.0, 146.2, 129.7, 128.3, 128.1, 80.5, 51.9, 36.4, 31.0, 28.0; IR (ATR): 2978, 1719, 1611, 1435, 1366, 1275, 1144, 1103 cm^{-1}; HRMS (ESI) *m/z*: [M+Na]$^+$ calcd for $C_{15}H_{20}NaO_4$ 287.1259, found 287.1252.
The ^{1}H NMR spectrum is in accordance with those reported in the literature.[9]

Methyl 4-(3-amino-2-methyl-3-oxopropyl)benzoate (3ac)

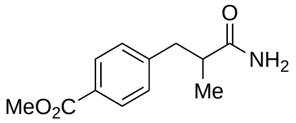

The title compound was obtained from methyl 4-chlorobenzoate (171 mg, 1.0 mmol) and methacrylamide (298 mg, 3.5 mmol) with 5 F/mol of electricity. Silica gel column chromatography (AcOEt 100%) gave **3ac** (175.0 mg, 0.791 mmol, 79%) as a white solid. mp 146–147 °C; ^{1}H NMR (400 MHz, $CDCl_3$): δ 7.96 (d, J = 8.2 Hz, 2H), 7.27 (d, J = 8.2 Hz, 2H), 5.25 (br s, 2H), 3.90 (s, 3H), 3.06 (dd, J = 13.5, 8.0 Hz, 1H), 2.74 (dd, J = 13.5, 6.6 Hz, 1H), 2.53 (dqd, J = 8.0, 6.9, 6.6 Hz, 1H), 1.21 (d, J = 6.9 Hz, 3H); ^{13}C{^{1}H} NMR (100 MHz, $CDCl_3$): δ 177.7, 167.0, 145.1, 129.7, 129.0, 128.3, 52.0, 42.6, 40.0, 17.7; IR (ATR): 3410, 3194, 2974, 1682, 1661, 1287, 1179, 1115 cm^{-1}; HRMS (ESI) *m/z*: [M+Na]$^+$ calcd for $C_{12}H_{15}NNaO_3$ 244.0950, found 244.0945.

Methyl 4-(2-cyanoethyl)benzoate (3ad)

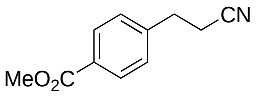

The title compound was obtained from methyl 4-chlorobenzoate (171 mg, 1.0 mmol) and acrylonitrile

(186 mg, 3.5 mmol) with 4.5 F/mol of electricity. Silica gel column chromatography (hexane/AcOEt = 4:1) gave **3ad** (154.6 mg, 0.817 mmol, 82%) as a white solid. ^{1}H NMR (400 MHz, $CDCl_3$): δ 8.03-8.01 (m, 2H), 7.32 (d, J = 8.2 Hz, 2H), 3.92 (s, 3H), 3.02 (t, J = 7.3 Hz, 2H), 2.66 (t, J = 7.3 Hz, 2H); ^{13}C{^{1}H} NMR (100 MHz, $CDCl_3$): δ 166.5, 143.0, 129.9, 128.9, 128.2, 118.6, 51.9, 31.2, 18.7.
The ^{1}H and ^{13}C{^{1}H} NMR spectra are in accordance with those reported in the literature.[10]

Methyl 4-(2,2-diphenylethyl)benzoate (3ae)

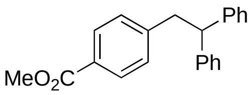

The title compound was obtained from methyl 4-chlorobenzoate (171 mg, 1.0 mmol) and 1,1-diphenylethylene (631 mg, 3.5 mmol). Silica gel column chromatography (hexane/AcOEt = 60:1) gave **3ae** (250.8 mg, 0.793 mmol, 79%) as a white solid. ^{1}H NMR (400 MHz, $CDCl_3$): δ 7.85-7.82 (m, 2H), 7.27-7.23 (m, 4H), 7.20-7.14 (m, 6H), 7.06 (d, J = 8.5 Hz, 2H), 4.23 (t, J = 7.9 Hz, 1H), 3.87 (s, 3H), 3.41 (d, J = 7.8 Hz, 2H); ^{13}C{^{1}H} NMR (100 MHz, $CDCl_3$): δ 166.9, 145.6, 143.8, 129.3, 129.0, 128.3, 127.8, 127.7, 126.2, 52.7, 51.8, 41.9.
The ^{1}H and ^{13}C{^{1}H} NMR spectra are in accordance with those reported in the literature.[11]

Methyl 4-(2-phenylpropyl)benzoate (3af)

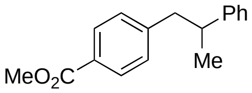

The title compound was obtained from methyl 4-chlorobenzoate (171 mg, 1.0 mmol) and α-methylstyrene (414 mg, 3.5 mmol). Silica gel column chromatography (hexane/AcOEt = 80:1) gave **3af** (200.9 mg, 0.790 mmol, 79%) as a colorless oil. ^{1}H NMR (400 MHz, $CDCl_3$): δ 7.89 (d, J = 8.0 Hz, 2H), 7.29-7.25 (m, 2H), 7.20-7.11 (m, 5H), 3.89 (s, 3H), 3.05-2.94 (m, 2H), 2.85 (dd, J = 12.8, 7.5 Hz, 1H), 1.26 (d, J = 6.6 Hz, 3H); ^{13}C{^{1}H} NMR (100 MHz, $CDCl_3$): δ 166.9, 146.1, 146.0, 129.3, 129.0, 128.2, 127.7, 126.8, 126.0, 51.7, 44.8, 41.5, 21.1; IR (ATR): 3028, 2855, 1717, 1609, 1433, 1275, 1179, 1105 cm^{-1}; HRMS (ESI) *m/z*: $[M+Na]^+$ calcd for $C_{17}H_{18}NaO_2$ 277.1205, found 277.1207.

Methyl 4-(2-phenylethyl)benzoate (3ag)

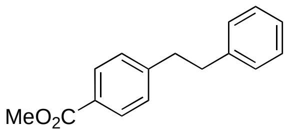

The title compound was obtained from methyl 4-chlorobenzoate (171 mg, 1.0 mmol) and styrene (521 mg, 5.0 mmol) using MeCN (3.0 mL). Silica gel column chromatography (hexane/AcOEt = 80:1) gave **3ag** (183.6 mg, 0.764 mmol, 76%) as a white solid. ^{1}H NMR (400 MHz, $CDCl_3$): δ 7.96-7.93 (m, 2H), 7.29-7.14 (m, 7H), 3.90 (s, 3H), 3.01-2.91 (m, 4H); ^{13}C{^{1}H} NMR (100 MHz, $CDCl_3$): δ 167.0, 147.0, 141.0, 129.6, 128.4, 128.3, 128.3, 127.8, 126.0, 51.8, 37.7, 37.3.
The ^{1}H and ^{13}C{^{1}H} NMR spectra are in accordance with those reported in the literature.[12]

Methyl 4-(2-(4-methoxyphenyl)ethyl)benzoate (3ah)

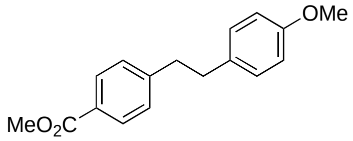

The title compound was obtained from methyl 4-chlorobenzoate (171 mg, 1.0 mmol) and 4-methoxystyrene (470 mg, 3.5 mmol) using MeCN (3.0 mL). Silica gel column chromatography (hexane/AcOEt = 20:1) gave **3ah** (165.2 mg, 0.611 mmol, 61%) as a white solid. ^{1}H NMR (400 MHz, $CDCl_3$): δ 7.95-7.92 (m, 2H), 7.21 (d, J = 8.2 Hz, 2H), 7.07-7.04 (m, 2H), 6.83-6.79 (m, 2H), 3.90 (s, 3H), 3.79 (s, 3H), 2.96-2.85 (m, 4H); ^{13}C{^{1}H} NMR (100 MHz, $CDCl_3$): δ 167.1, 157.8, 147.2, 133.1, 129.6, 129.3, 128.5, 127.8, 113.7, 55.1, 51.9, 38.1, 36.5.

The ^{1}H and ^{13}C{^{1}H} NMR spectra are in accordance with those reported in the literature.[12]

Methyl 4-(2-(4-chlorophenyl)ethyl)benzoate (3ai)

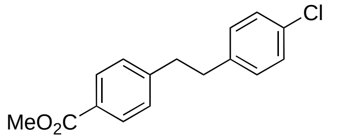

The title compound was obtained from methyl 4-chlorobenzoate (171 mg, 1.0 mmol) and 4-chlorostyrene (485 mg, 3.5 mmol) using MeCN (3.0 mL). Silica gel column chromatography (hexane/AcOEt = 80:1) gave **3ai** (234.7 mg, 0.854 mmol, 85%) as a white solid. ^{1}H NMR (400 MHz, $CDCl_3$): δ 7.96-7.93 (m, 2H), 7.24-7.18 (m, 4H), 7.07-7.04 (m, 2H), 3.91 (s, 3H), 2.97-2.88 (m, 4H); ^{13}C{^{1}H} NMR (100 MHz, $CDCl_3$): δ 167.0, 146.6, 139.4, 131.7, 129.8, 129.6, 128.5, 128.4, 128.0, 52.0, 37.6, 36.7.

The ^{1}H and ^{13}C{^{1}H} NMR spectra are in accordance with those reported in the literature.[13]

Methyl 4-(2,3-dihydro-1H-inden-2-yl)benzoate (3aj)

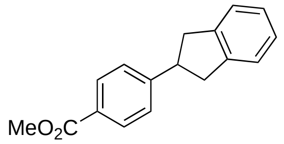

The title compound was obtained from methyl 4-chlorobenzoate (171 mg, 1.0 mmol) and 1*H*-indene (581 mg, 5.0 mmol) using MeCN (3.0 mL). Silica gel column chromatography (hexane/AcOEt = 80:1) gave **3aj** (140.2 mg, 0.556 mmol, 56%) as a yellow oil. ^{1}H NMR (400 MHz, $CDCl_3$): δ 7.99-7.96 (m, 2H), 7.38-7.35 (m, 2H), 7.26-7.23 (m, 2H), 7.22-7.17 (m, 2H), 3.91 (s, 3H), 3.79-3.70 (m, 1H), 3.38 (dd, J = 15.3, 8.2 Hz, 2H), 3.09 (dd, J = 15.4, 8.6 Hz, 2H); ^{13}C{^{1}H} NMR (100 MHz, $CDCl_3$): δ 166.9, 150.8, 142.4, 129.7, 128.0, 126.9, 126.5, 124.2, 51.8, 45.2, 40.5.

The ^{1}H and ^{13}C{^{1}H} NMR spectra are in accordance with those reported in the literature.[14]

Methyl 4-(2-(pyridin-2-yl)ethyl)benzoate (3ak)

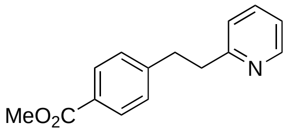

The title compound was obtained from methyl 4-chlorobenzoate (171 mg, 1.0 mmol) and 2-vinylpyridine (526 mg, 5.0 mmol) using MeCN (3.0 mL). Silica gel column chromatography (hexane/AcOEt = 5:1) gave **3ak** (199.3 mg, 0.826 mmol, 83%) as a white solid. mp 70–71 °C; ^{1}H NMR (400 MHz, $CDCl_3$): δ 8.56 (ddd, J = 4.9, 1.8, 0.9 Hz, 1H), 7.95-7.92 (m, 2H), 7.56 (td, J = 7.7, 1.8 Hz, 1H), 7.26-7.24 (m, 2H), 7.12 (ddd, J = 7.6, 4.9, 1.1 Hz, 1H), 7.04 (dt, J = 7.8, 1.0 Hz, 1H), 3.90 (s, 3H), 3.16-3.07 (m, 4H); ^{13}C{^{1}H} NMR (100 MHz, $CDCl_3$): δ 167.0, 160.5, 149.3, 147.0, 136.3, 129.6, 128.4, 127.8, 122.9, 121.2, 51.9, 39.6, 35.8.; IR (ATR): 2943, 1707, 1589, 1439, 1281, 1107 cm^{-1}; HRMS (ESI) *m/z*: $[M+H]^+$ calcd for $C_{15}H_{16}NO_2$ 242.1181, found 242.1178.
The ^{1}H and ^{13}C{^{1}H} NMR spectra are in accordance with those reported in the literature.[15]

3-Phenylpropanenitrile (3kd)

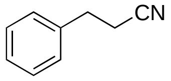

The title compound was obtained from iodobenzene (204 mg, 1.0 mmol) and acrylonitrile (265 mg, 5.0 mmol). Silica gel column chromatography (hexane/AcOEt = 8:1) gave **3kd** (111.8 mg, 0.852 mmol, 85%) as a colorless oil. ^{1}H NMR (400 MHz, $CDCl_3$): δ 7.37-7.33 (m, 2H), 7.30-7.23 (m, 3H), 2.97 (t, J = 7.4 Hz, 2H), 2.63 (t, J = 7.4 Hz, 2H); ^{13}C{^{1}H} NMR (100 MHz, $CDCl_3$): δ 137.9, 128.7, 128.1, 127.0, 119.0, 31.3, 19.1.
The ^{1}H and ^{13}C{^{1}H} NMR spectra are in accordance with those reported in the literature.[16]

1-Chloro-4-(2-phenylethyl)benzene (3ki)

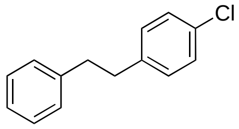

The title compound was obtained from iodobenzene (204 mg, 1.0 mmol) and 4-chlorostyrene (693 mg, 5.0 mmol). Silica gel column chromatography (hexane 100%) gave **3ki** (158.6 mg, 0.732 mmol, 73%) as a white solid. ^{1}H NMR (400 MHz, $CDCl_3$): δ 7.30-7.14 (m, 7H), 7.10-7.06 (m, 2H), 2.89 (s, 4H); ^{13}C{^{1}H} NMR (100 MHz, $CDCl_3$): δ 141.2, 140.1, 131.6, 129.8, 128.42, 128.35, 128.3, 126.0, 37.7, 37.2.
The ^{1}H and ^{13}C{^{1}H} NMR spectra are in accordance with those reported in the literature.[17]

2.3. Gram-scale experiment

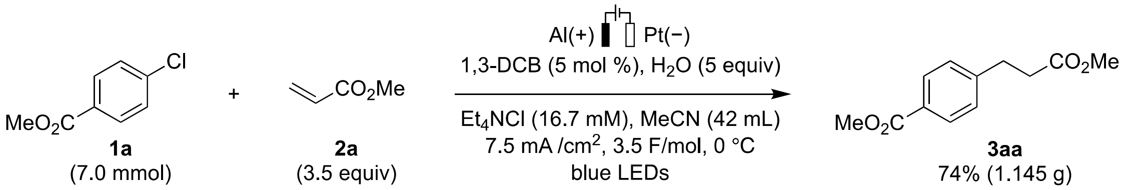

A cylinder-type undivided cell with a stir bar was charged with Et_4NCl (116 mg, 0.7 mmol), 1,3-dicyanobenzene (1,3-DCB, 44.8 mg, 0.35 mmol), **2a** (2.11 g, 24.5 mmol), and **1a** (1.19 g, 7.0 mmol). Anhydrous MeCN (42 mL) and H_2O (0.63 mL) were successively added. The reaction vessel was equipped with seven Pt plate cathodes (immersed surface area: 1×2 cm^2 each) and seven Al plate anodes (immersed surface area: 1×2 cm^2 each) under air (Figure S2), then 3.5 F/mol of electricity was passed through at a constant current condition (7.5 mA/cm^2) under visible-light irradiation (approximate distance from the cathodes: 2.5 cm) at 0 °C in an ice bath (Figure S3). Most of solvent was removed under reduced pressure, and the residue was diluted with AcOEt. The organic layer was washed with H_2O, dried over Na_2SO_4, filtered, and concentrated under reduced pressure. The residue was purified by flash silica gel column chromatography (hexane/AcOEt = 8/1, then benzene 100%) to afford **3aa** (1.145 g, 5.15 mmol, 74 % yield).

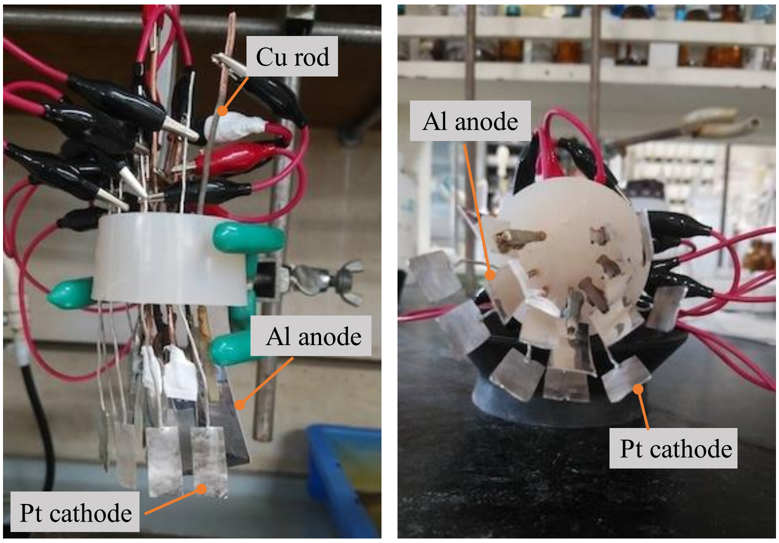

Figure S2. Side view (left) and bottom view (right) of a silicone stopper equipped with Al and Pt electrodes used for the gram-scale reaction.

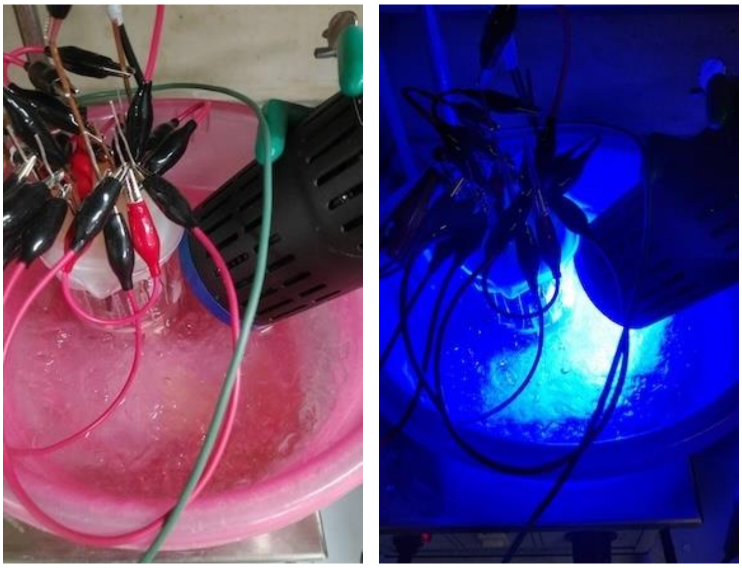

Figure S3. A reaction setup for the gram-scale reaction.

2.4. Radical-clock experiment

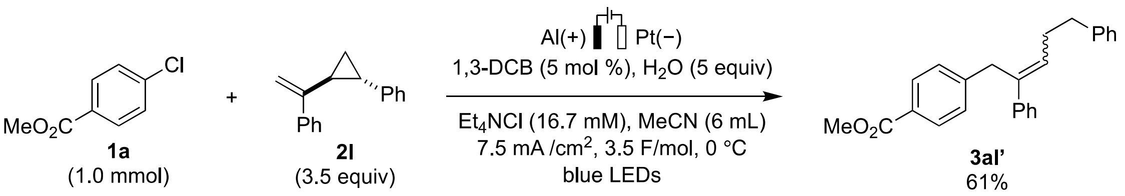

A cylinder-type undivided cell with a stir bar was charged with Et_4NCl (16.6 mg, 0.1 mmol), 1,3-dicyanobenzene (1,3-DCB, 6.4 mg, 0.05 mmol), **2l** (771 mg, 3.5 mmol), and **1a** (170 mg, 1.0 mmol). Anhydrous MeCN (6.0 mL) and H_2O (90 μL) were successively added. The reaction vessel was equipped with a Pt plate cathode (immersed surface area: 1 × 2 cm^2) and an Al plate anode (immersed surface area: 1 × 2 cm^2) under air, then 3.5 F/mol of electricity was passed through at a constant current condition (7.5 mA/cm^2) under visible-light irradiation (approximate distance from the cathode: 2.5 cm) at 0 °C in an ice bath. Most of solvent was removed under reduced pressure, and the residue was diluted with AcOEt. The organic layer was washed with H_2O, dried over Na_2SO_4, filtered, and concentrated under reduced pressure. The residue was purified by flash silica gel column chromatography (hexane/AcOEt = 40:1) gave **3al'** (216.6 mg, 0.608 mmol, 61%).

Methyl 4-(2,5-diphenylpent-2-en-1-yl)benzoate (3al')

Colorless oil; ^1H NMR (400 MHz, $CDCl_3$): δ 7.86 (d, J = 8.0 Hz, 2H), 7.31-7.12 (m, 12H), 6.02 (t, J = 7.3 Hz, 1H), 3.87 (s, 3H), 3.84 (s, 2H), 2.78 (t, J = 7.5 Hz, 2H), 2.56 (td, J = 7.5, 7.3 Hz, 2H); $^{13}C\{^1H\}$ NMR (100 MHz, $CDCl_3$): δ 166.9, 145.2, 142.3, 141.4, 137.3, 130.2, 129.6, 128.4, 128.3, 128.12, 128.06, 127.7, 126.7, 126.1, 125.9, 51.8, 35.69, 35.67, 30.9; IR (ATR): 3024, 2947, 1717, 1609, 1495, 1275, 1177, 1105 cm^{-1}; HRMS (ESI) m/z: $[M+Na]^+$ calcd for $C_{25}H_{24}NaO_2$ 379.1674, found 379.1672.

2.5. Deuterium-labeling experiment

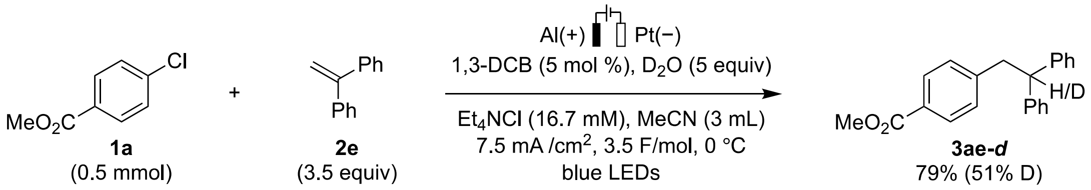

A cylinder-type undivided cell with a stir bar was charged with Et_4NCl (8.3 mg, 0.05 mmol), 1,3-dicyanobenzene (1,3-DCB, 3.2 mg, 0.025 mmol), **2e** (315 mg, 1.75 mmol), and **1a** (85 mg, 0.5 mmol). Anhydrous MeCN (3.0 mL) and D_2O (45 µL) were successively added. The reaction vessel was equipped with a Pt plate cathode (immersed surface area: 1 × 2 cm^2) and an Al plate anode (immersed surface area: 1 × 2 cm^2) under air, then 3.5 F/mol of electricity was passed through at a constant current condition (7.5 mA/cm^2) under visible-light irradiation (approximate distance from the cathode: 2.5 cm) at 0 °C in an ice bath. Most of solvent was removed under reduced pressure, and the residue was diluted with AcOEt. The organic layer was washed with H_2O, dried over Na_2SO_4, filtered, and concentrated under reduced pressure. The residue was purified by flash silica gel column chromatography to afford the desired product **3ae-*d*** (124.9 mg, 0.394 mmol, 79% yield, 51% D).

Compound 3ae-d (51% D)

^{1}H NMR (400 MHz, $CDCl_3$): δ 7.83 (d, J = 8.2 Hz, 2H), 7.26-7.23 (m, 5H), 7.19-7.14 (m, 6H), 7.06 (d, J = 8.0 Hz, 2H), 4.23 (t, J = 7.9 Hz, 0.49H), 3.87 (s, 3H), 3.41 (d, J = 6.6 Hz, 2H); $^{13}C\{^1H\}$ NMR (100 MHz, $CDCl_3$): δ 167.0, 145.7, 143.9, 143.8, 129.4, 129.1, 128.4, 127.88, 127.87, 127.8, 126.3, 77.3, 77.0, 76.7, 52.8, 52.3 (t, J = 19.8 Hz), 51.9, 42.04, 41.96; IR (ATR): 3024, 2926, 1713, 1609, 1491, 1279, 1179, 1103 cm^{-1}; HRMS (ESI) m/z: $[M+Na]^+$ calcd for $C_{22}H_{19}{}^2HNaO_2$ 340.1424, found 340.1439.

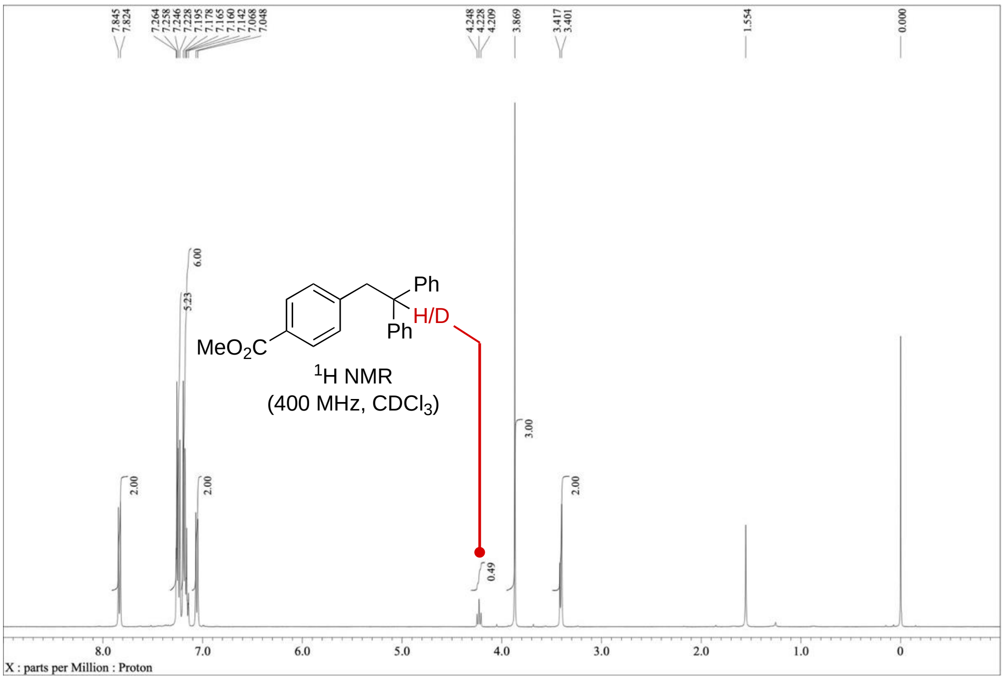

3. References

1) Amatore, M.; Gosmini, C.; Périchon, J. *J. Org. Chem.* **2006**, *71*, 6130–6134.
2) Leow, D.; Chen, Y.-H.; Hung, T.-H.; Su, Y.; Lin, Y.-Z. *Eur. J. Org. Chem.* **2014**, *2014*, 7347–7352.
3) Shavnya, A.; Coffey, S. A.; Smith, A. C.; Mascitti, V. *Org. Lett.* **2013**, *15*, 6226–6229.
4) Shibuya, M.; Shibuta, T.; Fukuda, H.; Iwabuchi, Y. *Org. Lett.* **2012**, *14*, 5010–5013.
5) Yao, L.; Mustafa, N.; Tan, E. C.; Poulsen, A.; Singh, P.; Duong-Thi, M.-D.; Lee, J. X. T.; Ramanujulu, P. M.; Chng, W. J.; Yen, J. J. Y.; Ohlson, S.; Dymock, B. W. *J. Med. Chem.* **2017**, *60*, 8336–8357.
6) Liu, J.-B.; Chen, C.; Chu, L.; Chen, Z.-H.; Xu, X.-H.; Qing, F.-L. *Angew. Chem., Int. Ed.* **2015**, *54*, 11839–11842.
7) Vieira, T. O.; Green, M. J.; Alper, H. *Org. Lett.* **2006**, *8*, 6143–6145.
8) Bon, D. J.-Y. D.; Kováč, O.; Ferugová, V.; Zálešák, F.; Pospíšil, J. *J. Org. Chem.* **2018**, *83*, 4990–5001.
9) Yoon, S. H.; Joo, H. W.; Seo, B. K.; Lee, E. J.; Jung, J. Y.; Yoon, S. Y.; Kwak, Y. S.; Cho, W. Y.; Jo, M. M. *PCT Int. Appl.* WO 2021/133038 A1, 2021.
10) Weweler, J.; Younas, S. L.; Streuff, J. *Angew. Chem., Int. Ed.* **2019**, *58*, 17700–17703.
11) Gao, L.; Wang, G.; Cao, J.; Yuan, D.; Xu, C.; Guo, X.; Li, S. *Chem. Commun.* **2018**, *54*, 11534–11537.
12) del Río-Rodríguez, R.; Blanco, L.; Collado, A.; Fernández-Salas, J. A.; Alemán, J. *Chem. Eur. J.* **2022**, *28*, e202201644.
13) Basnet, P.; Thapa, S.; Dickie, D. A.; Giri, R. *Chem. Commun.* **2016**, *52*, 11072–11075.
14) Zhang, X.; MacMillan, D. W. C. *J. Am. Chem. Soc.* **2016**, *138*, 13862–13865.
15) Cheng, L.; Sun, Y.; Wang, W.; Yao, C.; Li, T.-J. *J. Org. Chem.* **2019**, *84,* 3074–3082.
16) Black, P. J.; Edwards, M. G.; Williams, J. M. J. *Eur. J. Org. Chem.* **2006**, *2006*, 4367–4378.
17) Kantam, M. L.; Chakravarti, R.; Chintareddy, V. R.; Sreedhar, B.; Bhargava, S. *Adv. Synth. Catal.* **2008**, *350*, 2544–2550.

4. ^{1}H, ^{13}C{^{1}H}, and ^{19}F NMR spectra

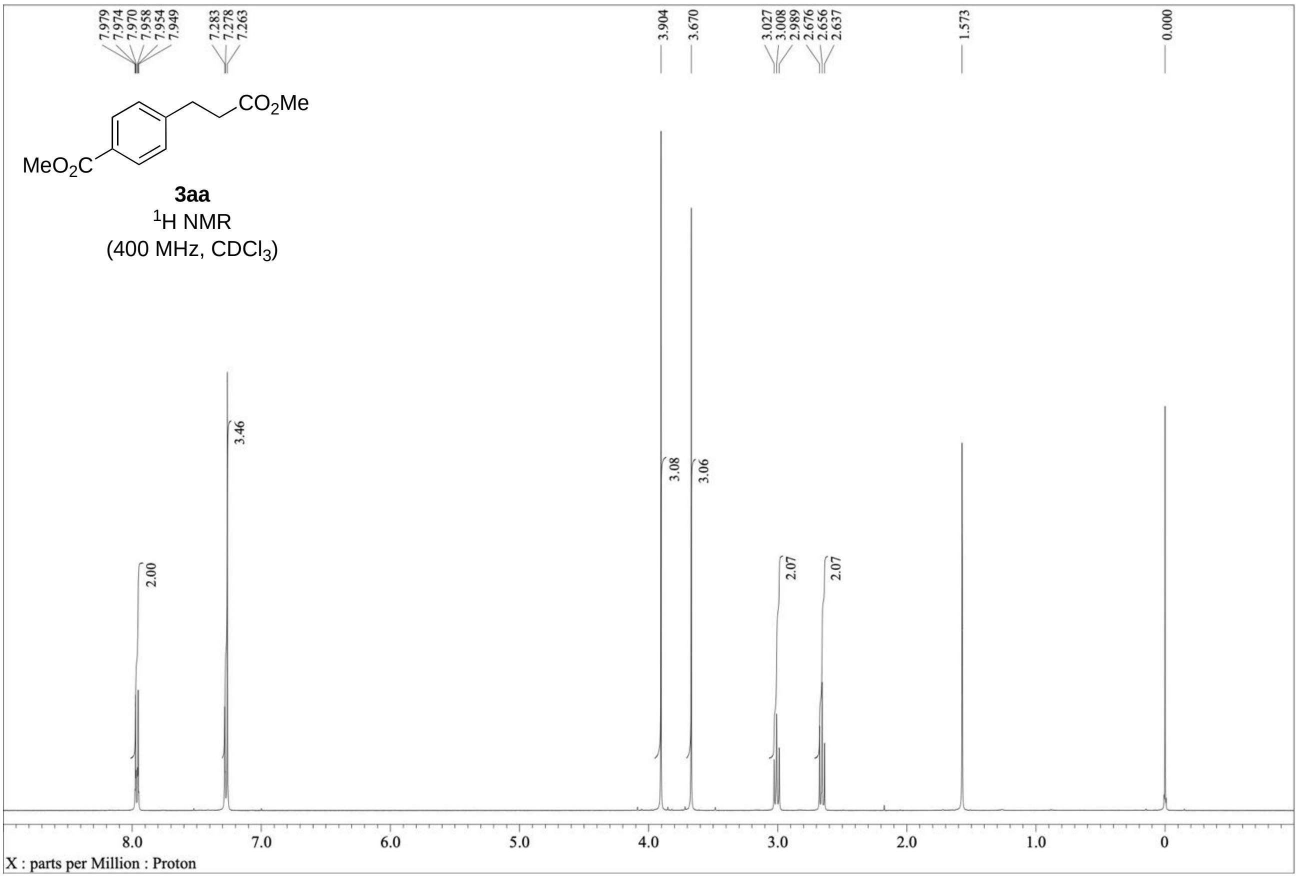

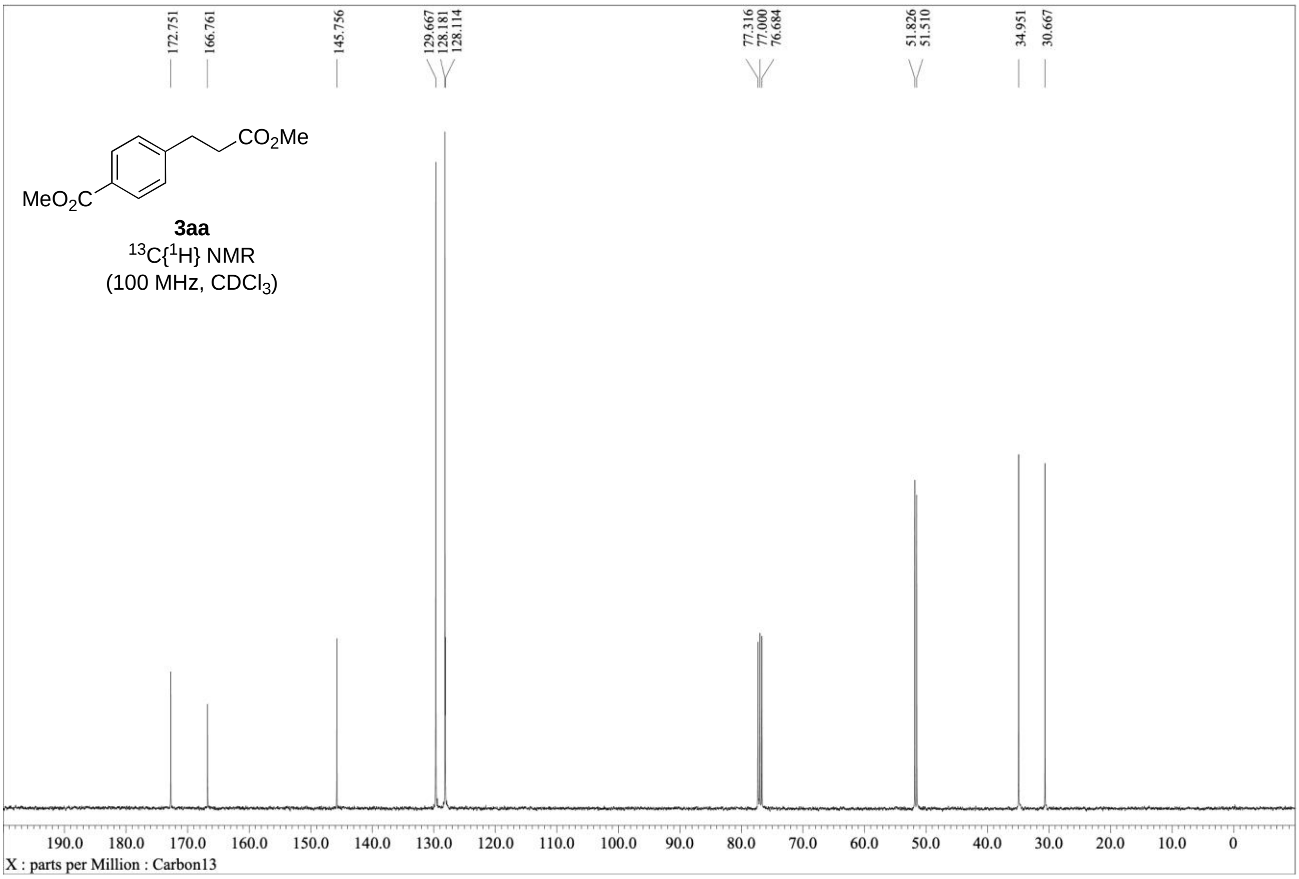

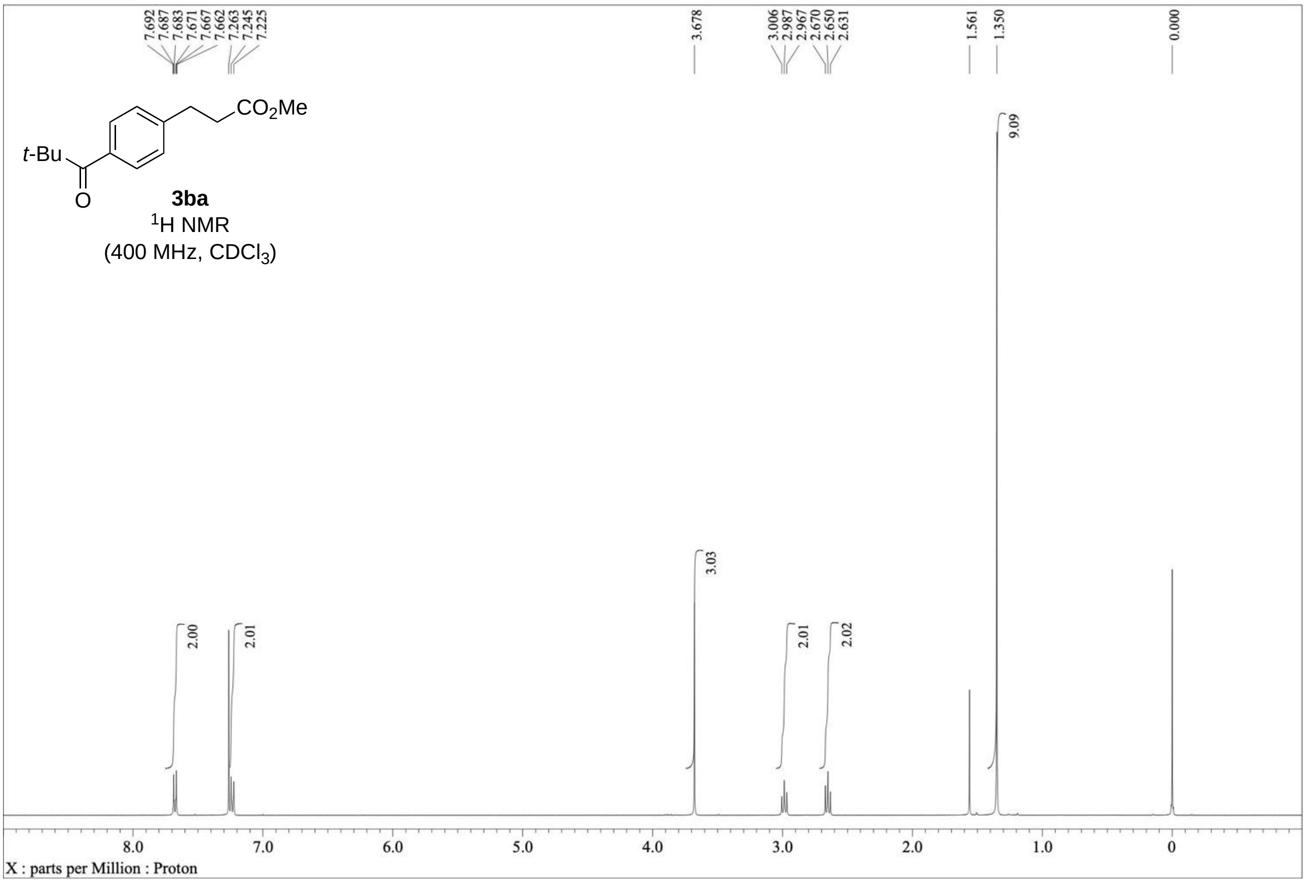
7.692
7.687
7.683
7.671
7.667
7.662
7.263
7.245
7.225
3.678
3.006
2.987
2.967
2.670
2.650
2.631
1.561
1.350
0.000
CO2Me
t-Bu
O
3ba
1H NMR
(400 MHz, CDCl3)
9.09
3.03
2.00
2.01
2.01
2.02
8.0
7.0
6.0
5.0
4.0
3.0
2.0
1.0
0
X : parts per Million : Proton

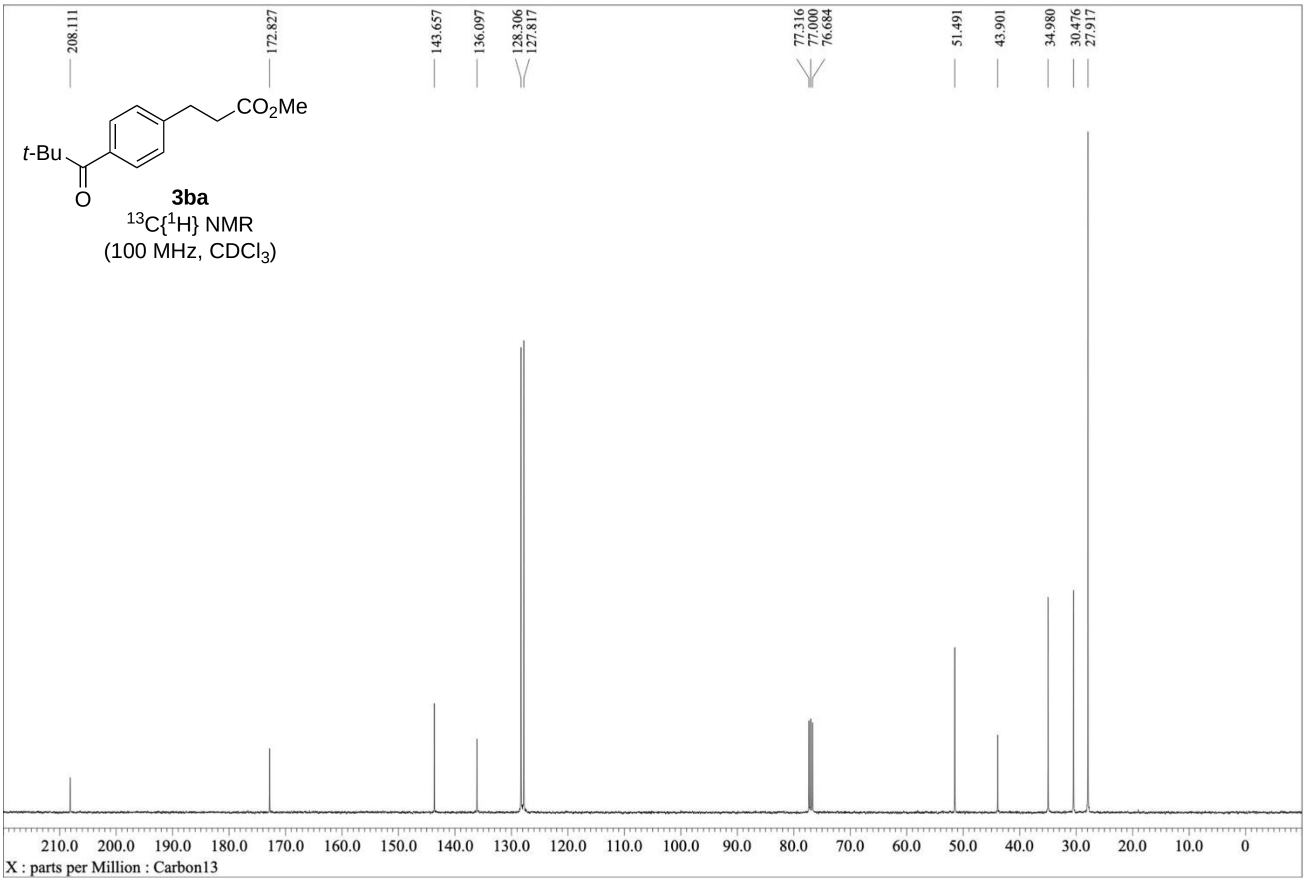
208.111
172.827
143.657
136.097
128.306
127.817
77.316
77.000
76.684
51.491
43.901
34.980
30.476
27.917
CO2Me
t-Bu
O
3ba
13C{1H} NMR
(100 MHz, CDCl3)
210.0
200.0
190.0
180.0
170.0
160.0
150.0
140.0
130.0
120.0
110.0
100.0
90.0
80.0
70.0
60.0
50.0
40.0
30.0
20.0
10.0
0
X : parts per Million : Carbon13

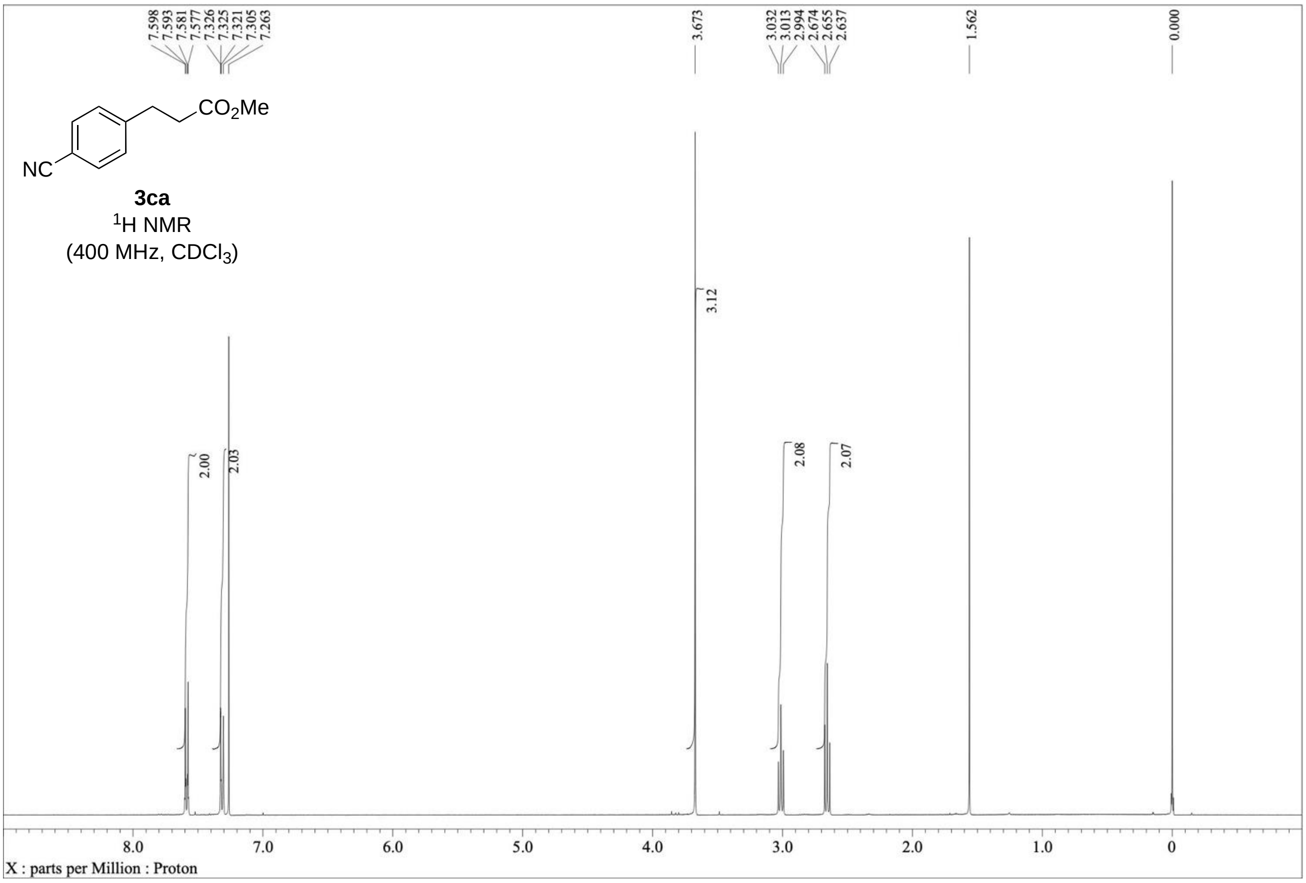

7.598
7.593
7.581
7.577
7.326
7.325
7.321
7.305
7.263
3.673
3.032
3.013
2.994
2.674
2.655
2.637
1.562
0.000
CO2Me
NC
3ca
1H NMR
(400 MHz, CDCl3)
3.12
2.00
2.03
2.08
2.07
8.0
7.0
6.0
5.0
4.0
3.0
2.0
1.0
0
X : parts per Million : Proton

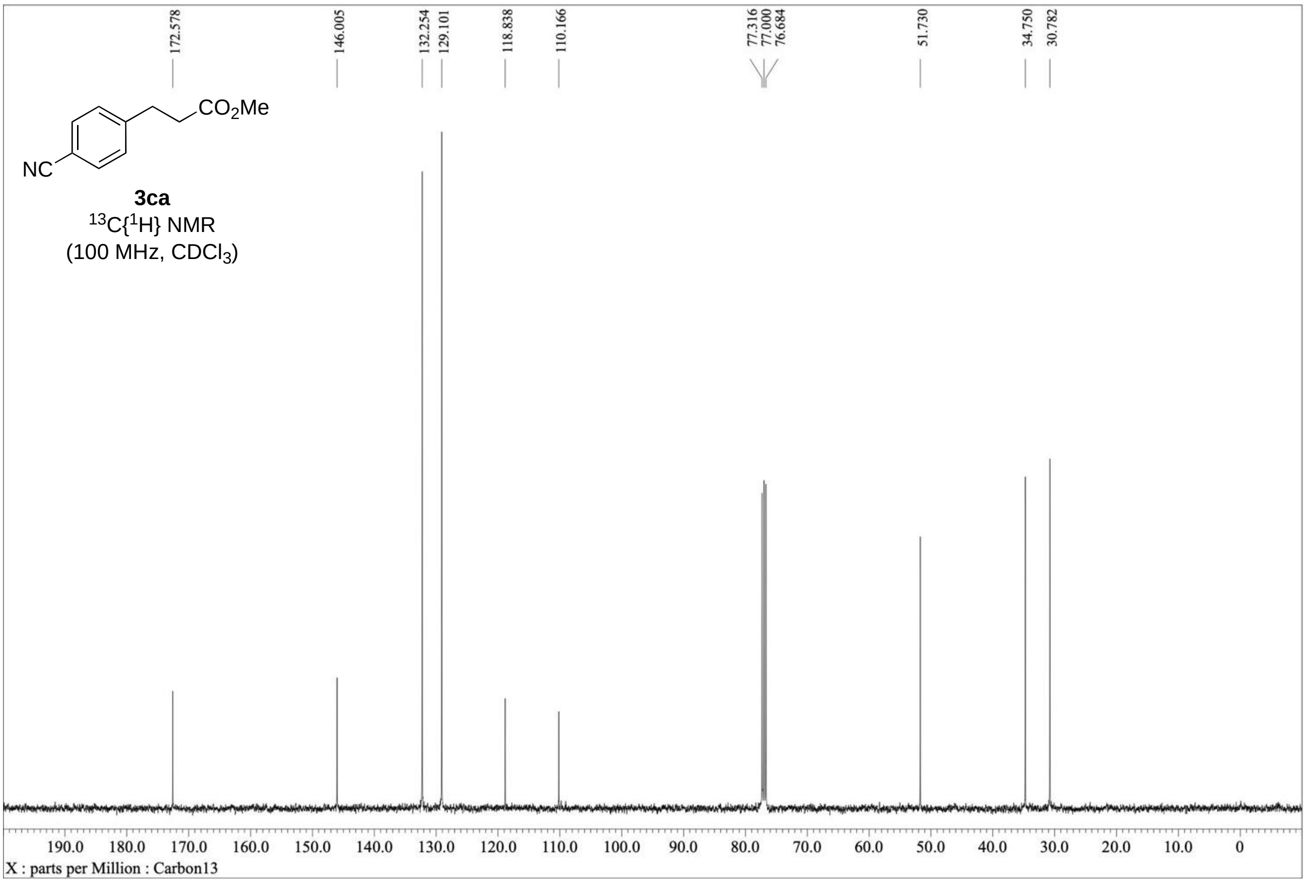

172.578
146.005
132.254
129.101
118.838
110.166
77.316
77.000
76.684
51.730
34.750
30.782
CO2Me
NC
3ca
13C{1H} NMR
(100 MHz, CDCl3)
190.0
180.0
170.0
160.0
150.0
140.0
130.0
120.0
110.0
100.0
90.0
80.0
70.0
60.0
50.0
40.0
30.0
20.0
10.0
0
X : parts per Million : Carbon13

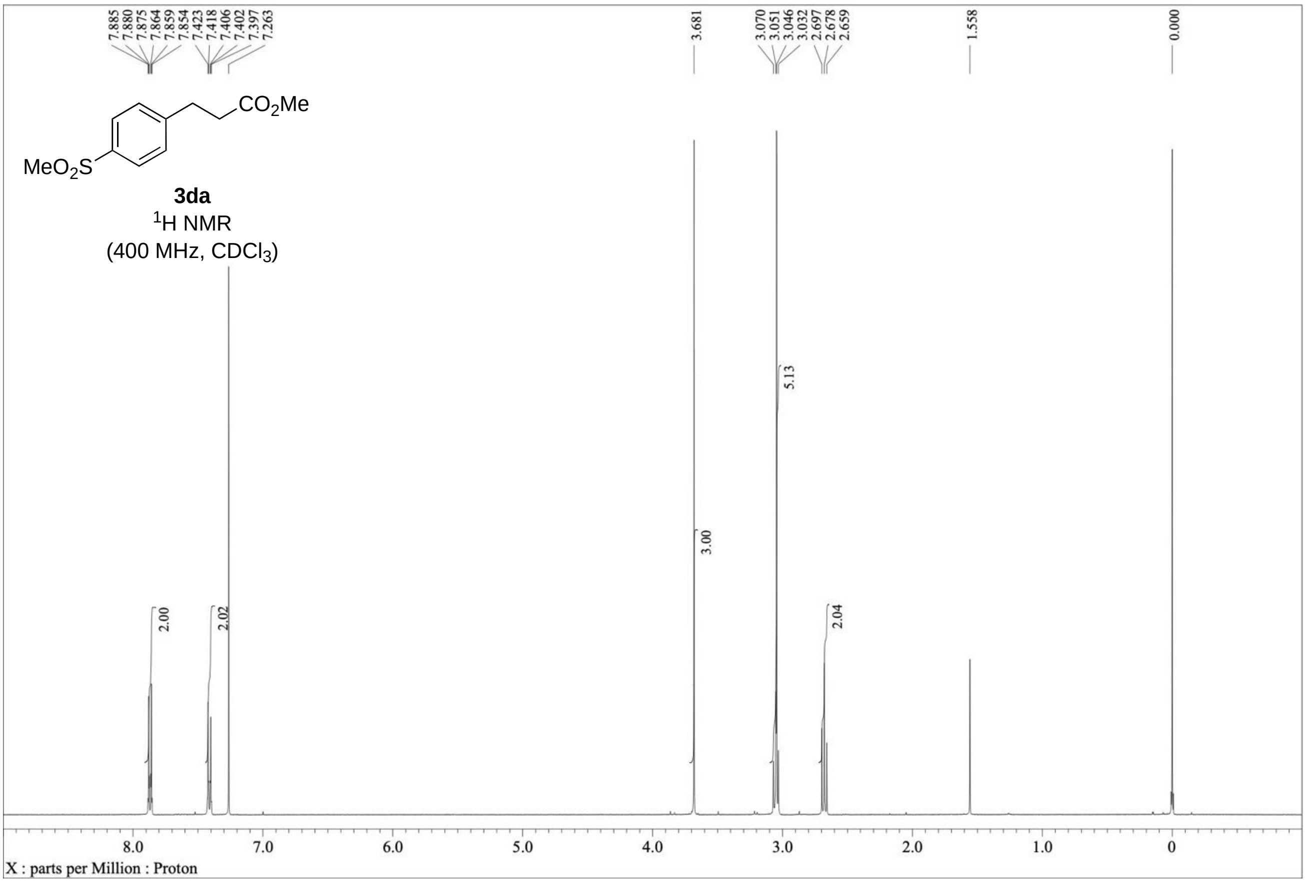
7.885
7.880
7.875
7.864
7.859
7.854
7.423
7.418
7.406
7.402
7.397
7.263
3.681
3.070
3.051
3.046
3.032
2.697
2.678
2.659
1.558
0.000
CO2Me
MeO2S
3da
1H NMR
(400 MHz, CDCl3)
2.00
2.02
3.00
5.13
2.04
X : parts per Million : Proton

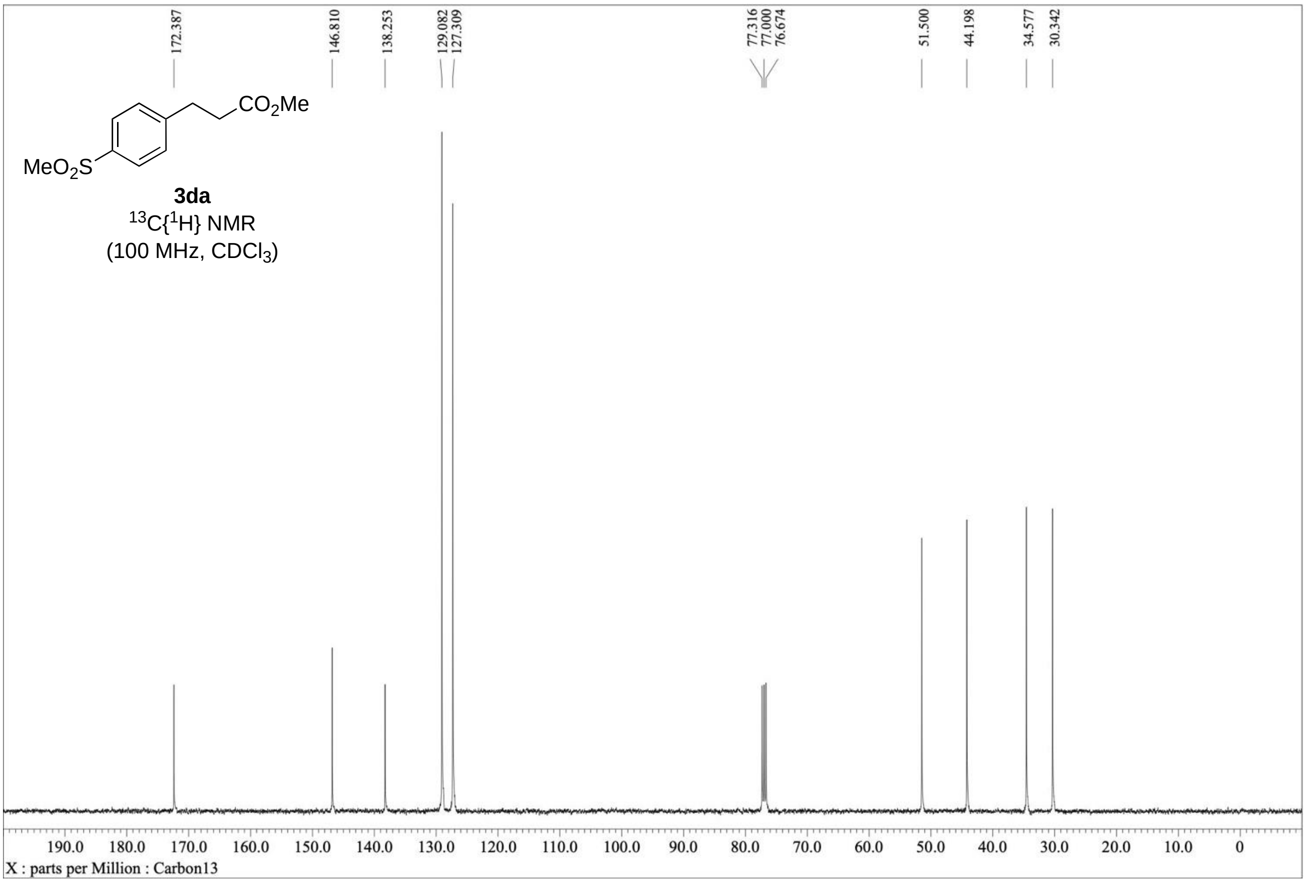
172.387
146.810
138.253
129.082
127.309
77.316
77.000
76.674
51.500
44.198
34.577
30.342
CO2Me
MeO2S
3da
13C{1H} NMR
(100 MHz, CDCl3)
X : parts per Million : Carbon13

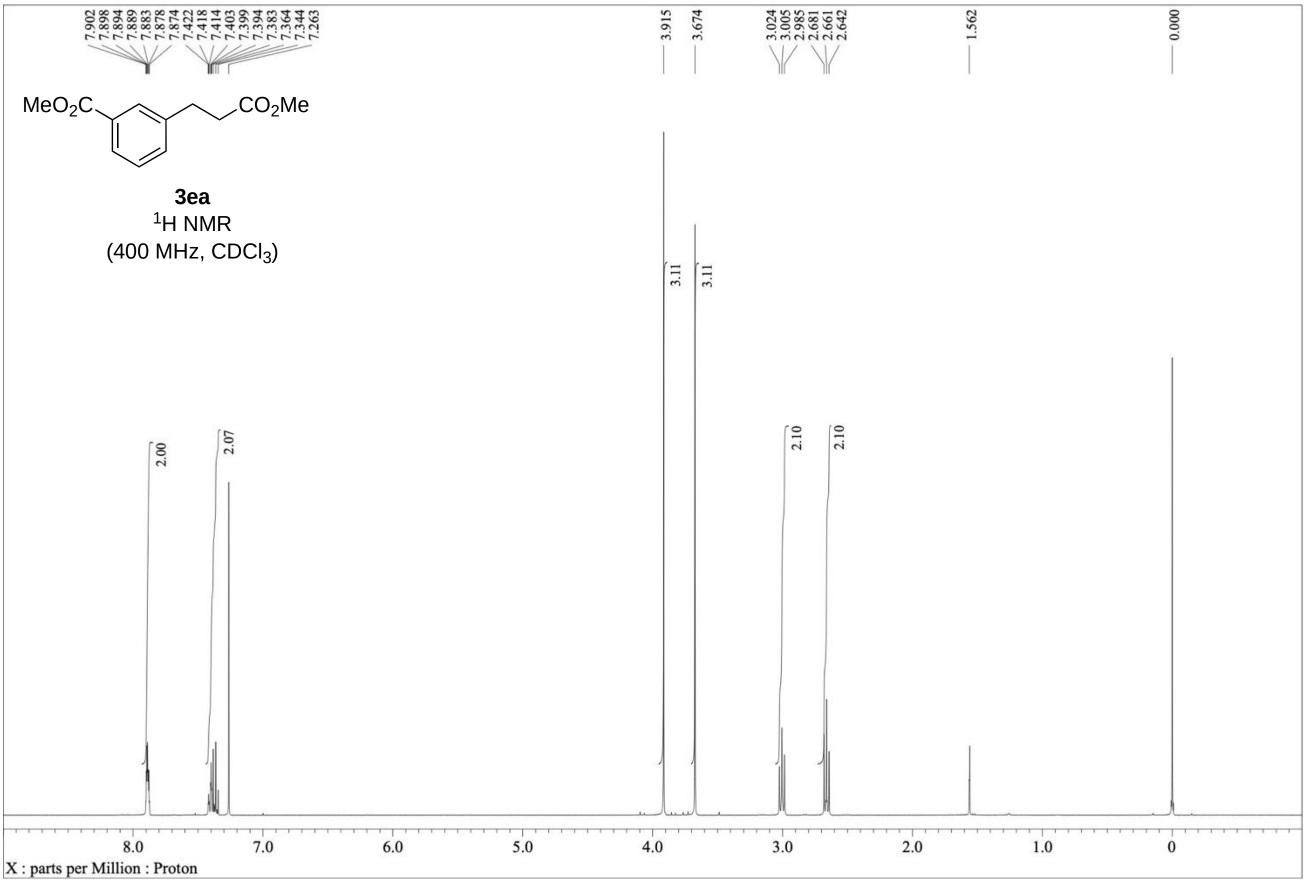
MeO2C
CO2Me
3ea
1H NMR
(400 MHz, CDCl3)
X : parts per Million : Proton

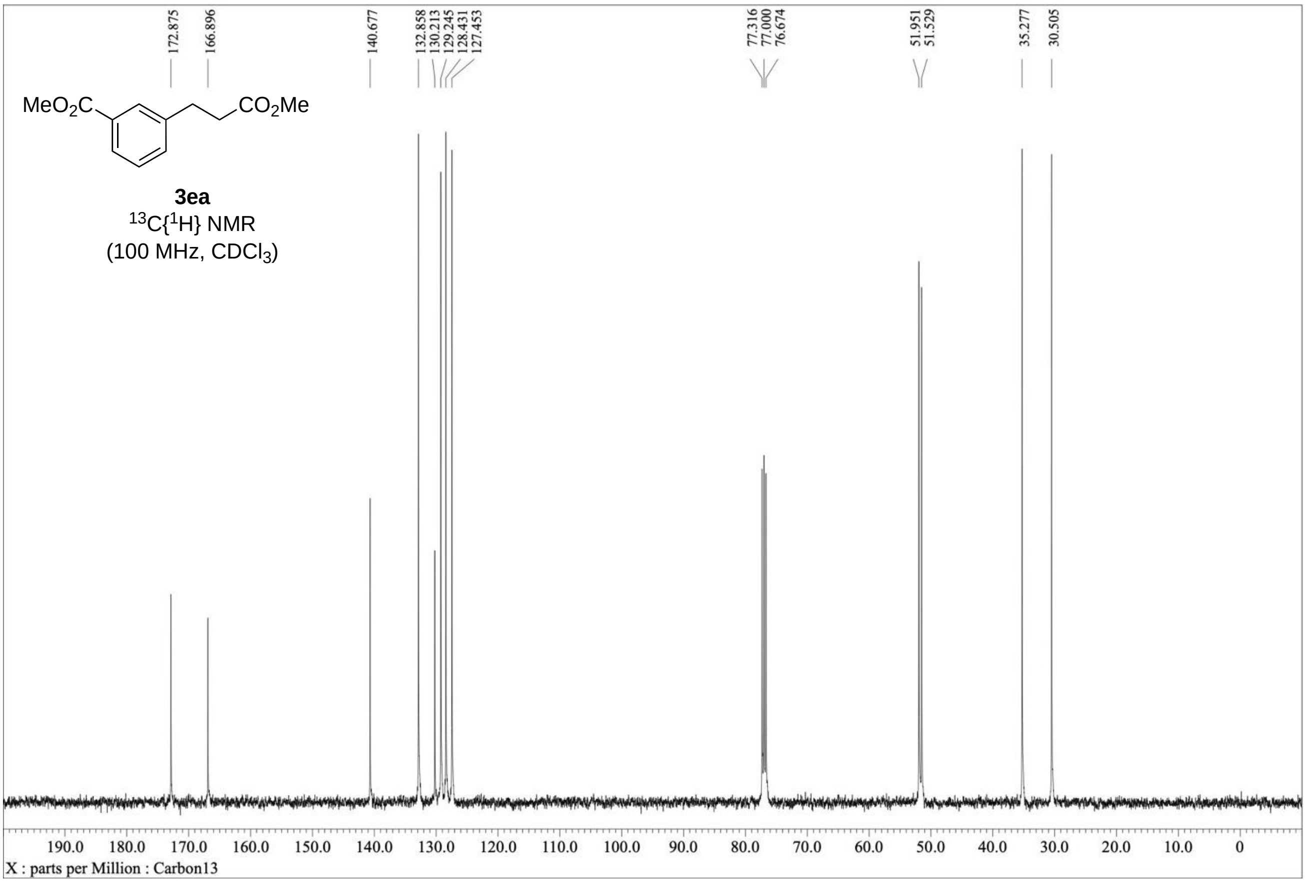
MeO2C
CO2Me
3ea
13C{1H} NMR
(100 MHz, CDCl3)
X : parts per Million : Carbon13

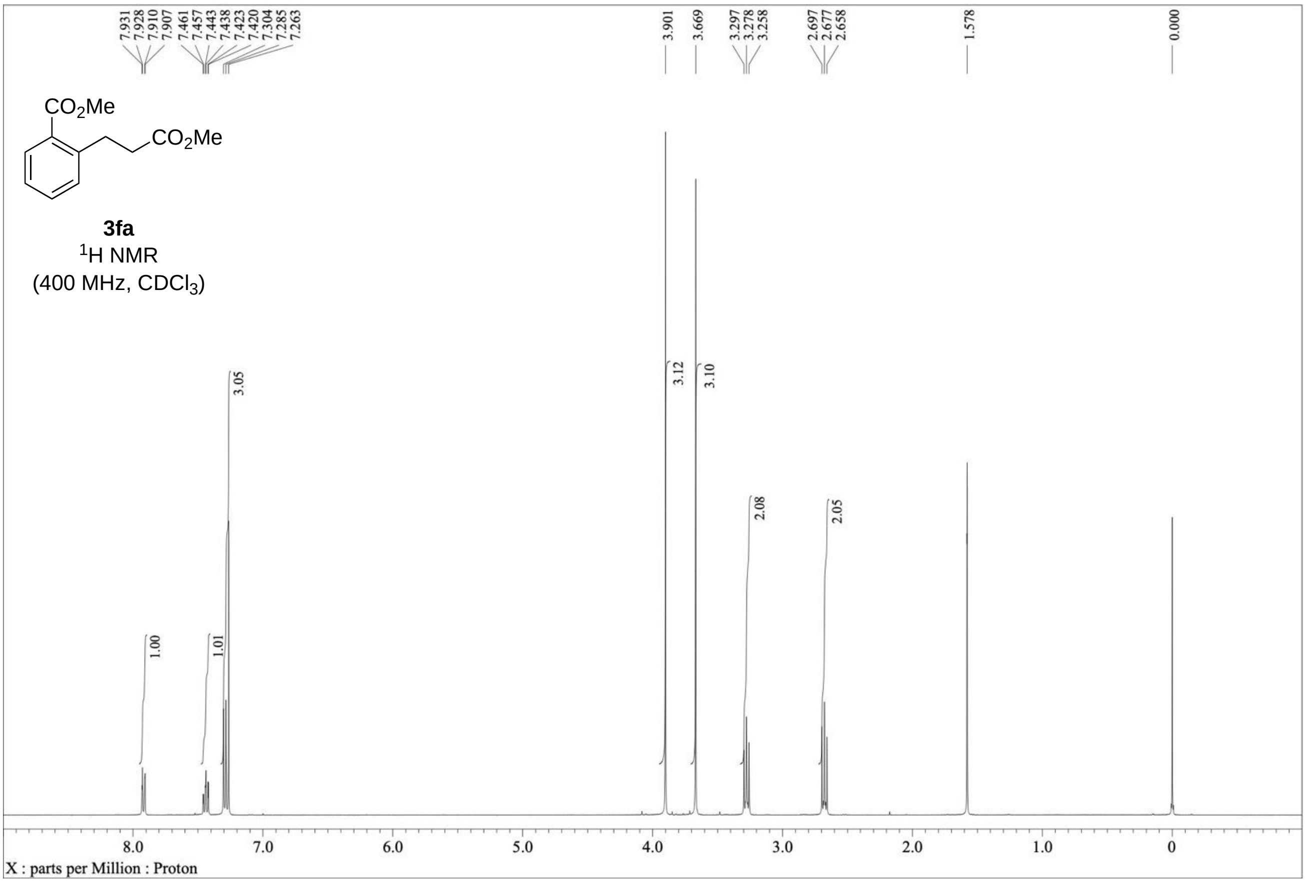

7.931
7.928
7.910
7.907
7.461
7.457
7.443
7.438
7.423
7.420
7.304
7.285
7.263
3.901
3.669
3.297
3.278
3.258
2.697
2.677
2.658
1.578
0.000
CO_2Me
CO_2Me
3fa
^1H NMR
(400 MHz, $CDCl_3$)
1.00
1.01
3.05
3.12
3.10
2.08
2.05
8.0
7.0
6.0
5.0
4.0
3.0
2.0
1.0
0
X : parts per Million : Proton

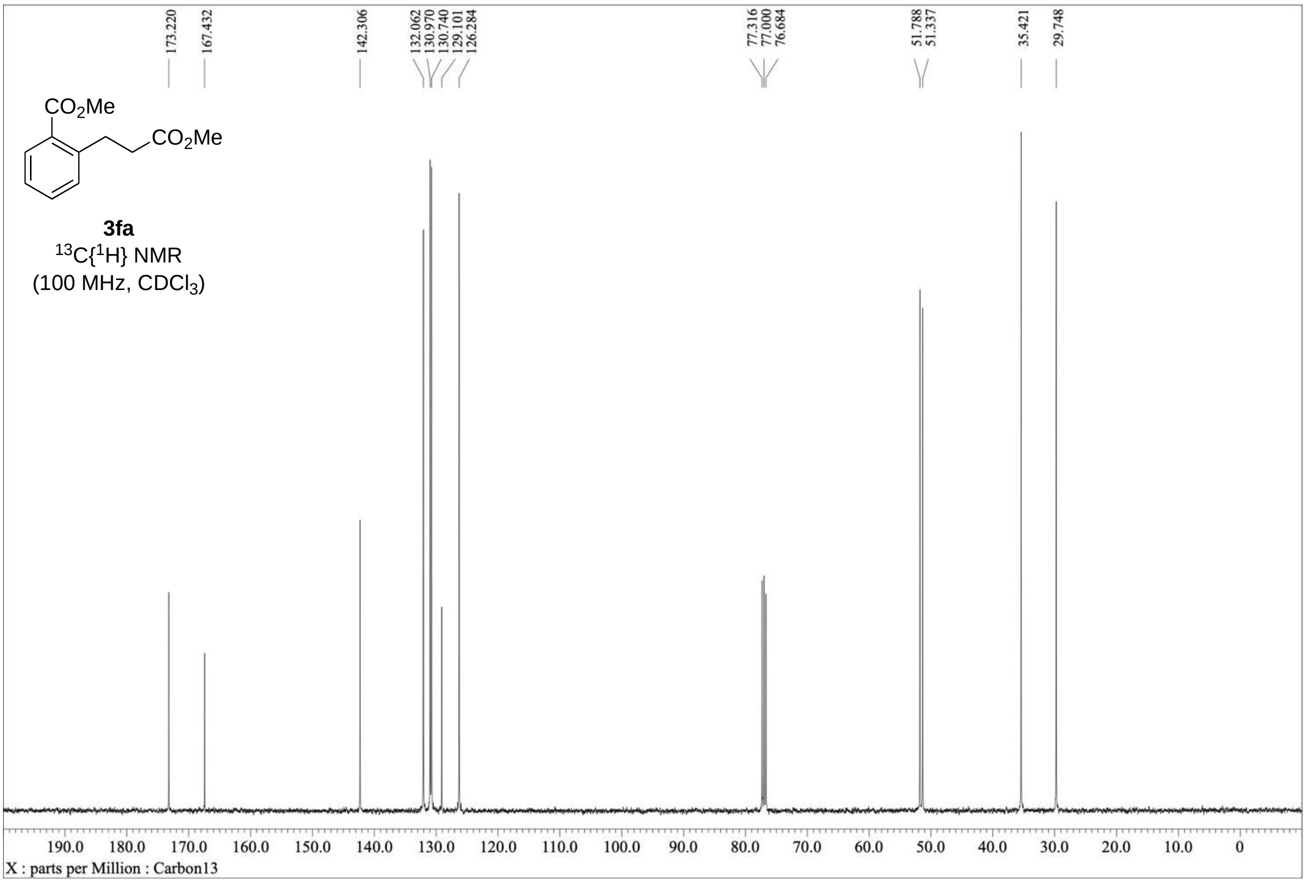

173.220
167.432
142.306
132.062
130.970
130.740
129.101
126.284
77.316
77.000
76.684
51.788
51.337
35.421
29.748
CO_2Me
CO_2Me
3fa
$^{13}C\{^1H\}$ NMR
(100 MHz, $CDCl_3$)
190.0
180.0
170.0
160.0
150.0
140.0
130.0
120.0
110.0
100.0
90.0
80.0
70.0
60.0
50.0
40.0
30.0
20.0
10.0
0
X : parts per Million : Carbon13

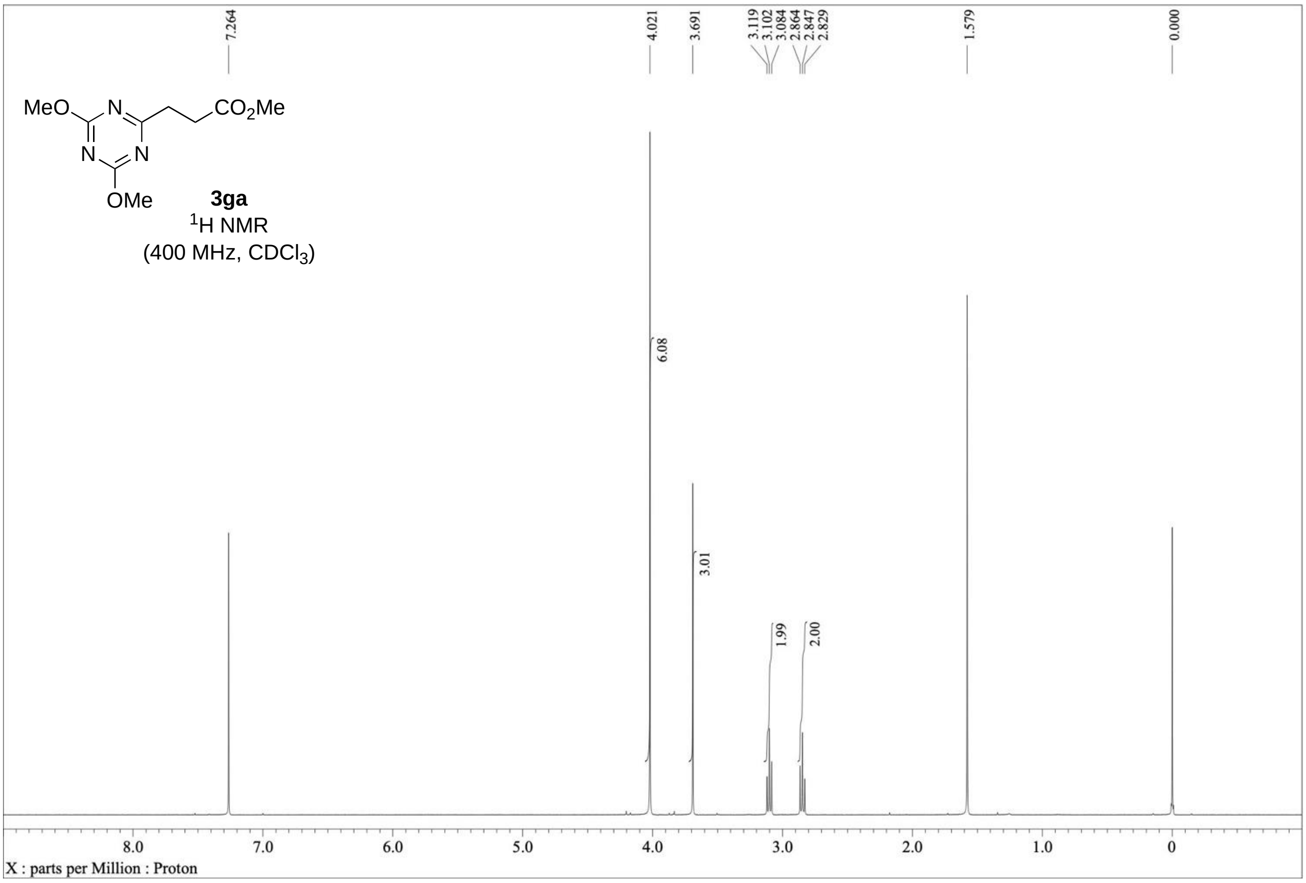
7.264
4.021
3.691
3.119
3.102
3.084
2.864
2.847
2.829
1.579
0.000
MeO
N
CO2Me
N
N
OMe
3ga
1H NMR
(400 MHz, CDCl3)
6.08
3.01
1.99
2.00
8.0
7.0
6.0
5.0
4.0
3.0
2.0
1.0
0
X : parts per Million : Proton

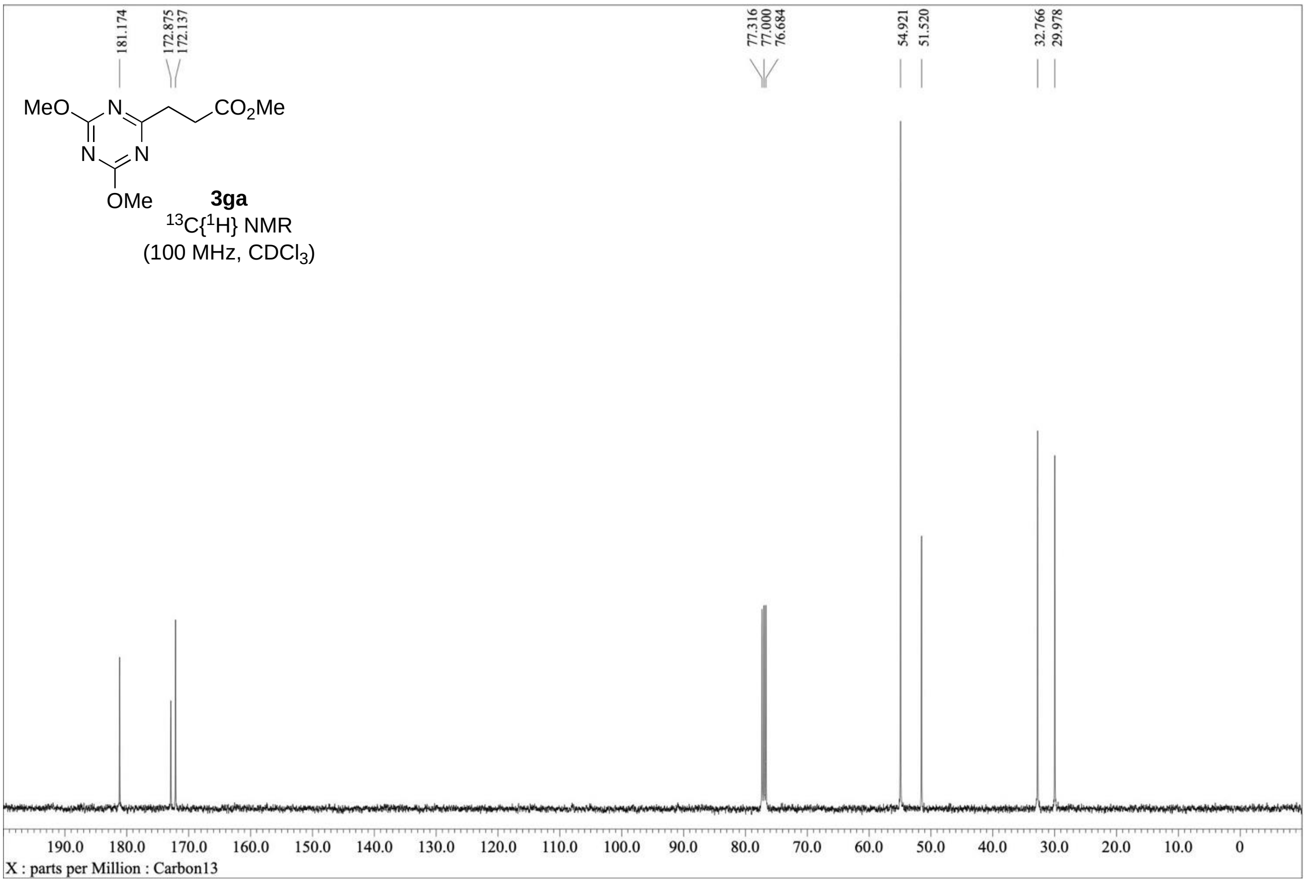
181.174
172.875
172.137
77.316
77.000
76.684
54.921
51.520
32.766
29.978
MeO
N
CO2Me
N
N
OMe
3ga
13C{1H} NMR
(100 MHz, CDCl3)
190.0
180.0
170.0
160.0
150.0
140.0
130.0
120.0
110.0
100.0
90.0
80.0
70.0
60.0
50.0
40.0
30.0
20.0
10.0
0
X : parts per Million : Carbon13

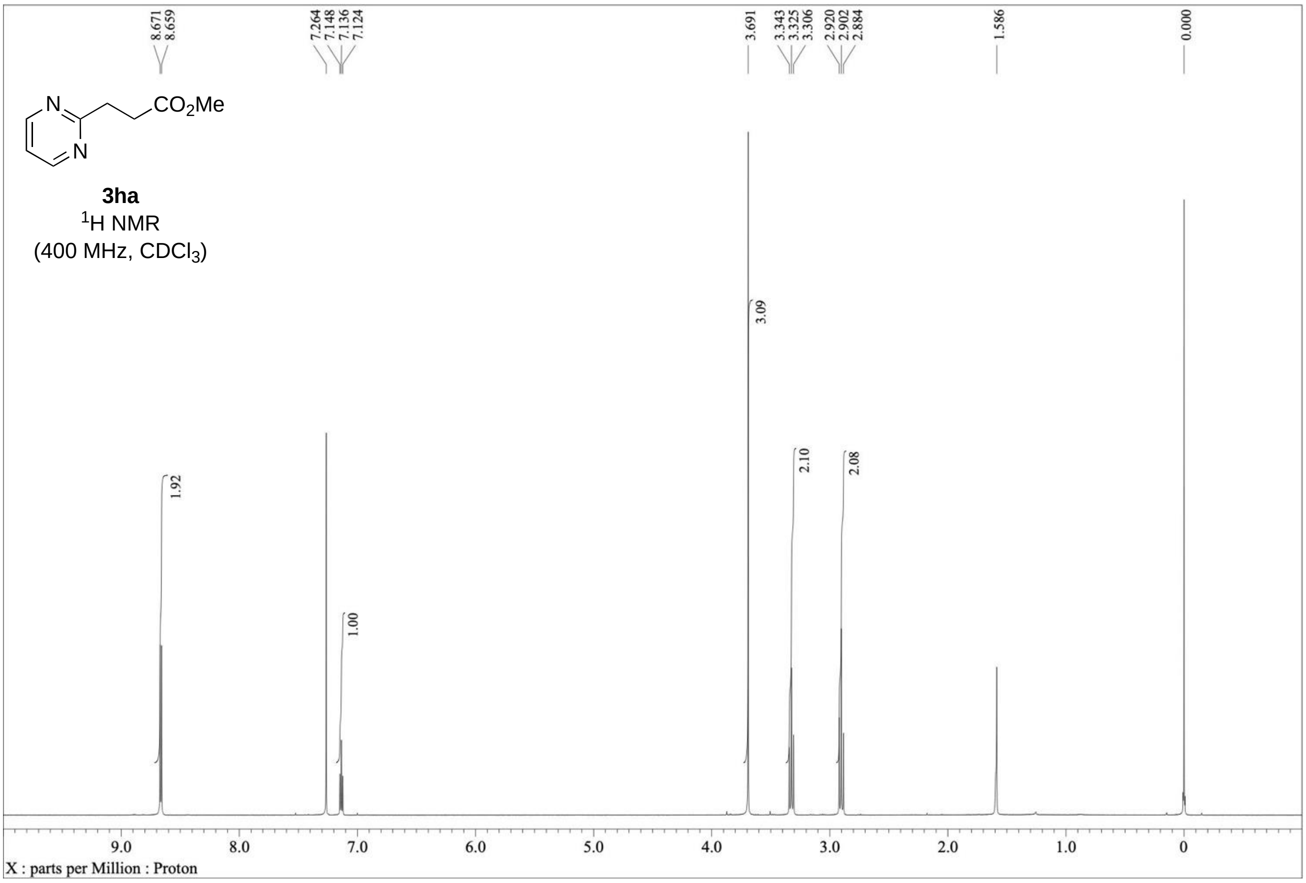

8.671
8.659
7.264
7.148
7.136
7.124
3.691
3.343
3.325
3.306
2.920
2.902
2.884
1.586
0.000
CO2Me
N
N
3ha
1H NMR
(400 MHz, CDCl3)
3.09
1.92
2.10
2.08
1.00
X : parts per Million : Proton

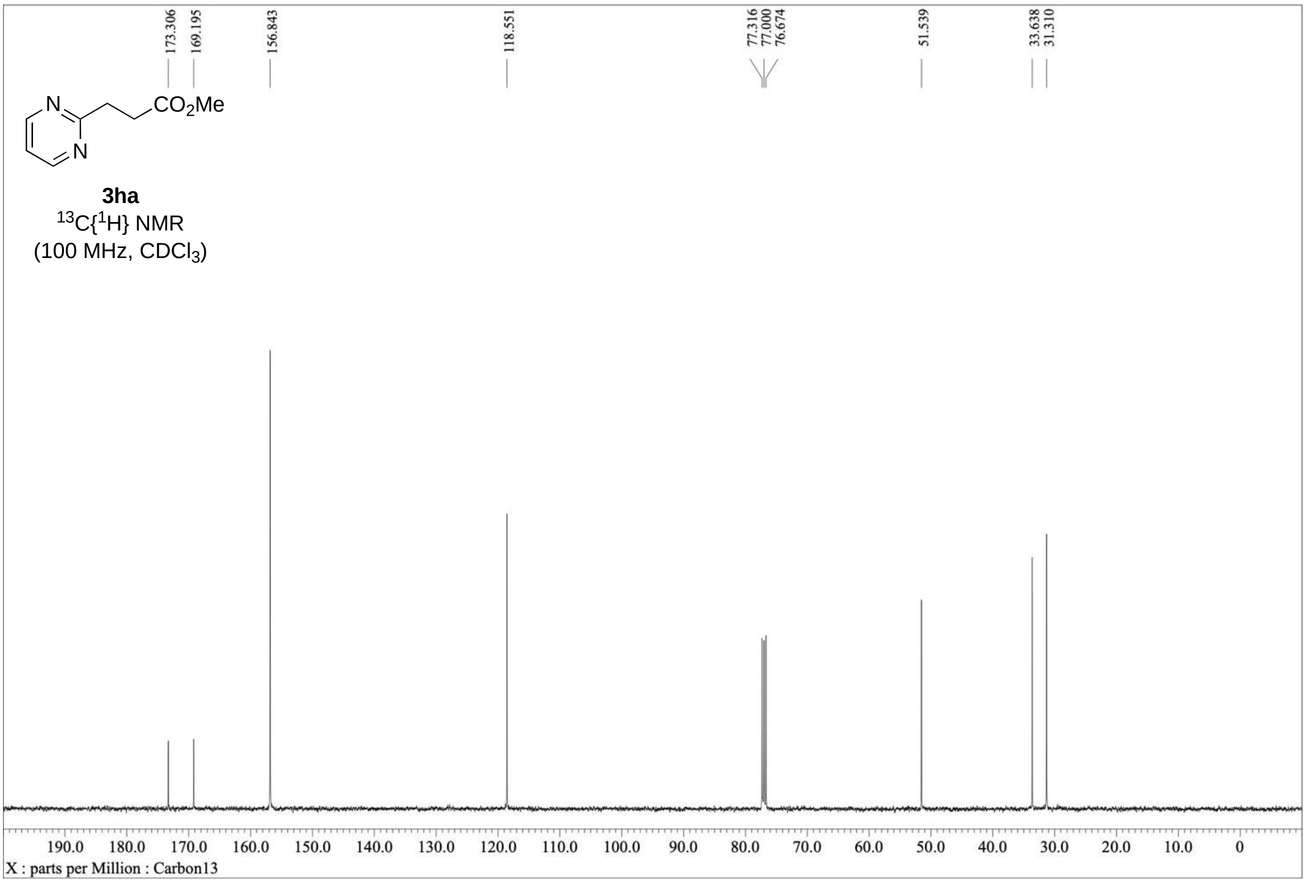

173.306
169.195
156.843
118.551
77.316
77.000
76.674
51.539
33.638
31.310
CO2Me
N
N
3ha
13C{1H} NMR
(100 MHz, CDCl3)
X : parts per Million : Carbon13

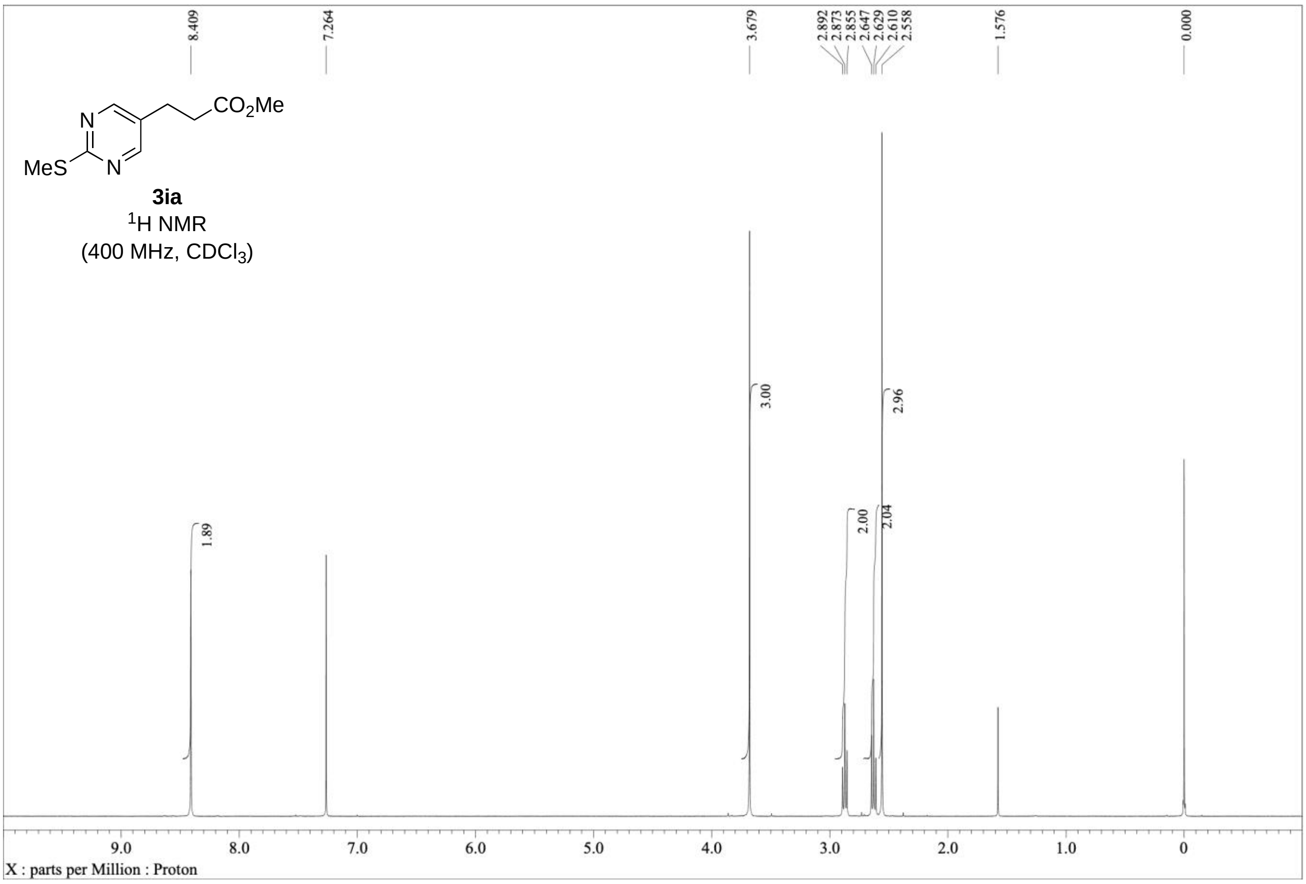

CO2Me
N
N
MeS
3ia
1H NMR
(400 MHz, CDCl3)
8.409
7.264
3.679
2.892
2.873
2.855
2.647
2.629
2.610
2.558
1.576
0.000
1.89
3.00
2.00
2.04
2.96
9.0
8.0
7.0
6.0
5.0
4.0
3.0
2.0
1.0
0
X : parts per Million : Proton

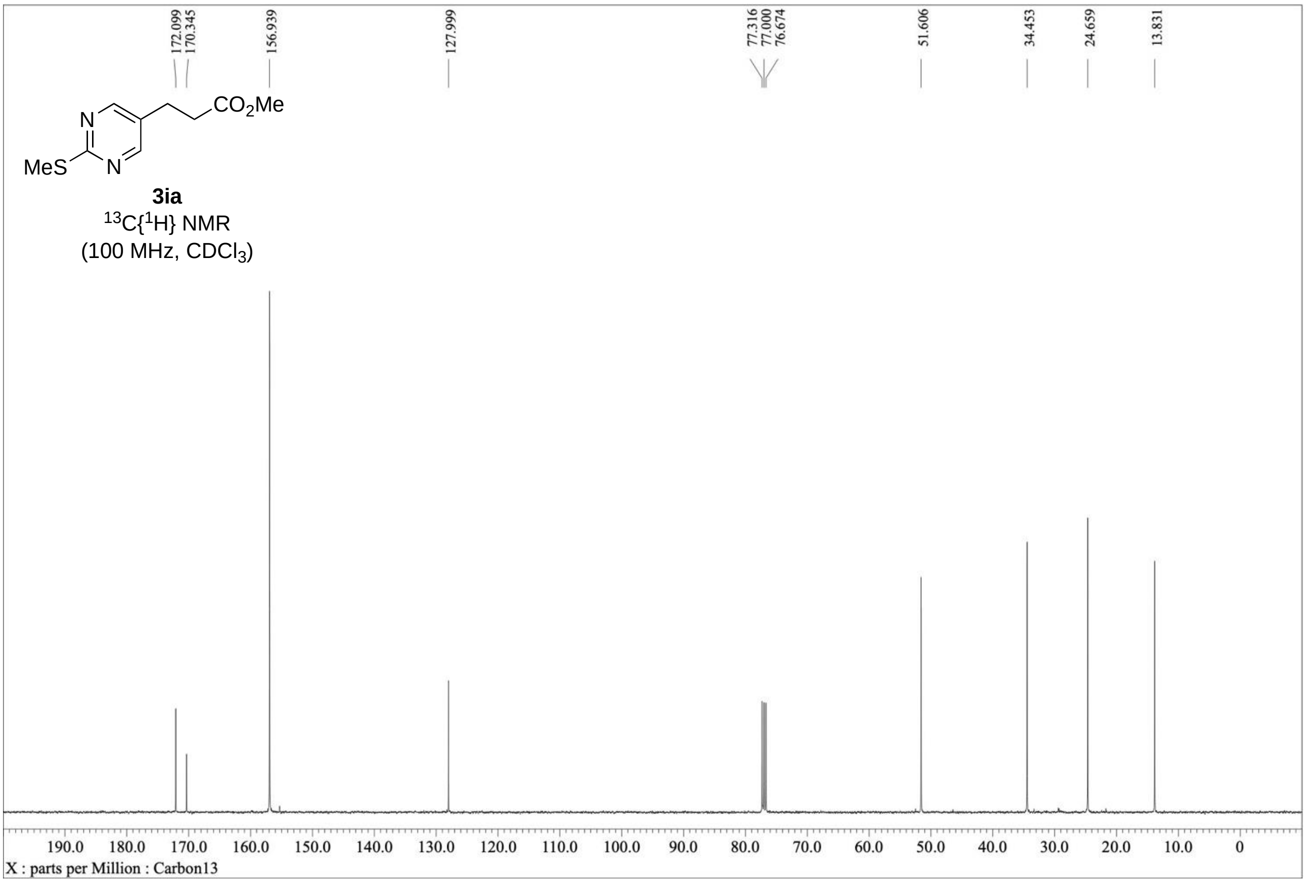

CO2Me
N
N
MeS
3ia
13C{1H} NMR
(100 MHz, CDCl3)
172.099
170.345
156.939
127.999
77.316
77.000
76.674
51.606
34.453
24.659
13.831
190.0
180.0
170.0
160.0
150.0
140.0
130.0
120.0
110.0
100.0
90.0
80.0
70.0
60.0
50.0
40.0
30.0
20.0
10.0
0
X : parts per Million : Carbon13

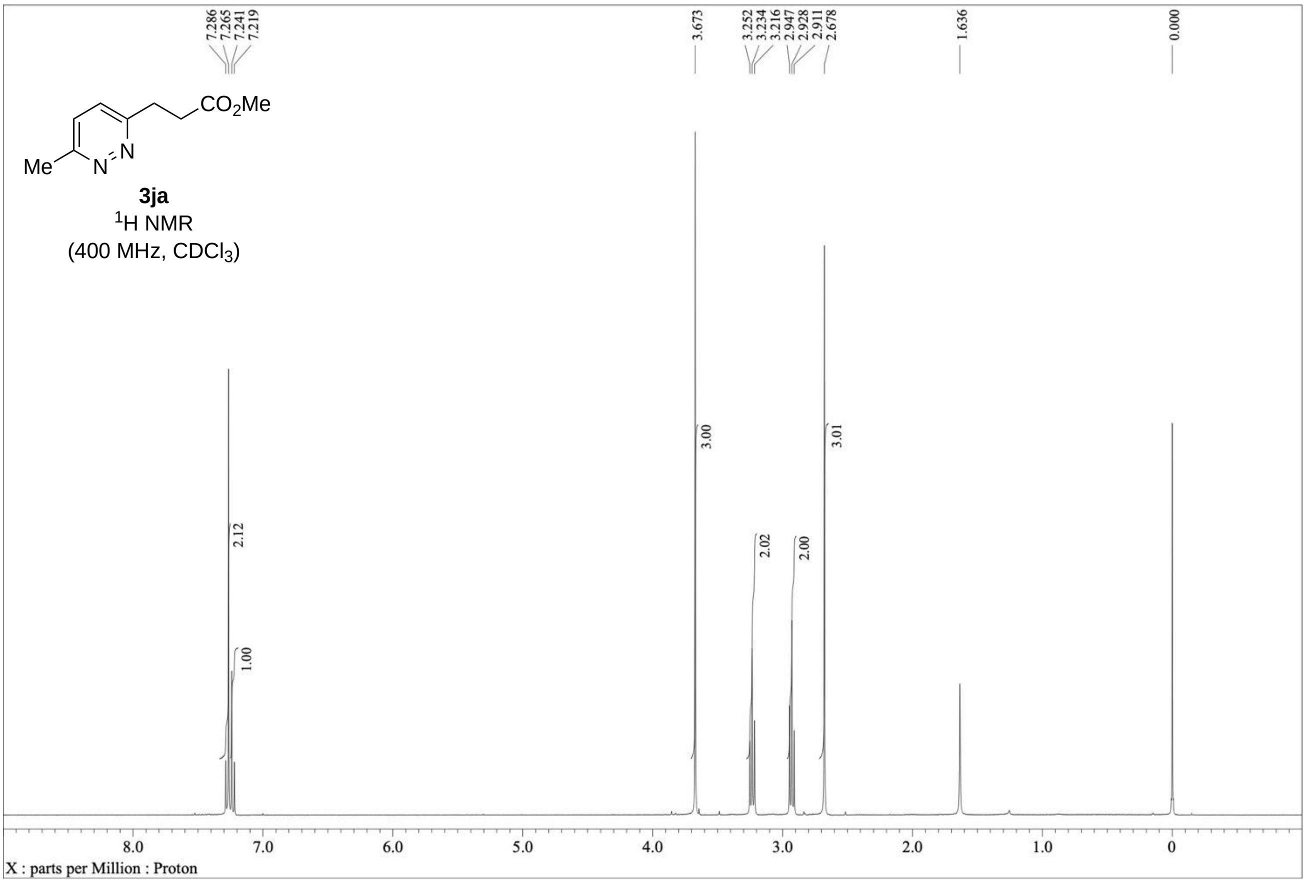
7.286
7.265
7.241
7.219
3.673
3.252
3.234
3.216
2.947
2.928
2.911
2.678
1.636
0.000
CO2Me
Me
N
N
3ja
1H NMR
(400 MHz, CDCl3)
2.12
1.00
3.00
2.02
2.00
3.01
8.0
7.0
6.0
5.0
4.0
3.0
2.0
1.0
0
X : parts per Million : Proton

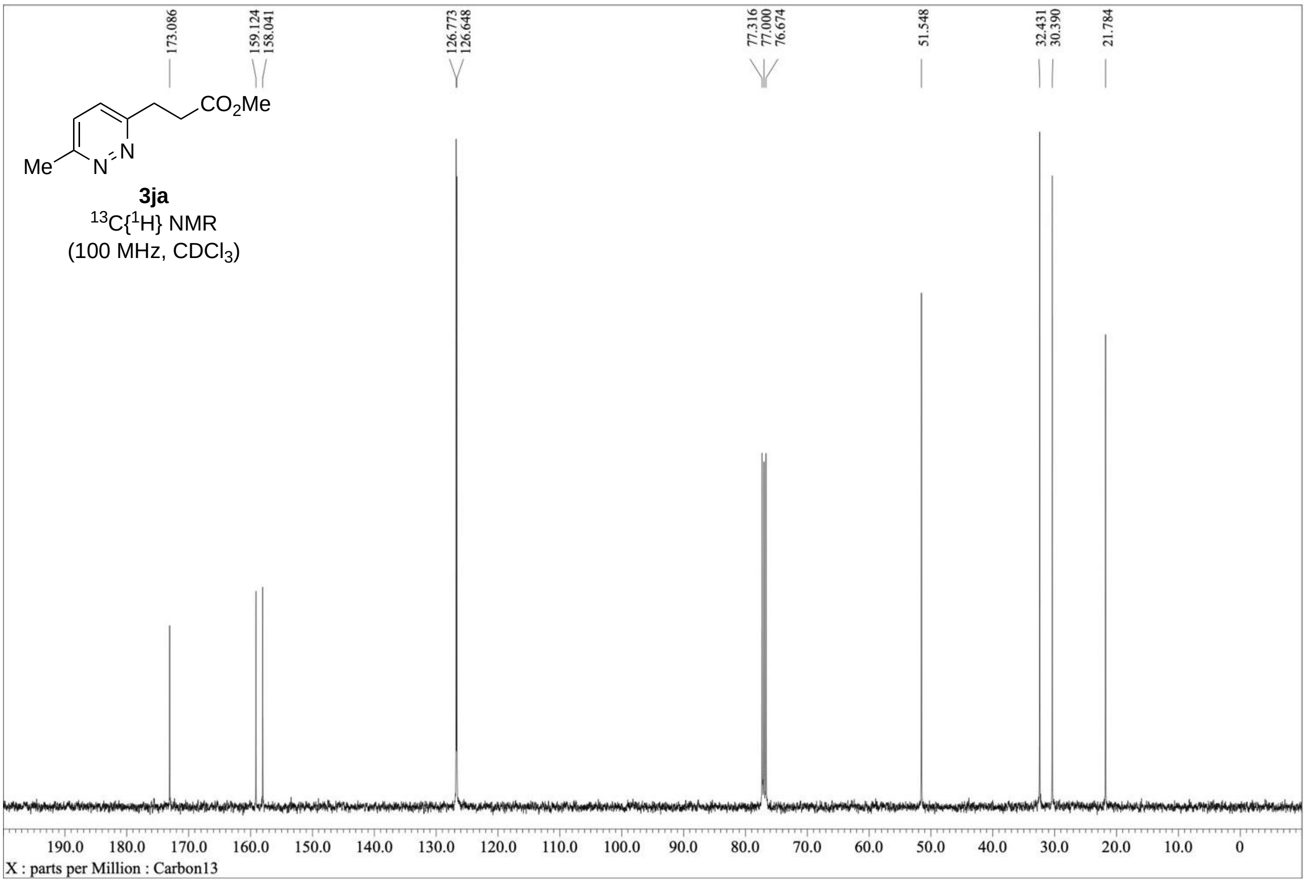
173.086
159.124
158.041
126.773
126.648
77.316
77.000
76.674
51.548
32.431
30.390
21.784
CO2Me
Me
N
N
3ja
13C{1H} NMR
(100 MHz, CDCl3)
190.0
180.0
170.0
160.0
150.0
140.0
130.0
120.0
110.0
100.0
90.0
80.0
70.0
60.0
50.0
40.0
30.0
20.0
10.0
0
X : parts per Million : Carbon13

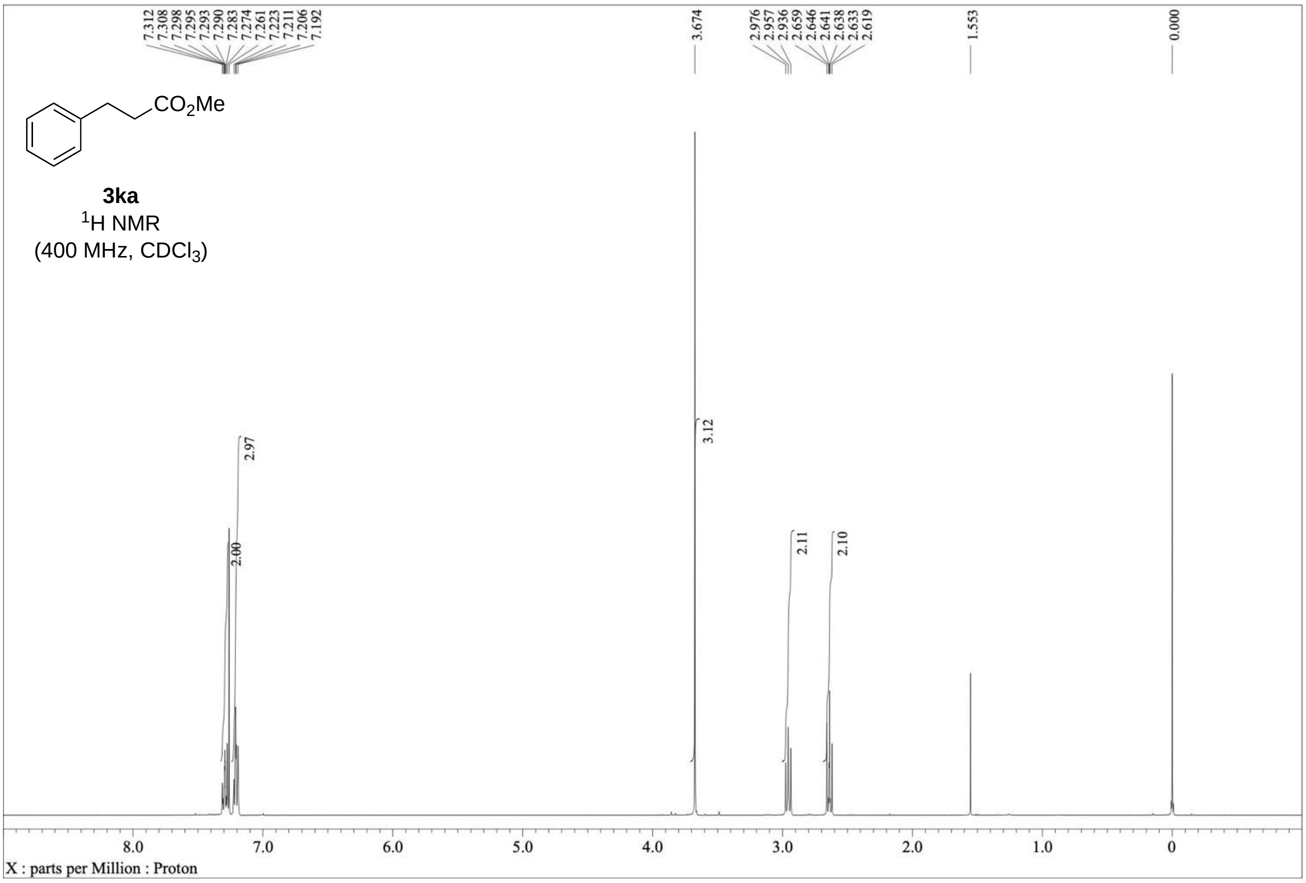
7.312
7.308
7.298
7.295
7.293
7.290
7.283
7.274
7.261
7.223
7.211
7.206
7.192
3.674
2.976
2.957
2.936
2.659
2.646
2.641
2.638
2.633
2.619
1.553
0.000
CO2Me
3ka
1H NMR
(400 MHz, CDCl3)
2.97
2.00
3.12
2.11
2.10
8.0
7.0
6.0
5.0
4.0
3.0
2.0
1.0
0
X : parts per Million : Proton

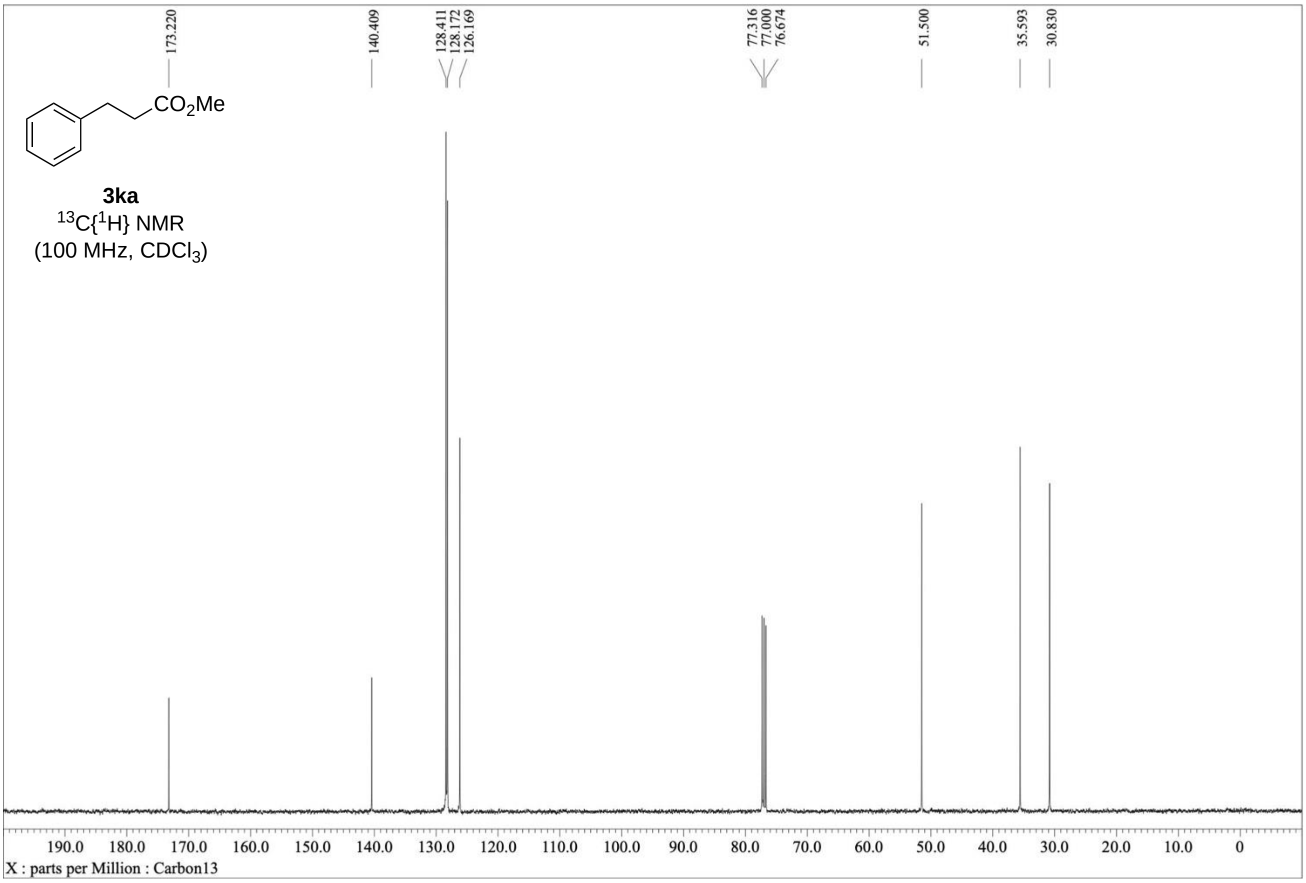
173.220
140.409
128.411
128.172
126.169
77.316
77.000
76.674
51.500
35.593
30.830
CO2Me
3ka
13C{1H} NMR
(100 MHz, CDCl3)
190.0
180.0
170.0
160.0
150.0
140.0
130.0
120.0
110.0
100.0
90.0
80.0
70.0
60.0
50.0
40.0
30.0
20.0
10.0
0
X : parts per Million : Carbon13

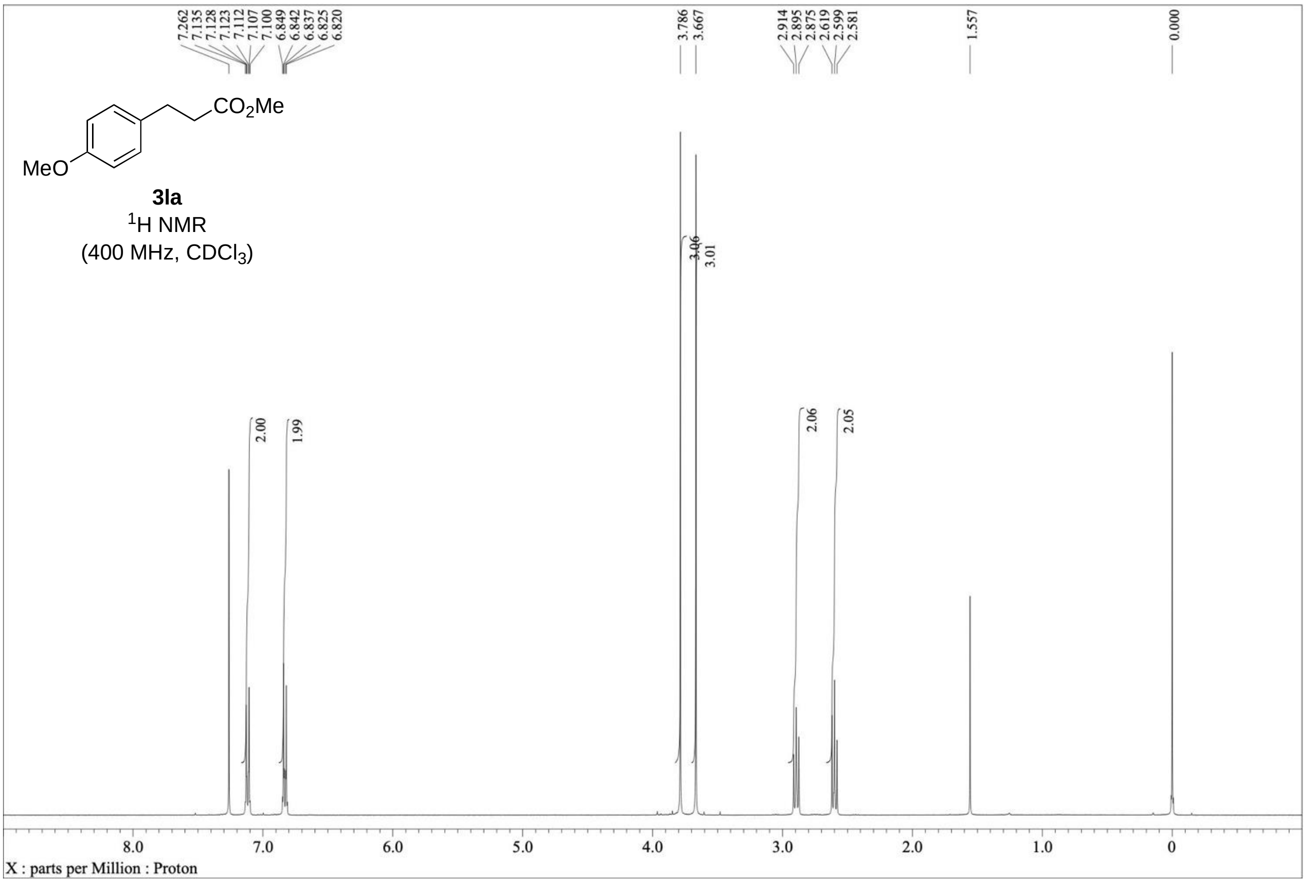
7.262
7.135
7.128
7.123
7.112
7.107
7.100
6.849
6.842
6.837
6.825
6.820
3.786
3.667
2.914
2.895
2.875
2.619
2.599
2.581
1.557
0.000
CO2Me
MeO
3la
1H NMR
(400 MHz, CDCl3)
2.00
1.99
3.06
3.01
2.06
2.05
8.0
7.0
6.0
5.0
4.0
3.0
2.0
1.0
0
X : parts per Million : Proton

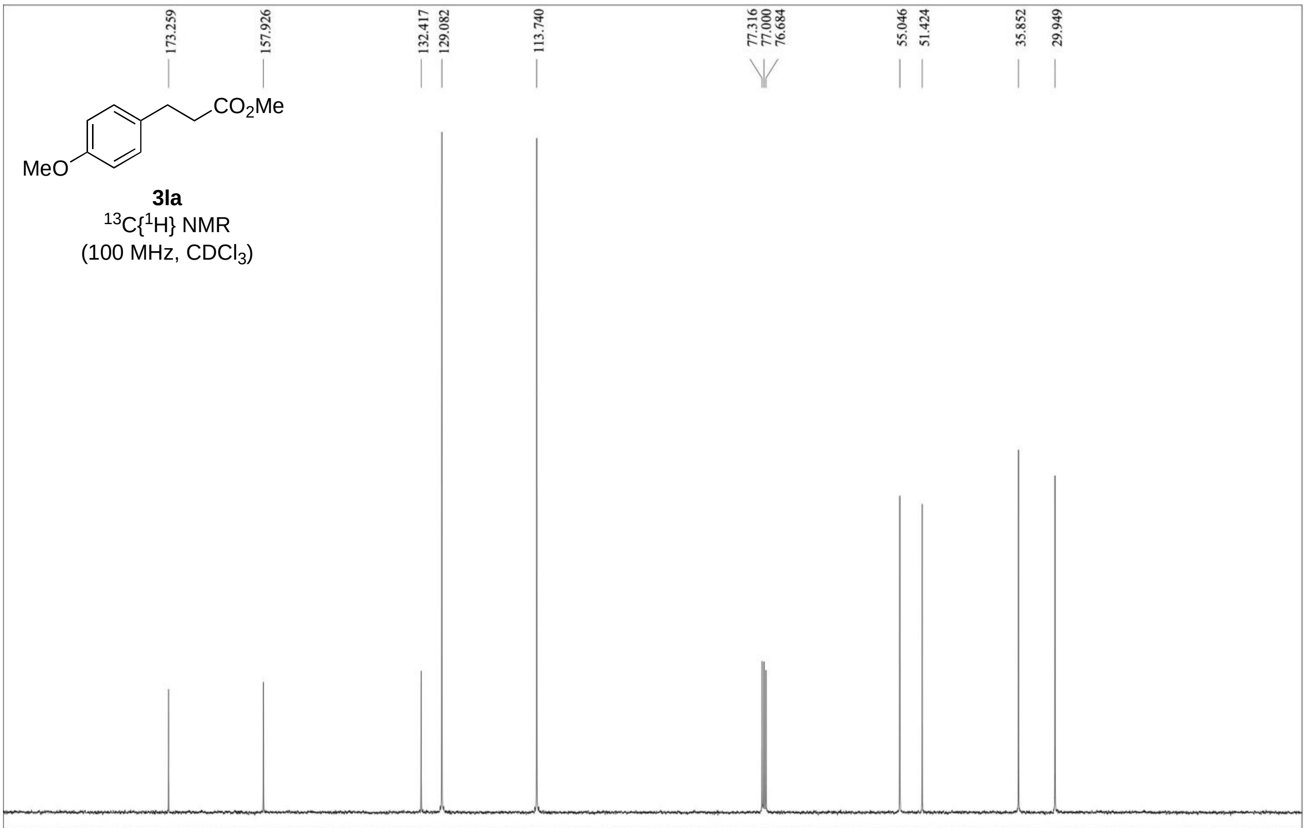
173.259
157.926
132.417
129.082
113.740
77.316
77.000
76.684
55.046
51.424
35.852
29.949
CO2Me
MeO
3la
13C{1H} NMR
(100 MHz, CDCl3)
190.0
180.0
170.0
160.0
150.0
140.0
130.0
120.0
110.0
100.0
90.0
80.0
70.0
60.0
50.0
40.0
30.0
20.0
10.0
0
X : parts per Million : Carbon13

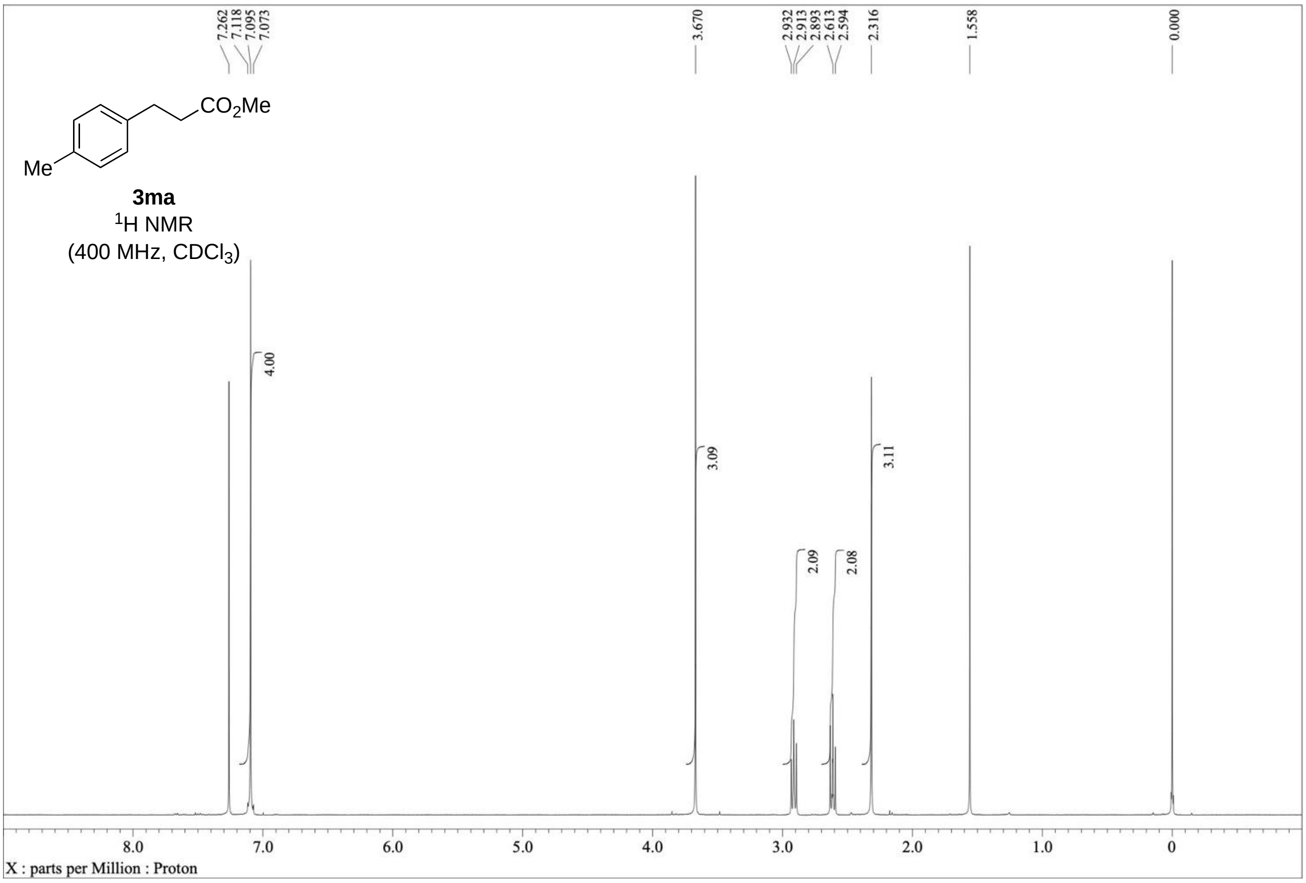
7.262
7.118
7.095
7.073
3.670
2.932
2.913
2.893
2.613
2.594
2.316
1.558
0.000
CO2Me
Me
3ma
1H NMR
(400 MHz, CDCl3)
4.00
3.09
2.09
2.08
3.11
8.0
7.0
6.0
5.0
4.0
3.0
2.0
1.0
0
X : parts per Million : Proton

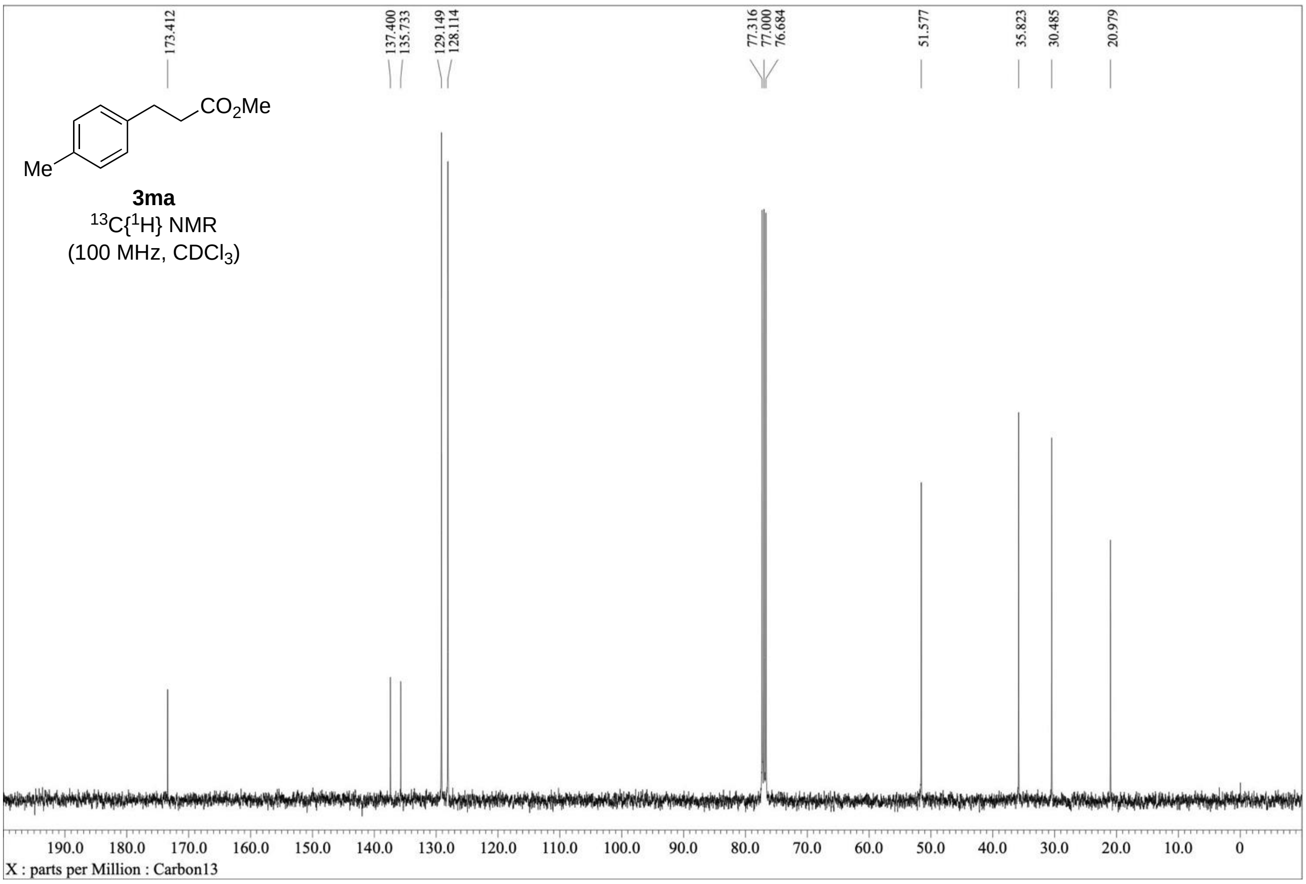
173.412
137.400
135.733
129.149
128.114
77.316
77.000
76.684
51.577
35.823
30.485
20.979
CO2Me
Me
3ma
13C{1H} NMR
(100 MHz, CDCl3)
190.0
180.0
170.0
160.0
150.0
140.0
130.0
120.0
110.0
100.0
90.0
80.0
70.0
60.0
50.0
40.0
30.0
20.0
10.0
0
X : parts per Million : Carbon13

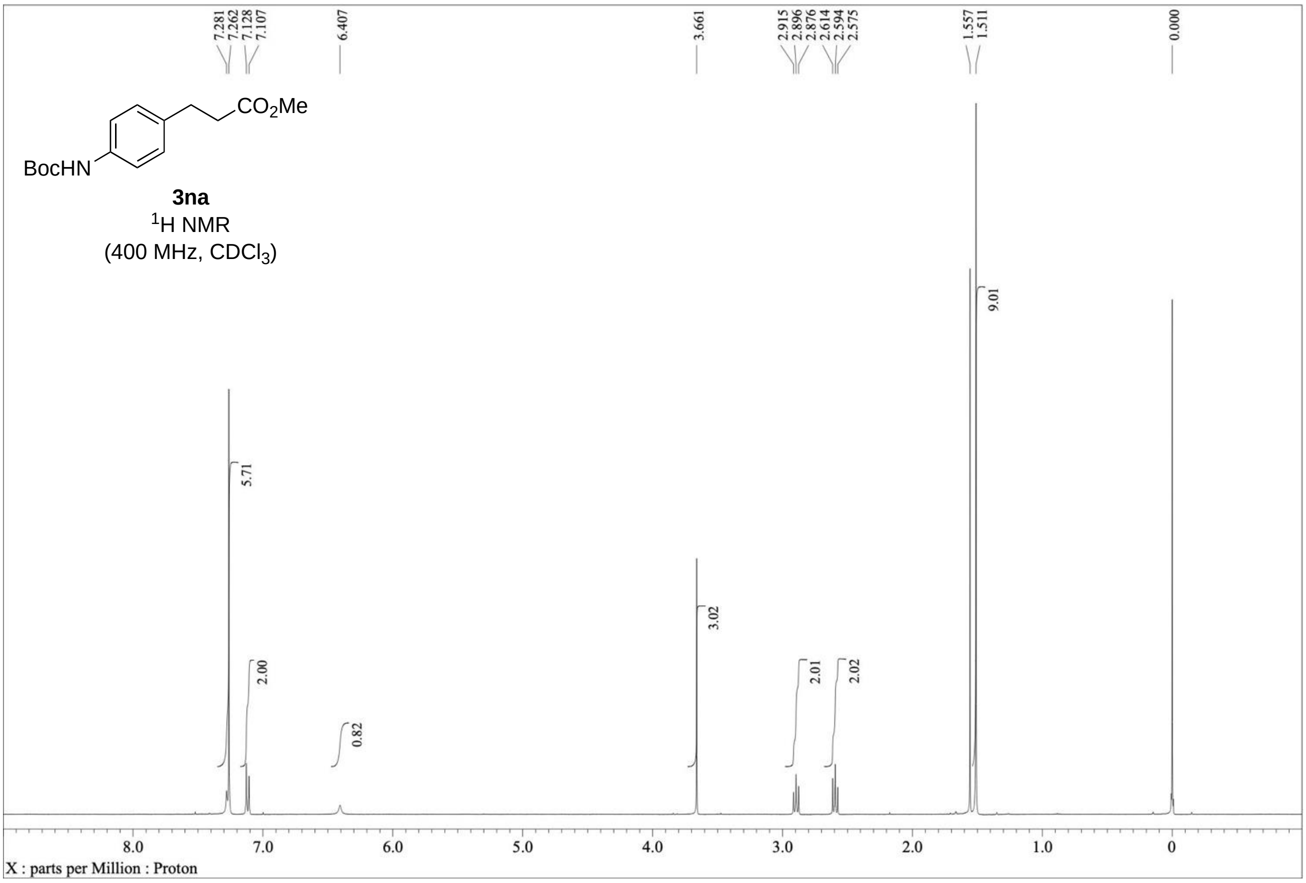
7.281
7.262
7.128
7.107
6.407
3.661
2.915
2.896
2.876
2.614
2.594
2.575
1.557
1.511
0.000
CO2Me
BocHN
3na
1H NMR
(400 MHz, CDCl3)
9.01
5.71
3.02
2.00
0.82
2.01
2.02
8.0 7.0 6.0 5.0 4.0 3.0 2.0 1.0 0
X : parts per Million : Proton

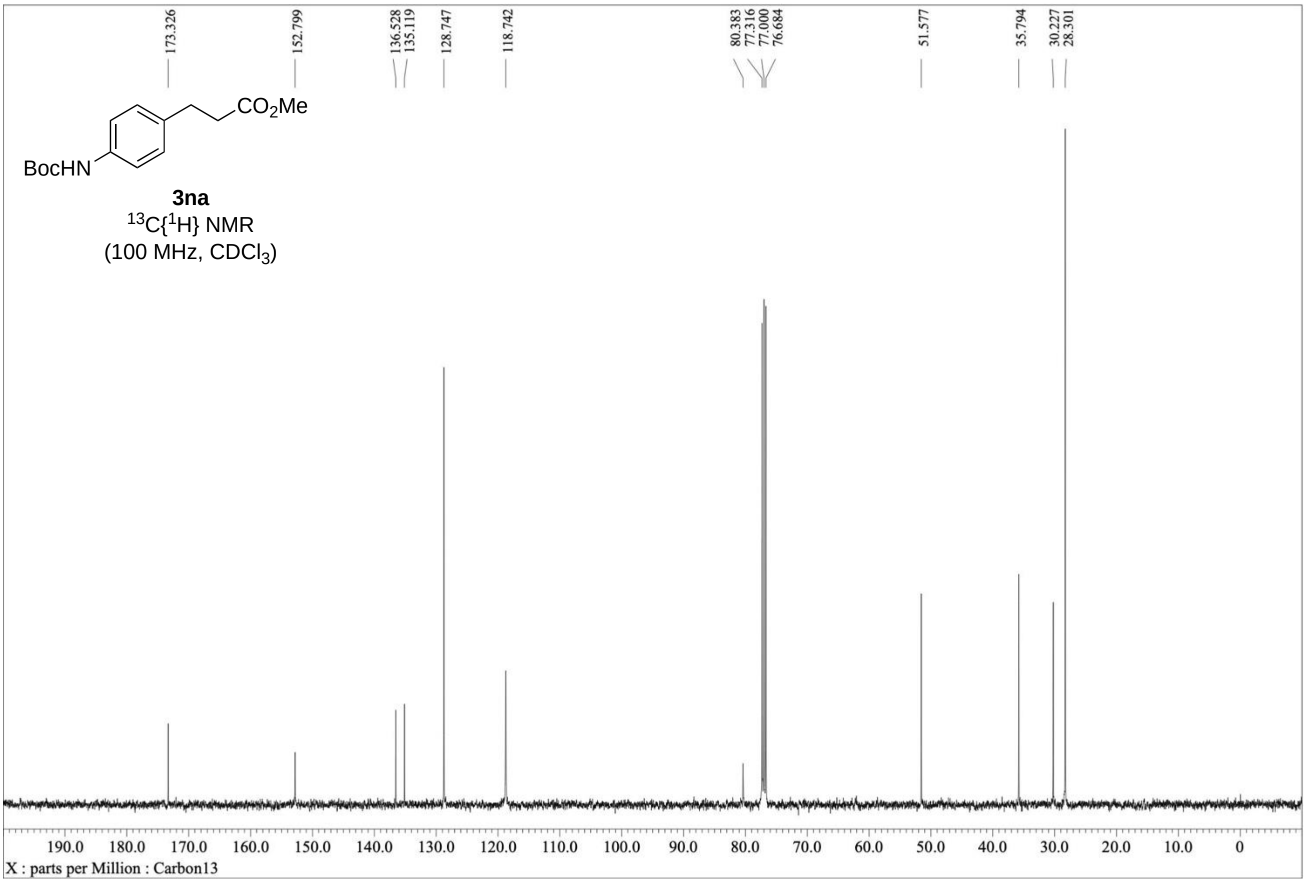
173.326
152.799
136.528
135.119
128.747
118.742
80.383
77.316
77.000
76.684
51.577
35.794
30.227
28.301
CO2Me
BocHN
3na
13C{1H} NMR
(100 MHz, CDCl3)
190.0 180.0 170.0 160.0 150.0 140.0 130.0 120.0 110.0 100.0 90.0 80.0 70.0 60.0 50.0 40.0 30.0 20.0 10.0 0
X : parts per Million : Carbon13

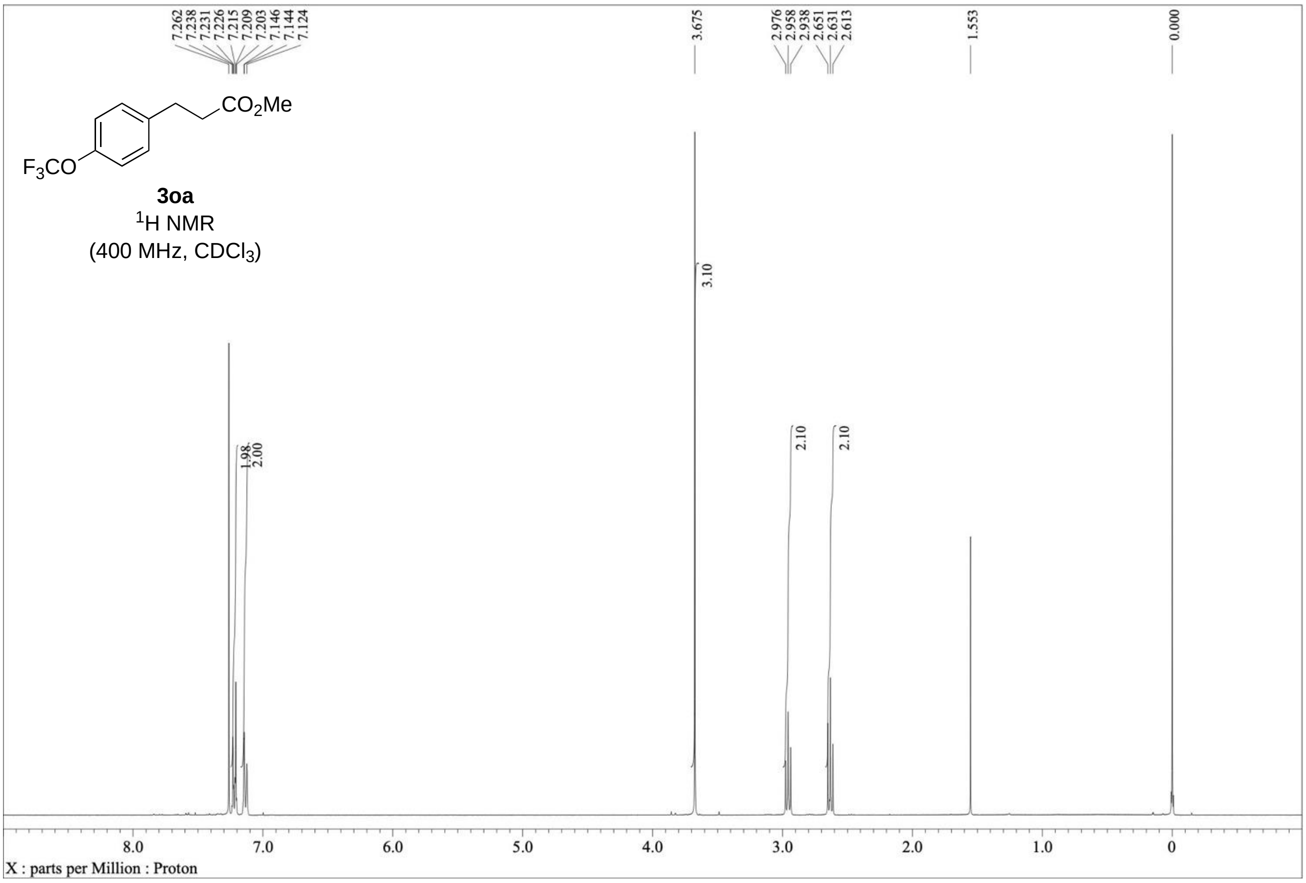
7.262
7.238
7.231
7.226
7.215
7.209
7.203
7.146
7.144
7.124
3.675
2.976
2.958
2.938
2.651
2.631
2.613
1.553
0.000
CO2Me
F3CO
3oa
1H NMR
(400 MHz, CDCl3)
3.10
1.98
2.00
2.10
2.10
X : parts per Million : Proton

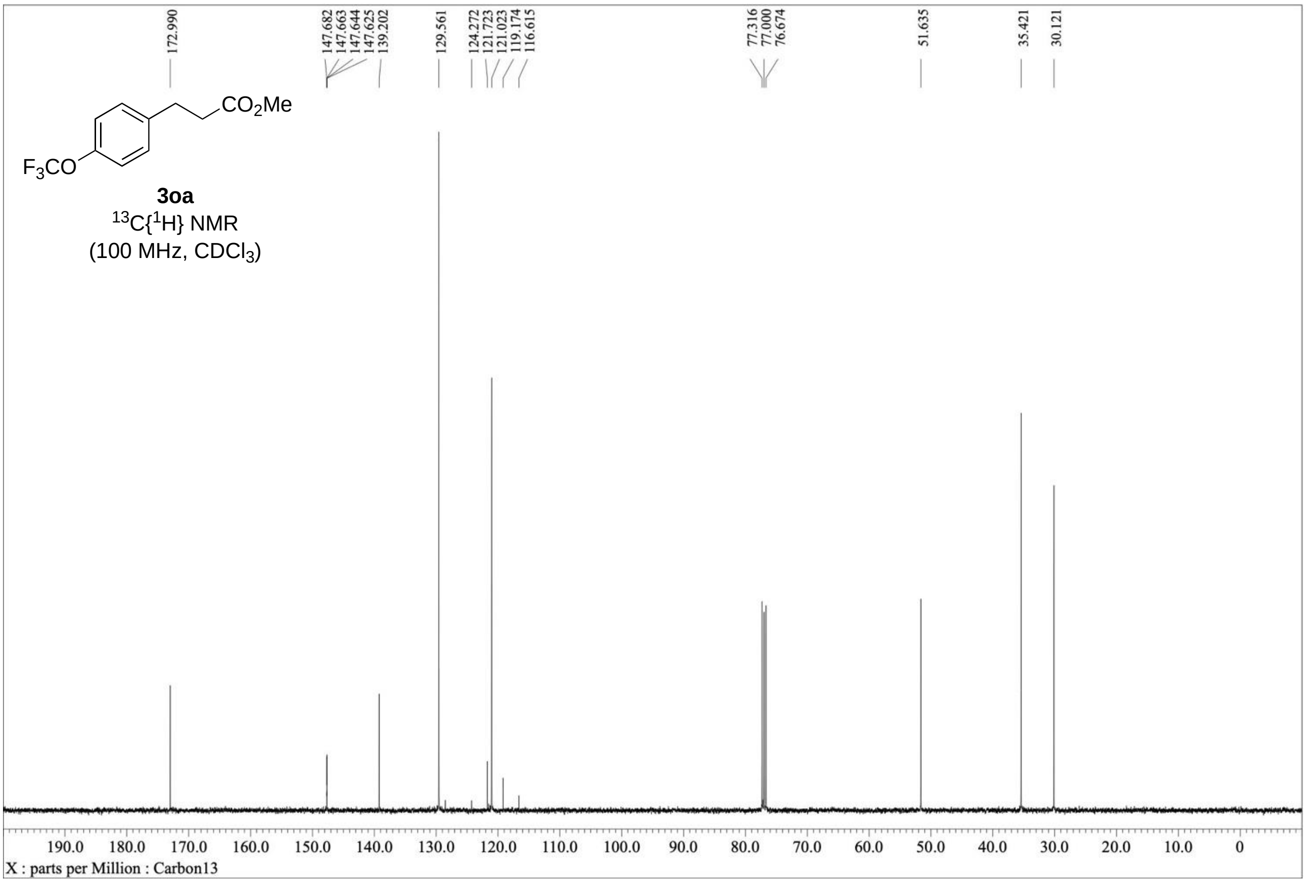
172.990
147.682
147.663
147.644
147.625
139.202
129.561
124.272
121.723
121.023
119.174
116.615
77.316
77.000
76.674
51.635
35.421
30.121
CO2Me
F3CO
3oa
13C{1H} NMR
(100 MHz, CDCl3)
X : parts per Million : Carbon13

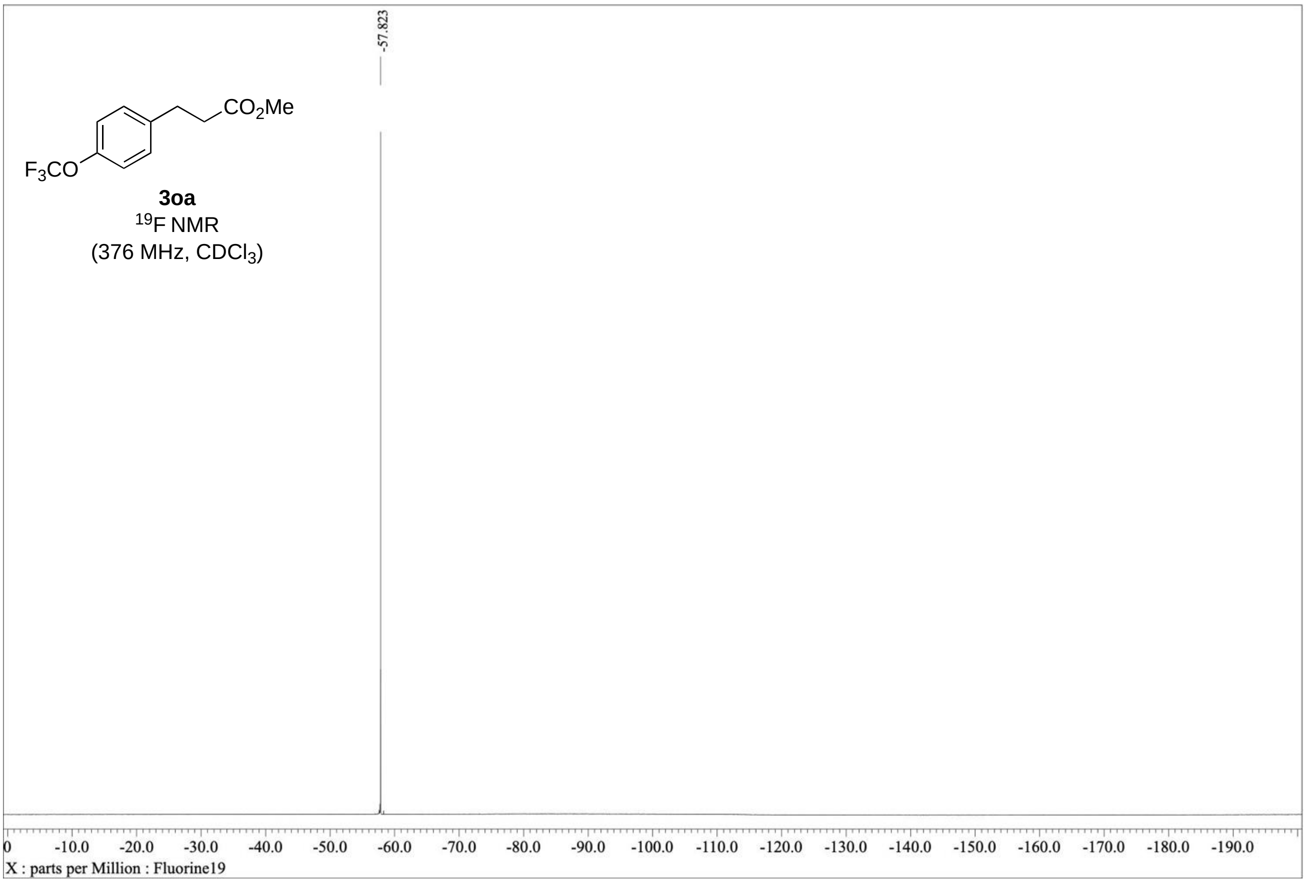
-57.823
CO2Me
F3CO
3oa
19F NMR
(376 MHz, CDCl3)
0 -10.0 -20.0 -30.0 -40.0 -50.0 -60.0 -70.0 -80.0 -90.0 -100.0 -110.0 -120.0 -130.0 -140.0 -150.0 -160.0 -170.0 -180.0 -190.0
X : parts per Million : Fluorine19

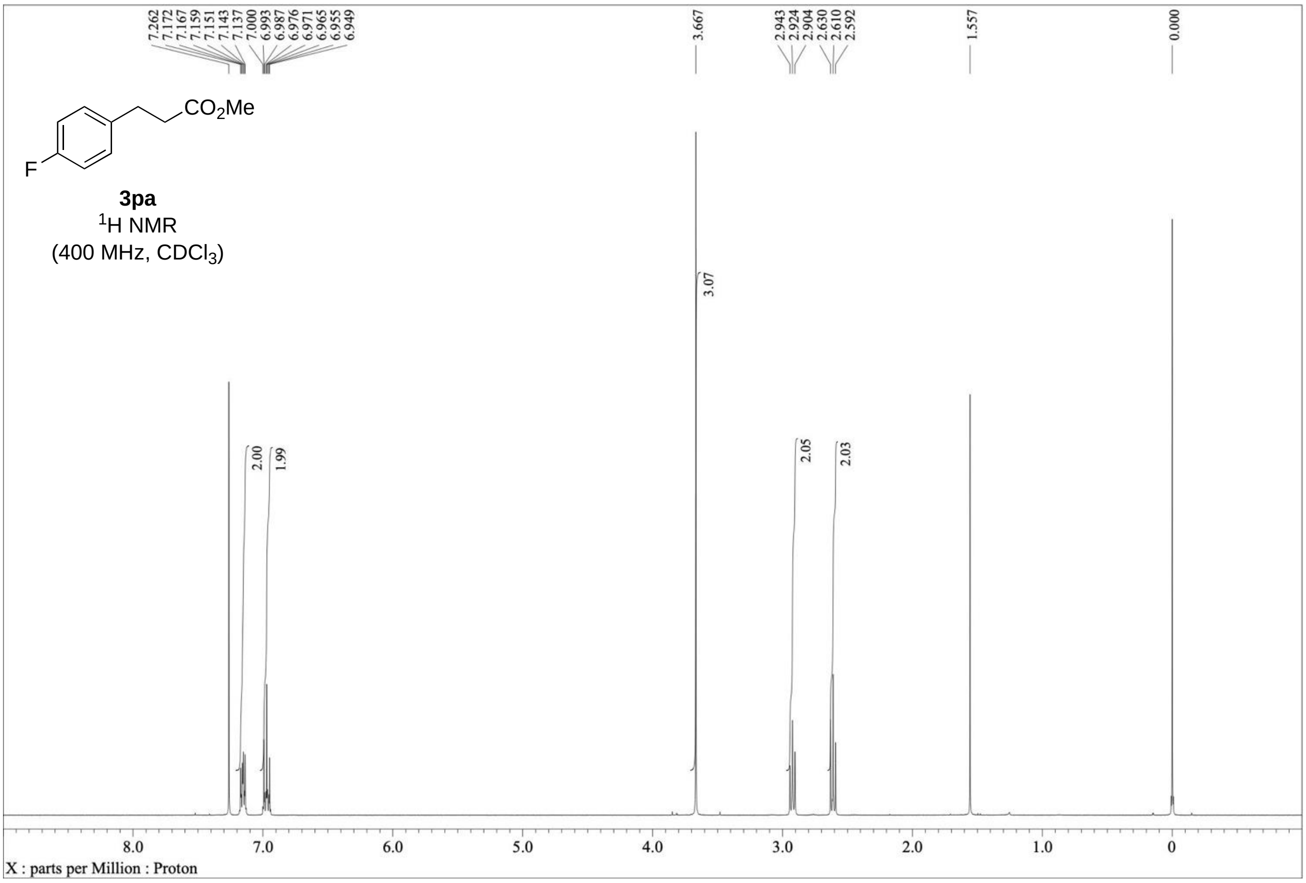

7.262
7.172
7.167
7.159
7.151
7.143
7.137
7.000
6.993
6.987
6.976
6.971
6.965
6.955
6.949
3.667
2.943
2.924
2.904
2.630
2.610
2.592
1.557
0.000
CO2Me
F
3pa
1H NMR
(400 MHz, CDCl3)
3.07
2.00
1.99
2.05
2.03
8.0
7.0
6.0
5.0
4.0
3.0
2.0
1.0
0
X : parts per Million : Proton

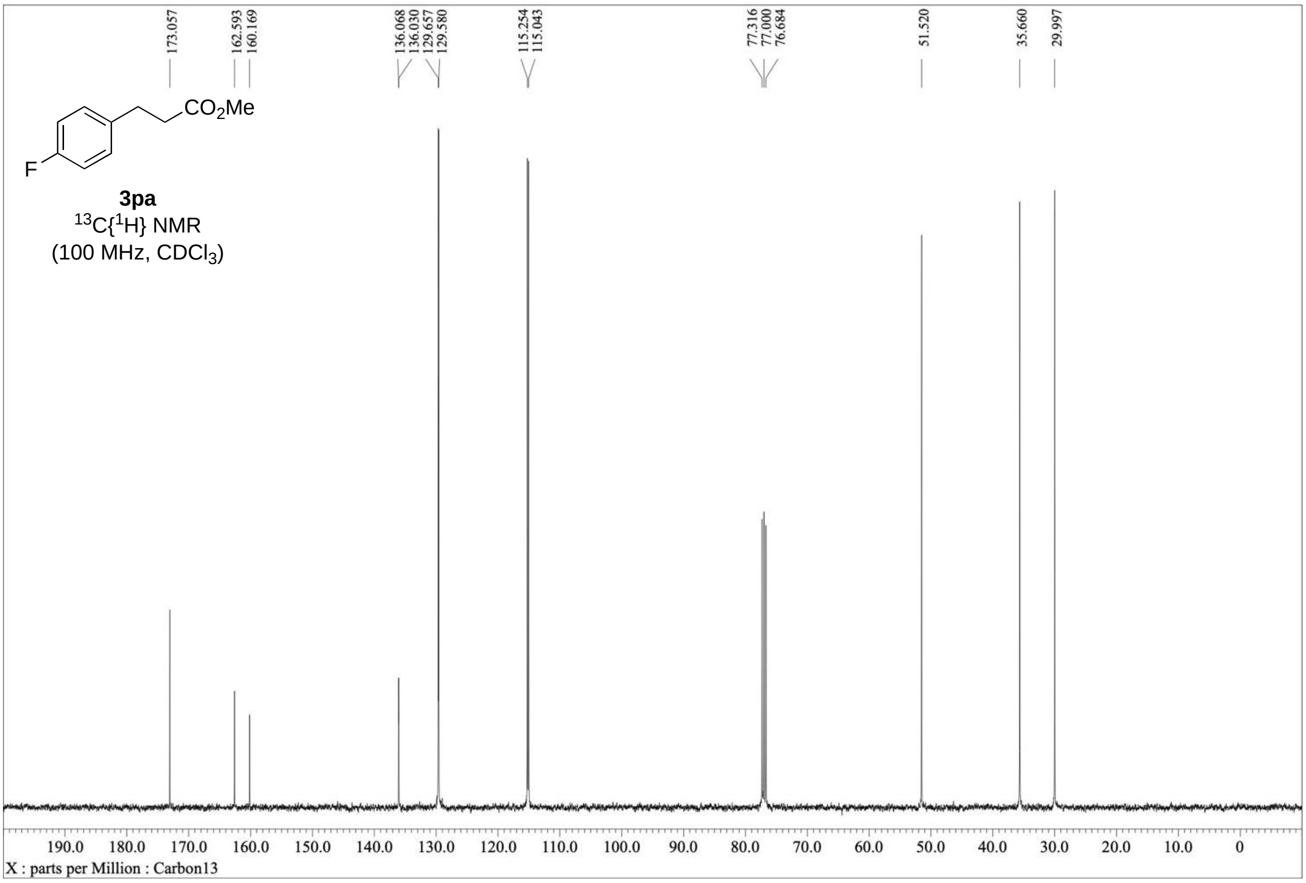

173.057
162.593
160.169
136.068
136.030
129.657
129.580
115.254
115.043
77.316
77.000
76.684
51.520
35.660
29.997
CO2Me
F
3pa
13C{1H} NMR
(100 MHz, CDCl3)
190.0
180.0
170.0
160.0
150.0
140.0
130.0
120.0
110.0
100.0
90.0
80.0
70.0
60.0
50.0
40.0
30.0
20.0
10.0
0
X : parts per Million : Carbon13

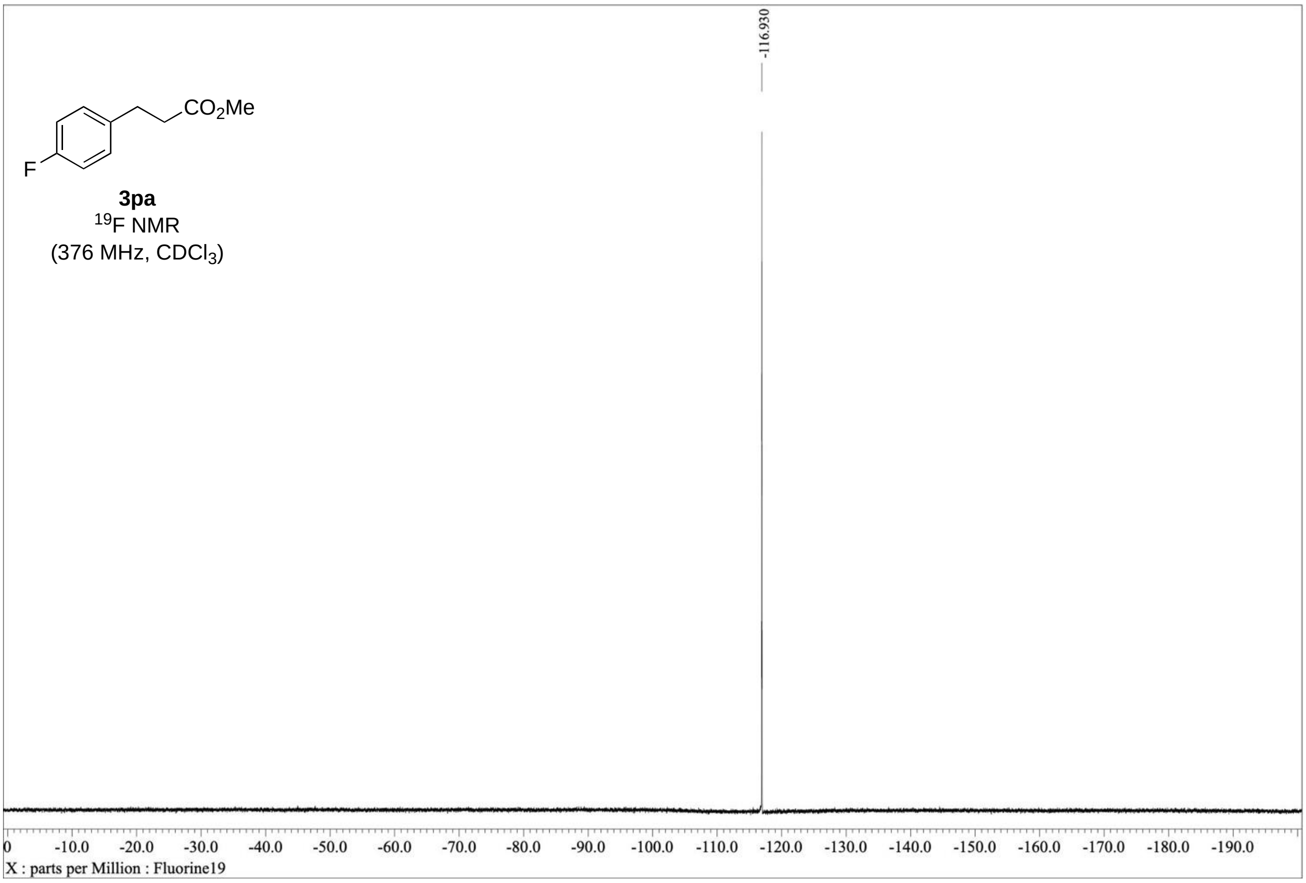

-116.930
CO2Me
F
3pa
19F NMR
(376 MHz, CDCl3)
0 -10.0 -20.0 -30.0 -40.0 -50.0 -60.0 -70.0 -80.0 -90.0 -100.0 -110.0 -120.0 -130.0 -140.0 -150.0 -160.0 -170.0 -180.0 -190.0
X : parts per Million : Fluorine19

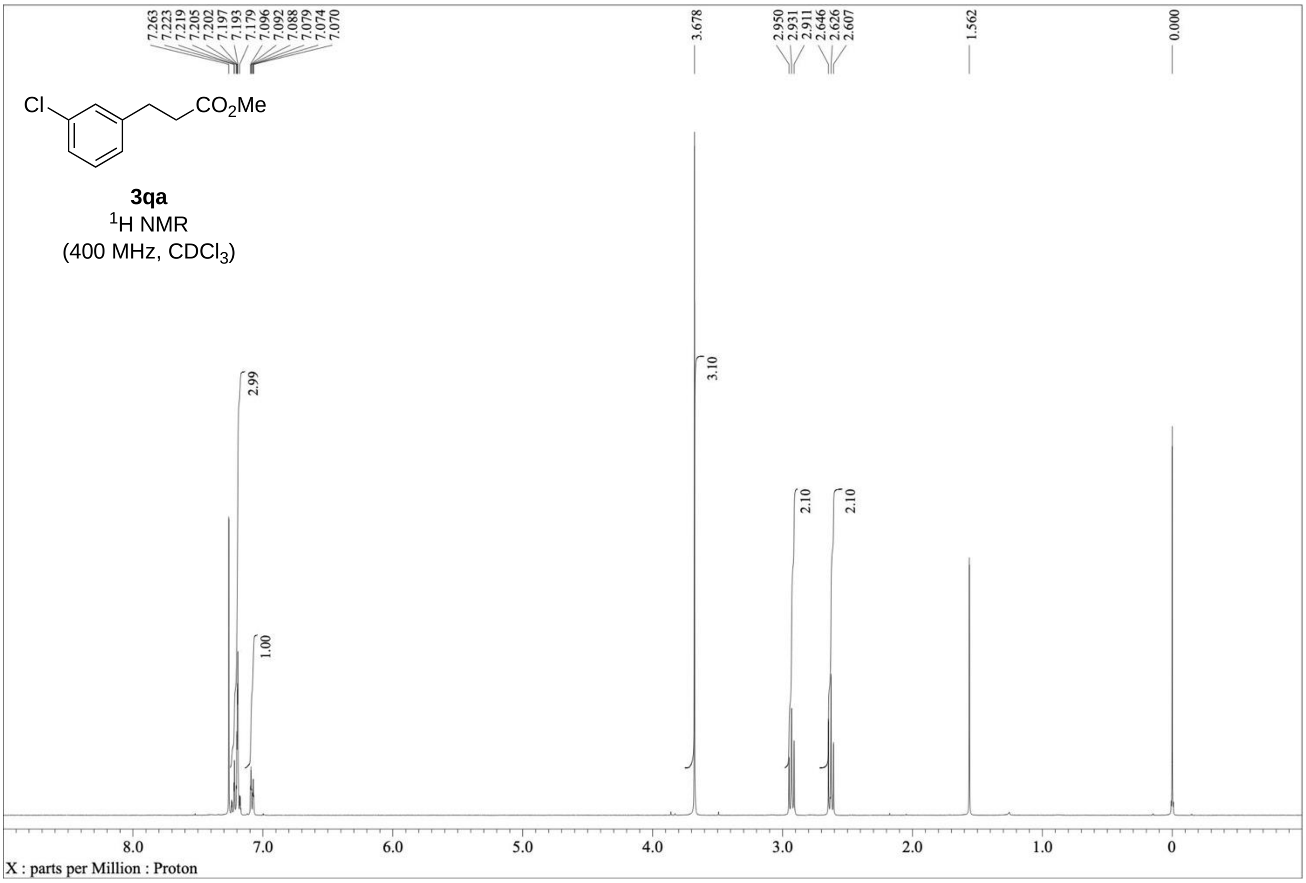
7.263
7.223
7.219
7.205
7.202
7.197
7.193
7.179
7.096
7.092
7.088
7.079
7.074
7.070
3.678
2.950
2.931
2.911
2.646
2.626
2.607
1.562
0.000
Cl
CO2Me
3qa
1H NMR
(400 MHz, CDCl3)
2.99
1.00
3.10
2.10
2.10
8.0
7.0
6.0
5.0
4.0
3.0
2.0
1.0
0
X : parts per Million : Proton

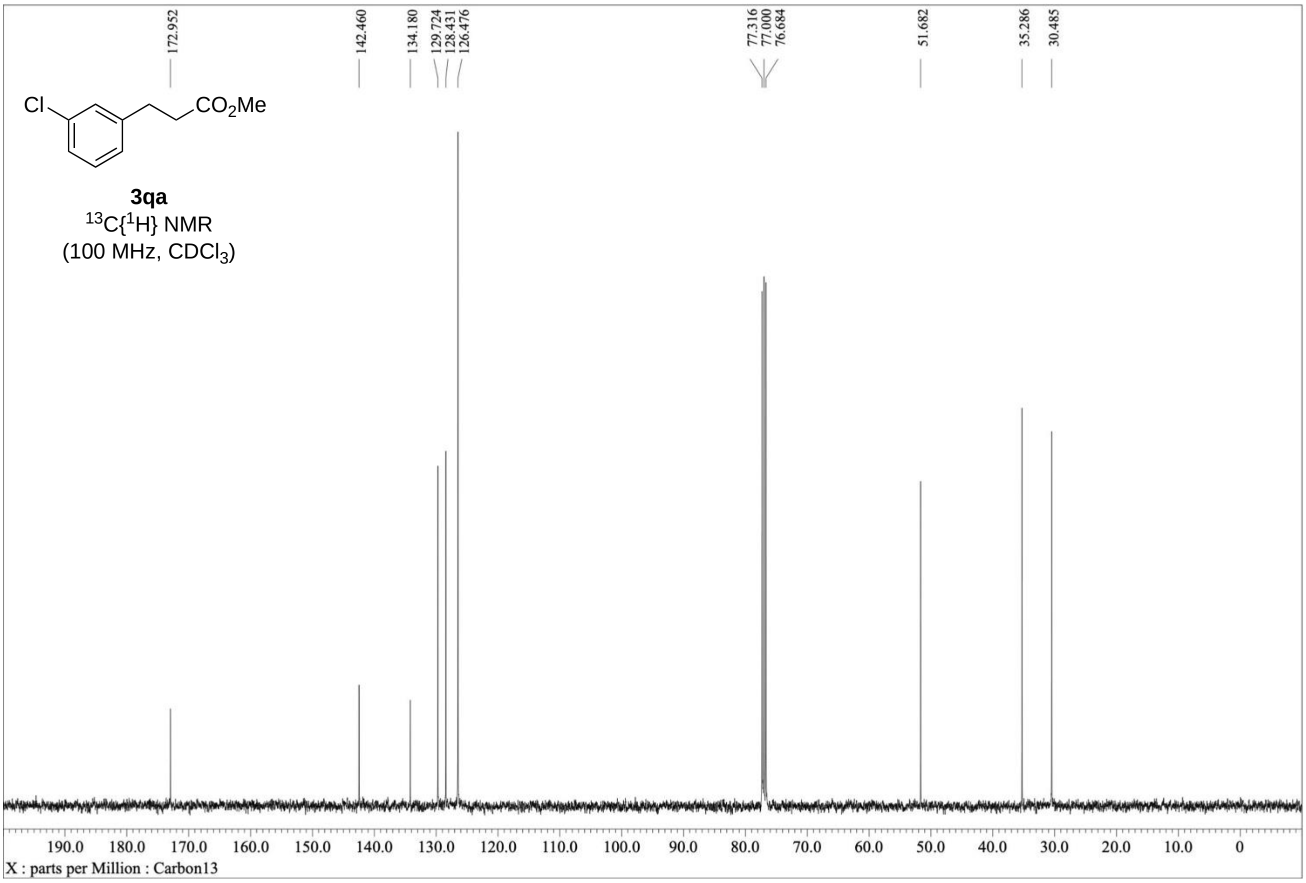
172.952
142.460
134.180
129.724
128.431
126.476
77.316
77.000
76.684
51.682
35.286
30.485
Cl
CO2Me
3qa
13C{1H} NMR
(100 MHz, CDCl3)
190.0
180.0
170.0
160.0
150.0
140.0
130.0
120.0
110.0
100.0
90.0
80.0
70.0
60.0
50.0
40.0
30.0
20.0
10.0
0
X : parts per Million : Carbon13

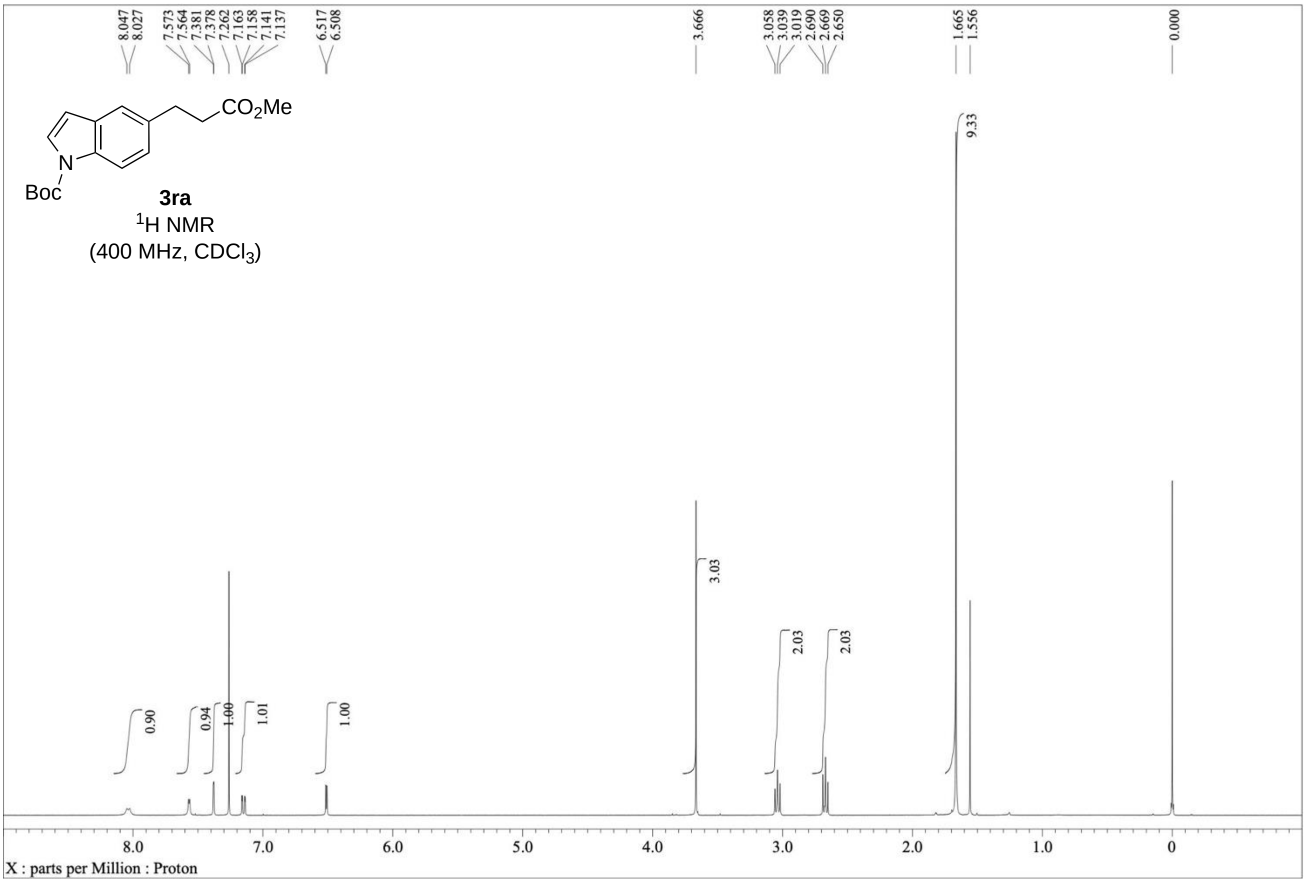
8.047
8.027
7.573
7.564
7.381
7.378
7.262
7.163
7.158
7.141
7.137
6.517
6.508
3.666
3.058
3.039
3.019
2.690
2.669
2.650
1.665
1.556
0.000
CO2Me
N
Boc
3ra
1H NMR
(400 MHz, CDCl3)
9.33
3.03
2.03
2.03
0.90
0.94
1.00
1.01
1.00
8.0
7.0
6.0
5.0
4.0
3.0
2.0
1.0
0
X : parts per Million : Proton

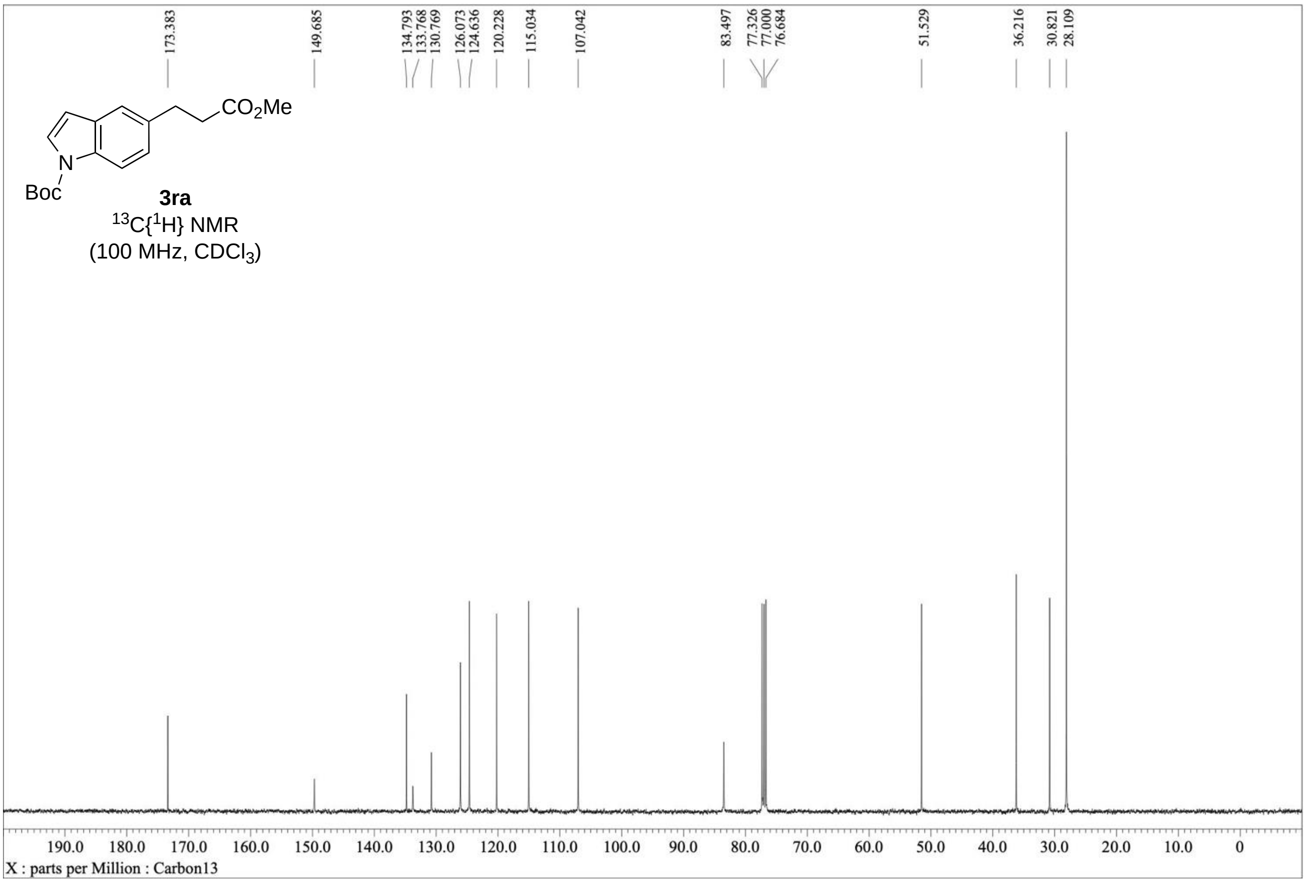
173.383
149.685
134.793
133.768
130.769
126.073
124.636
120.228
115.034
107.042
83.497
77.326
77.000
76.684
51.529
36.216
30.821
28.109
CO2Me
N
Boc
3ra
13C{1H} NMR
(100 MHz, CDCl3)
190.0
180.0
170.0
160.0
150.0
140.0
130.0
120.0
110.0
100.0
90.0
80.0
70.0
60.0
50.0
40.0
30.0
20.0
10.0
0
X : parts per Million : Carbon13

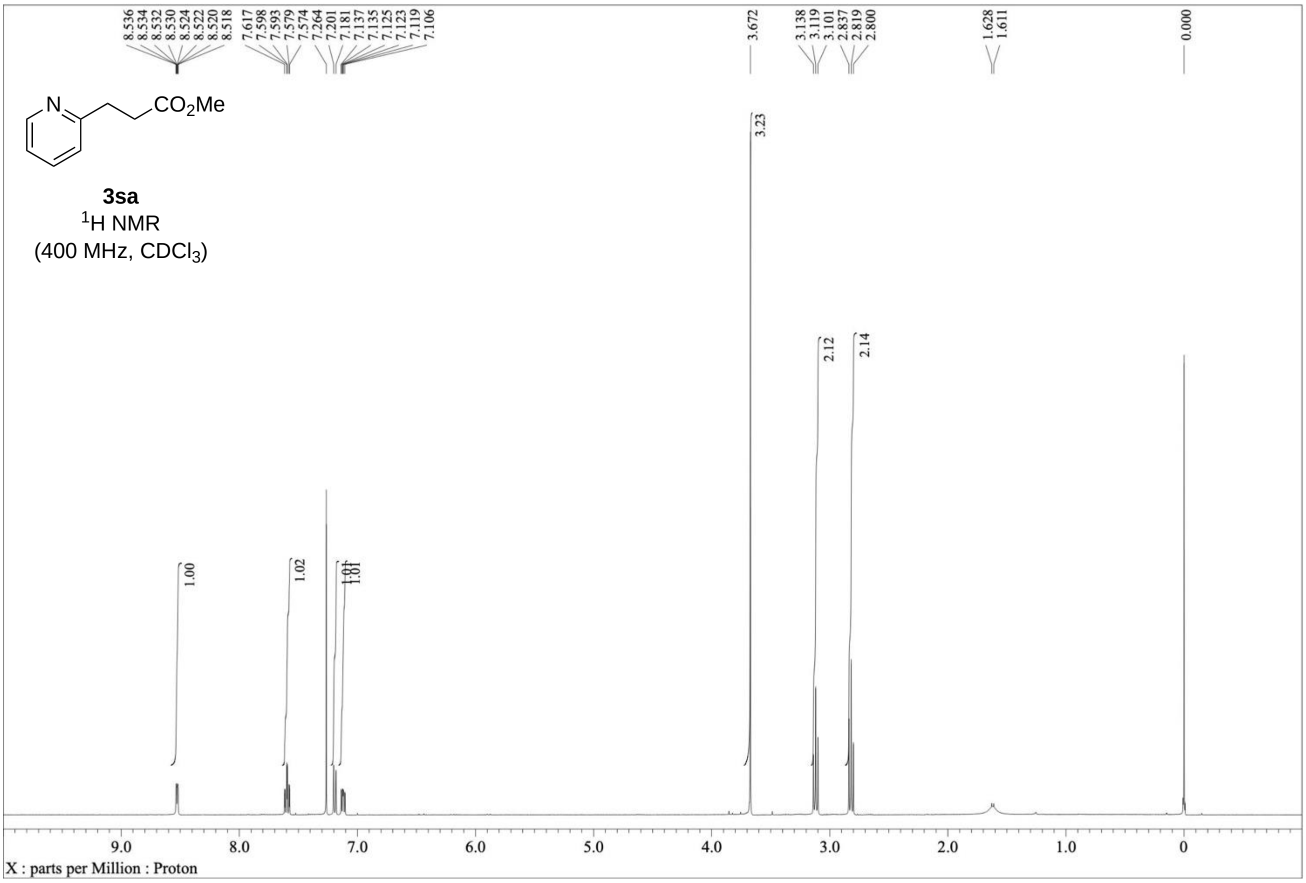
8.536
8.534
8.532
8.530
8.524
8.522
8.520
8.518
7.617
7.598
7.593
7.579
7.574
7.264
7.201
7.181
7.137
7.135
7.125
7.123
7.119
7.106
3.672
3.138
3.119
3.101
2.837
2.819
2.800
1.628
1.611
0.000
CO2Me
N
3sa
1H NMR
(400 MHz, CDCl3)
3.23
2.12
2.14
1.00
1.02
1.01
1.01
9.0
8.0
7.0
6.0
5.0
4.0
3.0
2.0
1.0
0
X : parts per Million : Proton

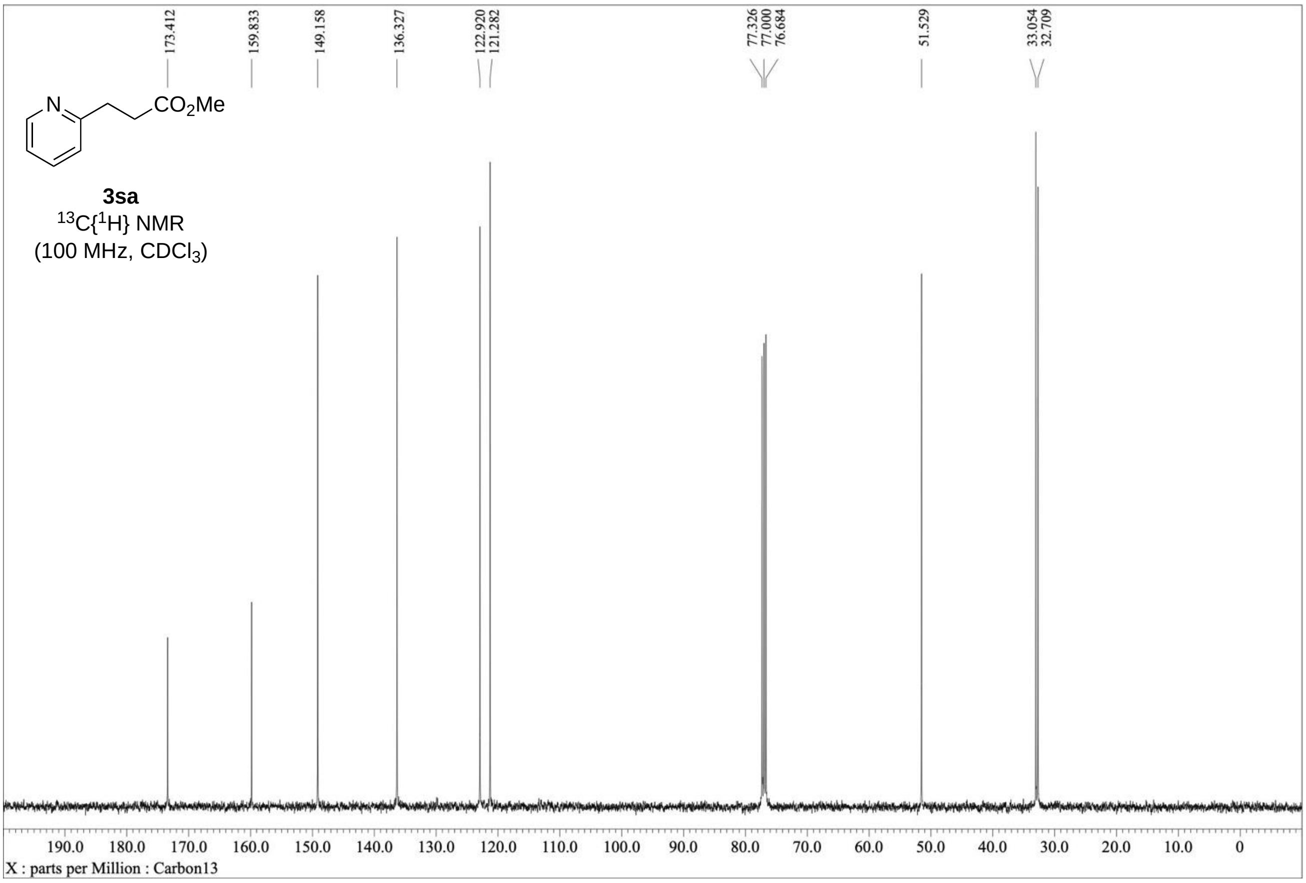
173.412
159.833
149.158
136.327
122.920
121.282
77.326
77.000
76.684
51.529
33.054
32.709
CO2Me
N
3sa
13C{1H} NMR
(100 MHz, CDCl3)
190.0
180.0
170.0
160.0
150.0
140.0
130.0
120.0
110.0
100.0
90.0
80.0
70.0
60.0
50.0
40.0
30.0
20.0
10.0
0
X : parts per Million : Carbon13

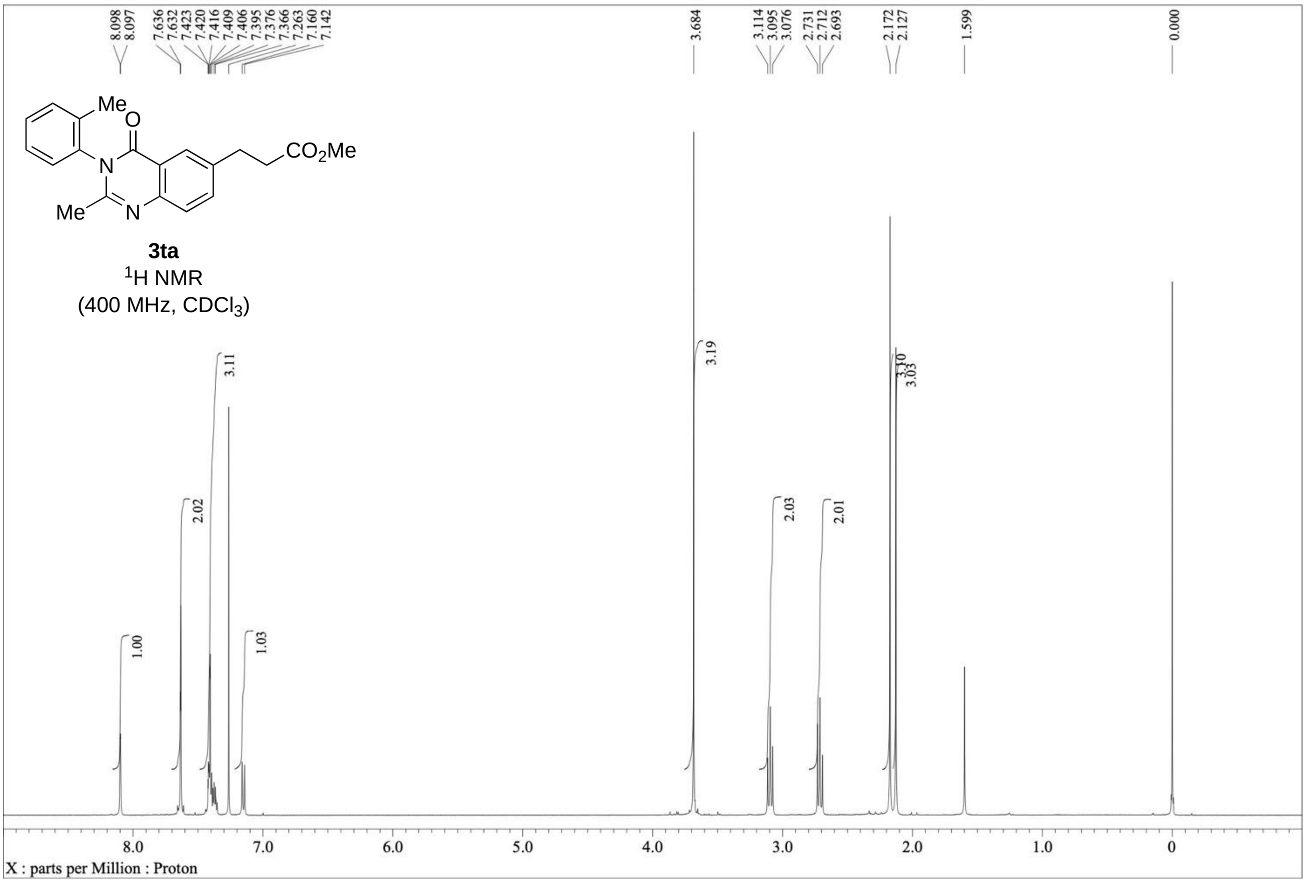
3ta
1H NMR
(400 MHz, CDCl3)
X : parts per Million : Proton

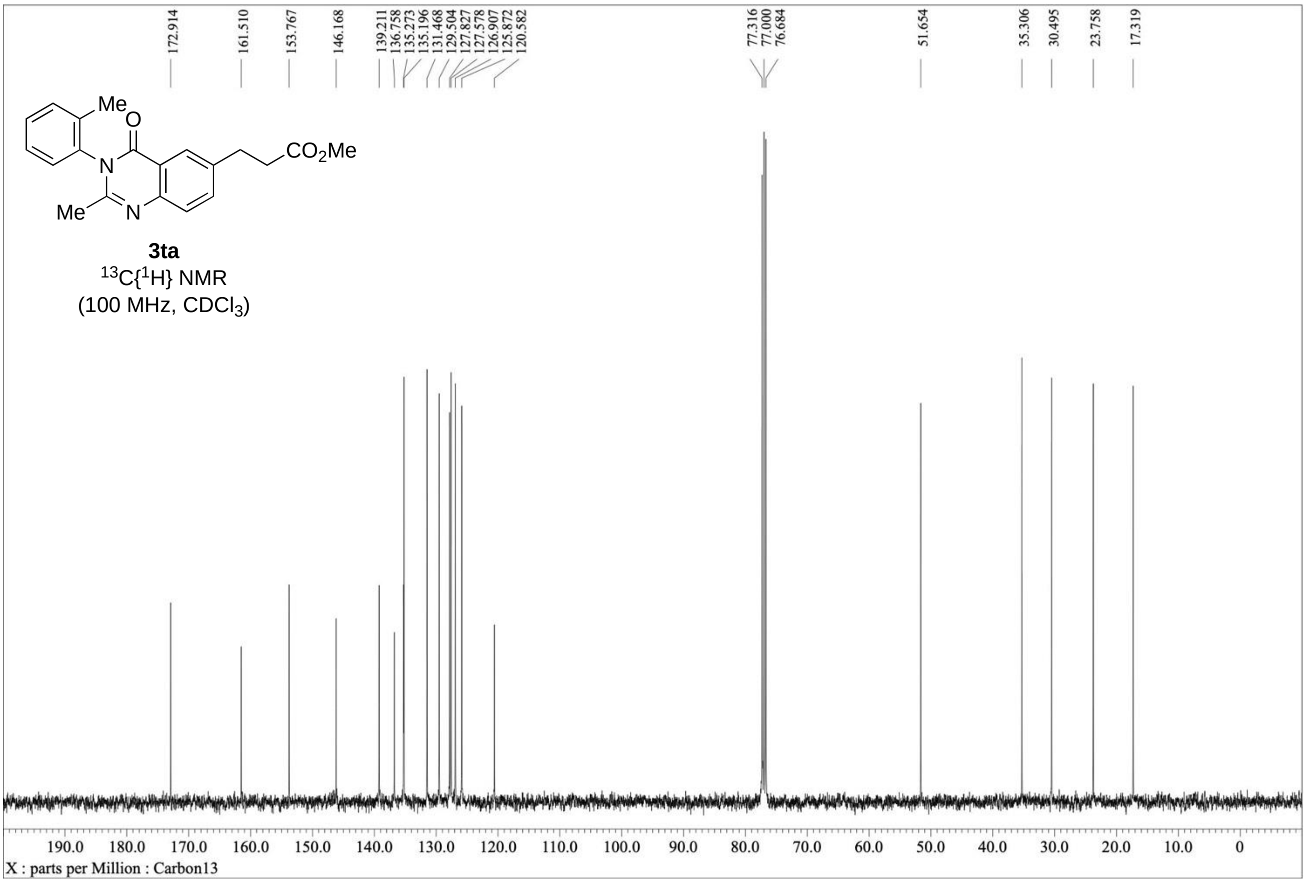
3ta
13C{1H} NMR
(100 MHz, CDCl3)
X : parts per Million : Carbon13

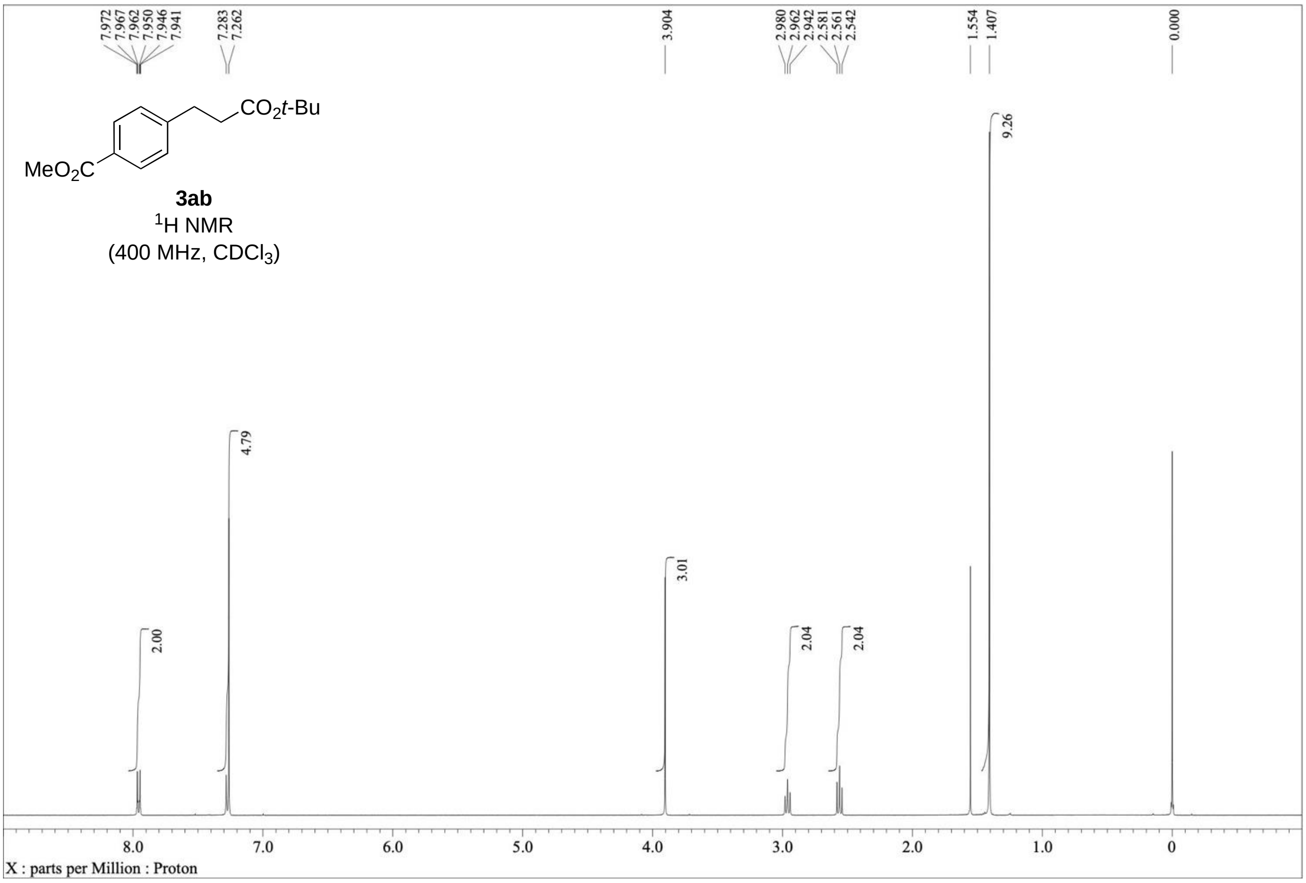

7.972
7.967
7.962
7.950
7.946
7.941
7.283
7.262
3.904
2.980
2.962
2.942
2.581
2.561
2.542
1.554
1.407
0.000
CO2t-Bu
MeO2C
3ab
1H NMR
(400 MHz, CDCl3)
9.26
4.79
3.01
2.00
2.04
2.04
8.0
7.0
6.0
5.0
4.0
3.0
2.0
1.0
0
X : parts per Million : Proton

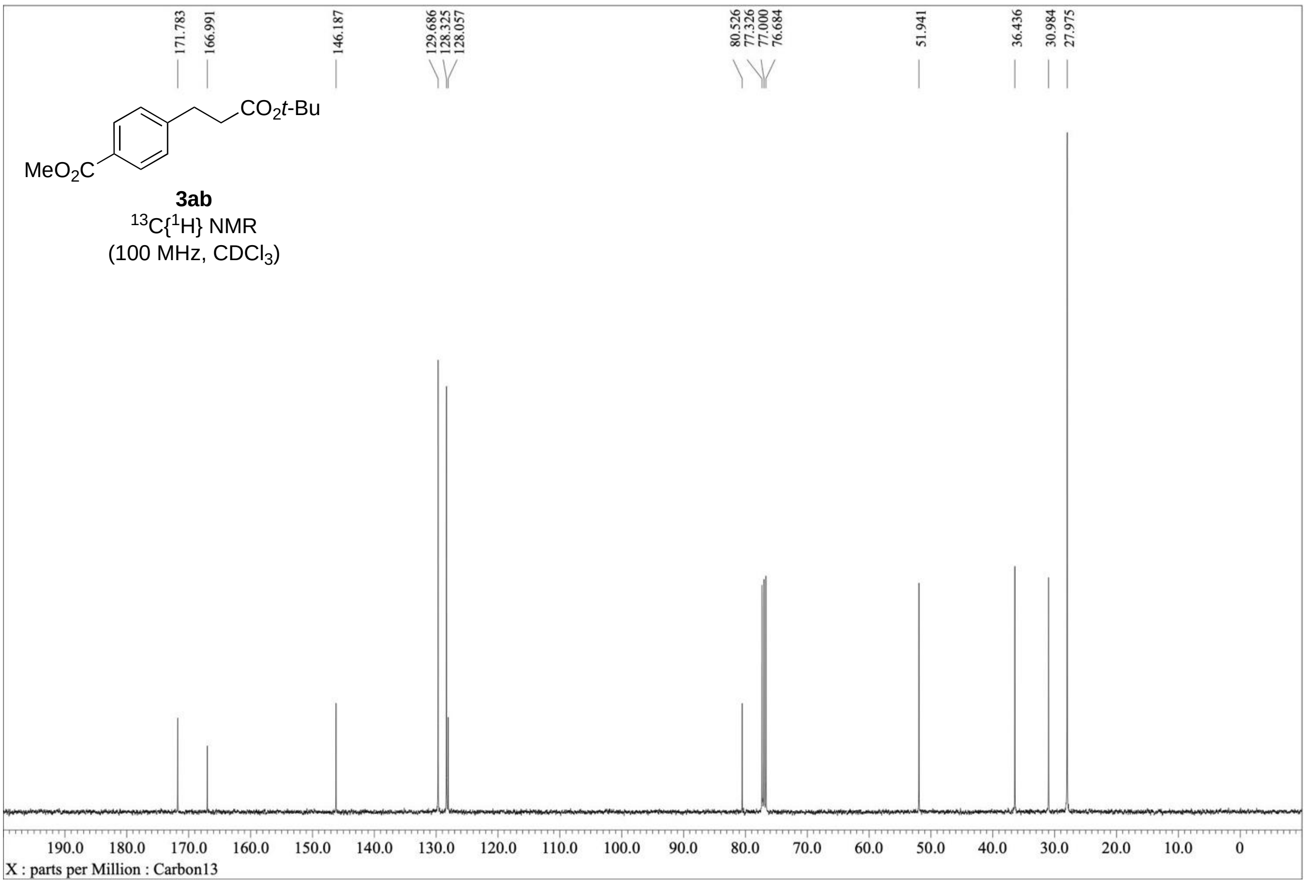

171.783
166.991
146.187
129.686
128.325
128.057
80.526
77.326
77.000
76.684
51.941
36.436
30.984
27.975
CO2t-Bu
MeO2C
3ab
13C{1H} NMR
(100 MHz, CDCl3)
190.0
180.0
170.0
160.0
150.0
140.0
130.0
120.0
110.0
100.0
90.0
80.0
70.0
60.0
50.0
40.0
30.0
20.0
10.0
0
X : parts per Million : Carbon13

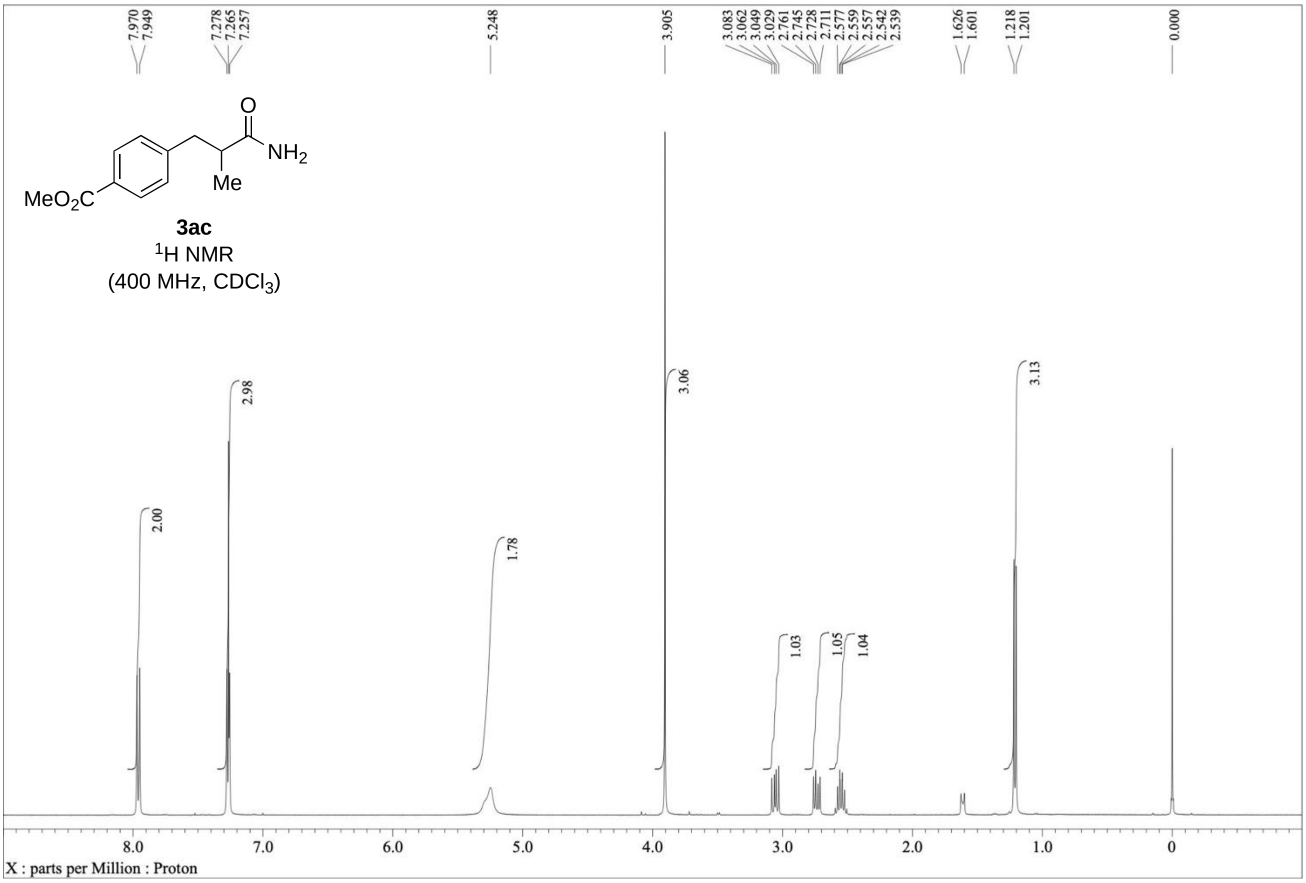
7.970
7.949
7.278
7.265
7.257
5.248
3.905
3.083
3.062
3.049
3.029
2.761
2.745
2.728
2.711
2.577
2.559
2.557
2.542
2.539
1.626
1.601
1.218
1.201
0.000
O
NH2
Me
MeO2C
3ac
1H NMR
(400 MHz, CDCl3)
2.00
2.98
1.78
3.06
1.03
1.05
1.04
3.13
8.0
7.0
6.0
5.0
4.0
3.0
2.0
1.0
0
X : parts per Million : Proton

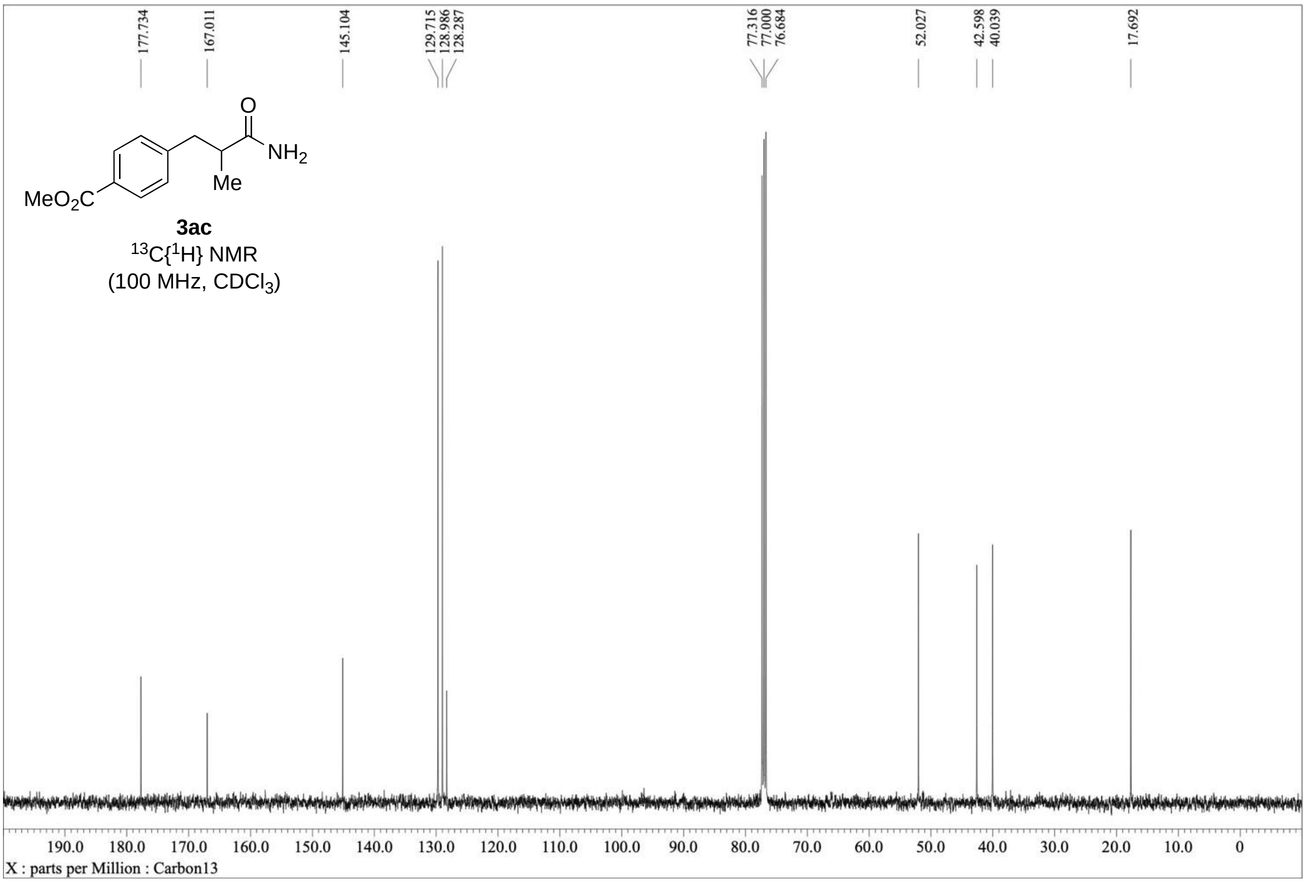
177.734
167.011
145.104
129.715
128.986
128.287
77.316
77.000
76.684
52.027
42.598
40.039
17.692
O
NH2
Me
MeO2C
3ac
13C{1H} NMR
(100 MHz, CDCl3)
190.0
180.0
170.0
160.0
150.0
140.0
130.0
120.0
110.0
100.0
90.0
80.0
70.0
60.0
50.0
40.0
30.0
20.0
10.0
0
X : parts per Million : Carbon13

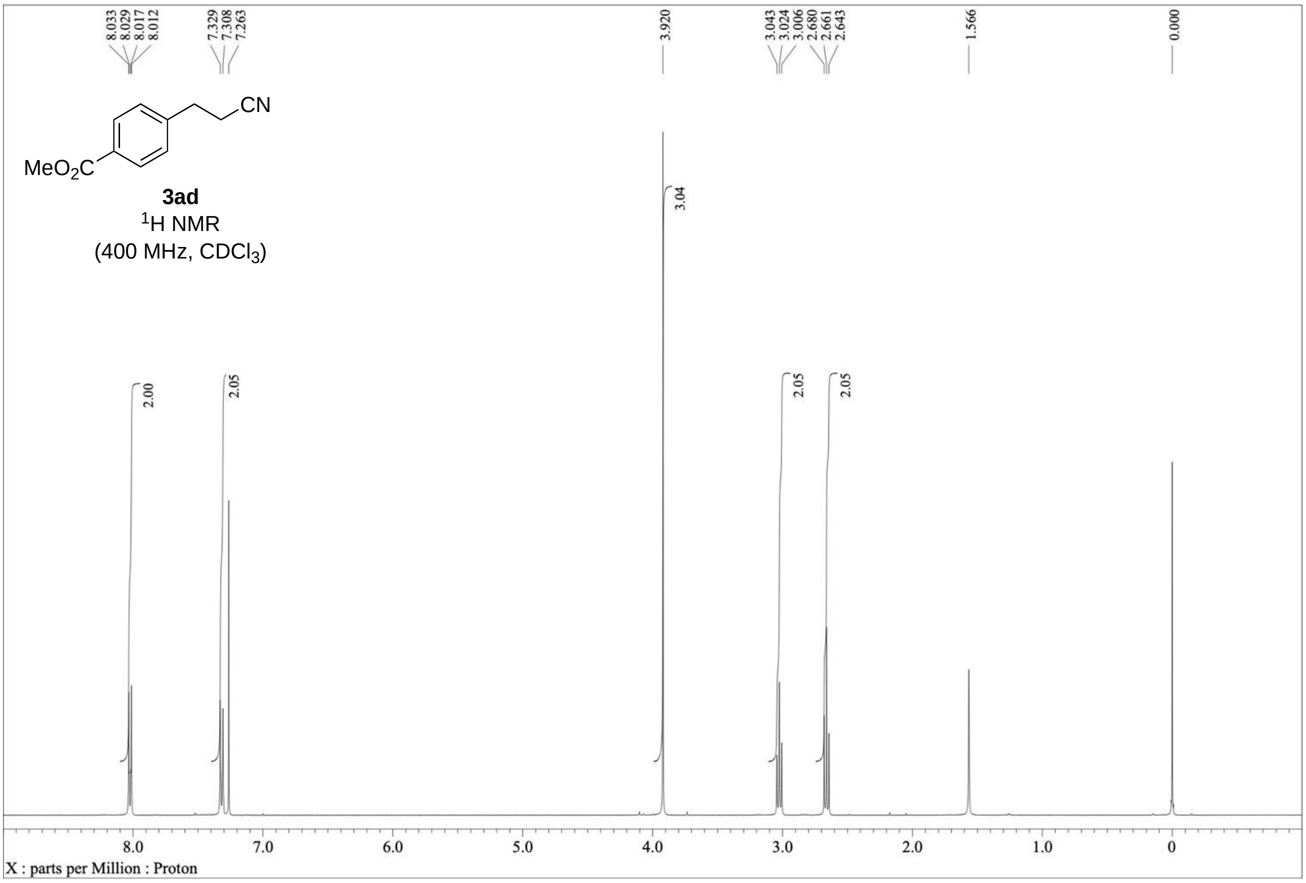
8.033
8.029
8.017
8.012
7.329
7.308
7.263
3.920
3.043
3.024
3.006
2.680
2.661
2.643
1.566
0.000
CN
MeO2C
3ad
1H NMR
(400 MHz, CDCl3)
3.04
2.00
2.05
2.05
2.05
8.0
7.0
6.0
5.0
4.0
3.0
2.0
1.0
0
X : parts per Million : Proton

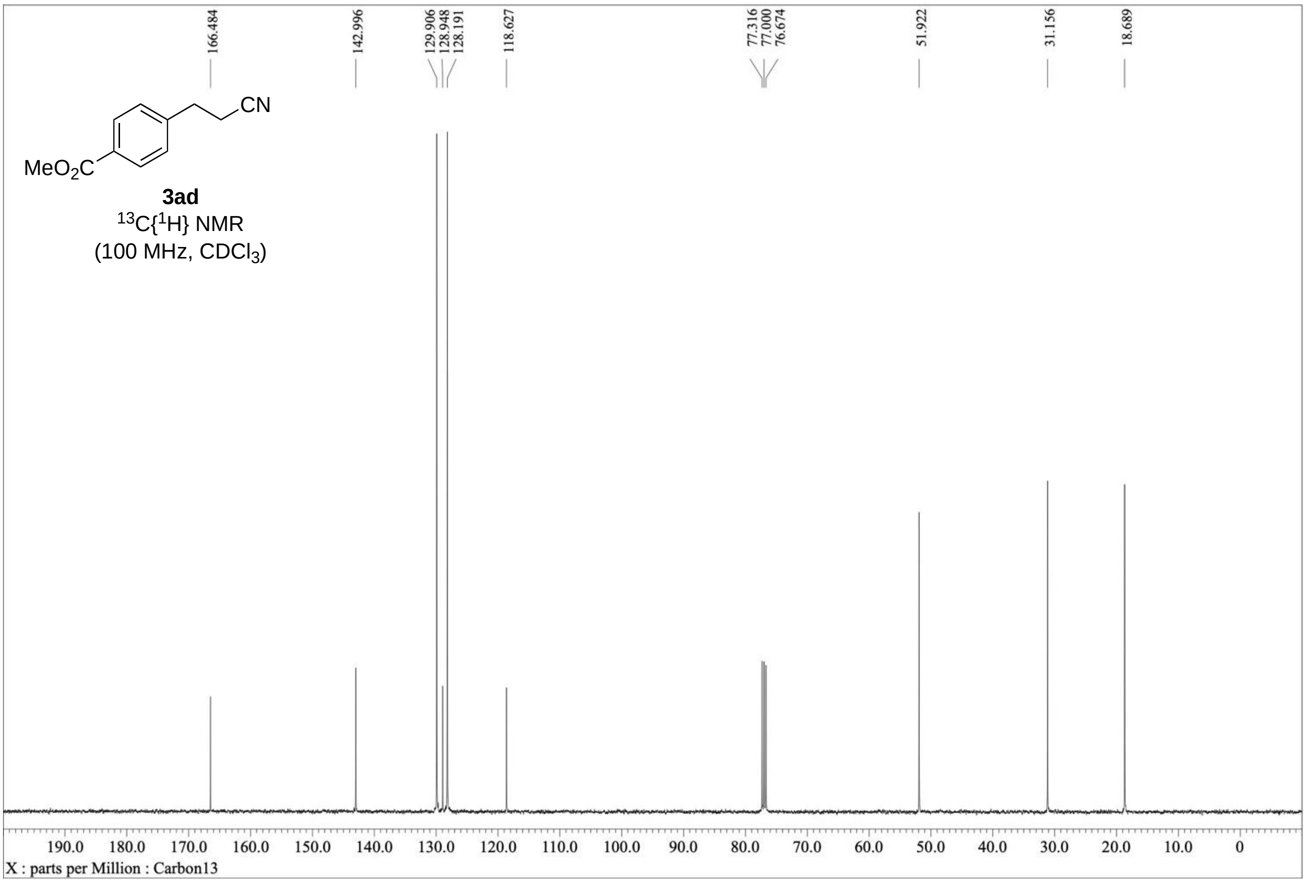
166.484
142.996
129.906
128.948
128.191
118.627
77.316
77.000
76.674
51.922
31.156
18.689
CN
MeO2C
3ad
13C{1H} NMR
(100 MHz, CDCl3)
190.0
180.0
170.0
160.0
150.0
140.0
130.0
120.0
110.0
100.0
90.0
80.0
70.0
60.0
50.0
40.0
30.0
20.0
10.0
0
X : parts per Million : Carbon13

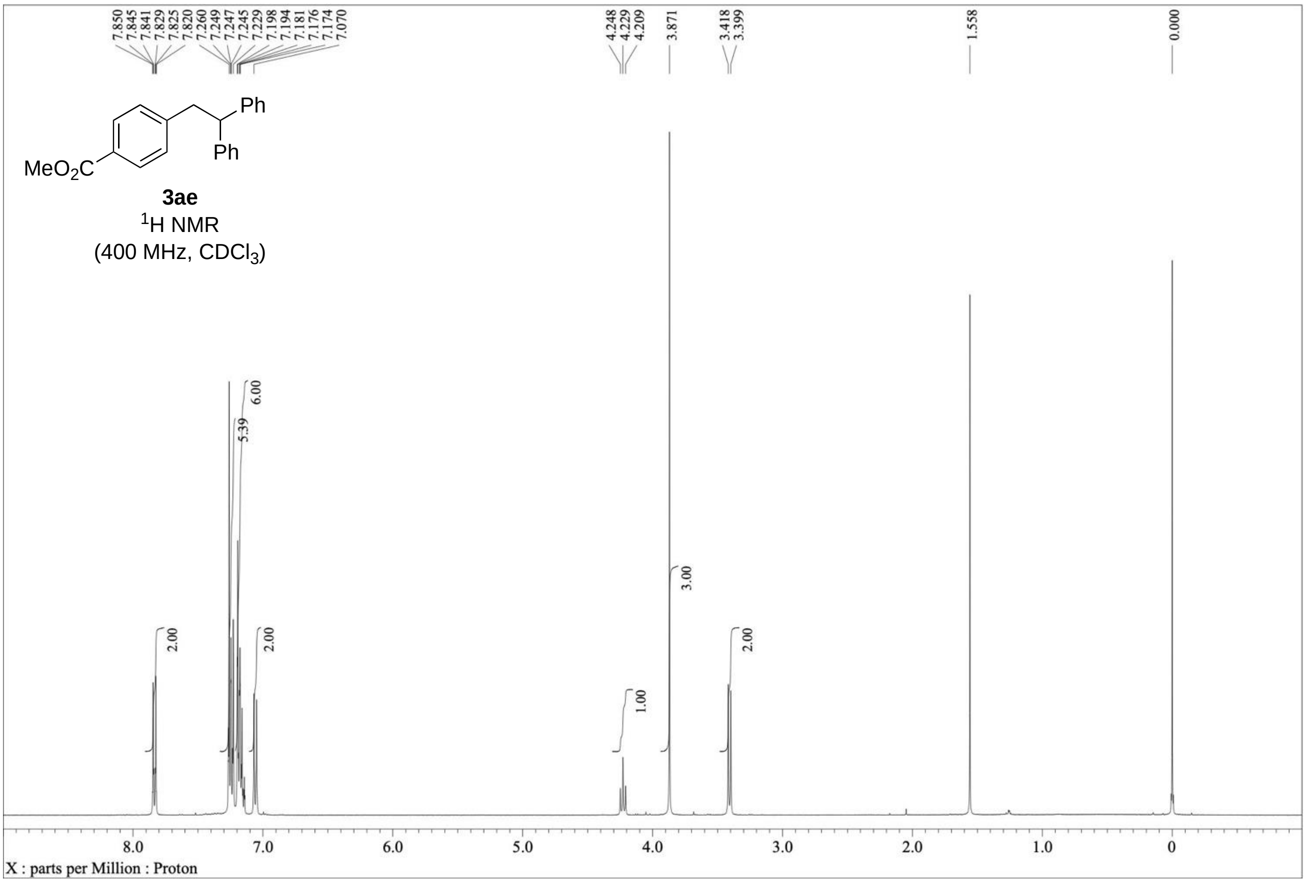
7.850
7.845
7.841
7.829
7.825
7.820
7.260
7.249
7.247
7.245
7.229
7.198
7.194
7.181
7.176
7.174
7.070
4.248
4.229
4.209
3.871
3.418
3.399
1.558
0.000
Ph
Ph
MeO2C
3ae
1H NMR
(400 MHz, CDCl3)
2.00
5.39
6.00
2.00
1.00
3.00
2.00
8.0
7.0
6.0
5.0
4.0
3.0
2.0
1.0
0
X : parts per Million : Proton

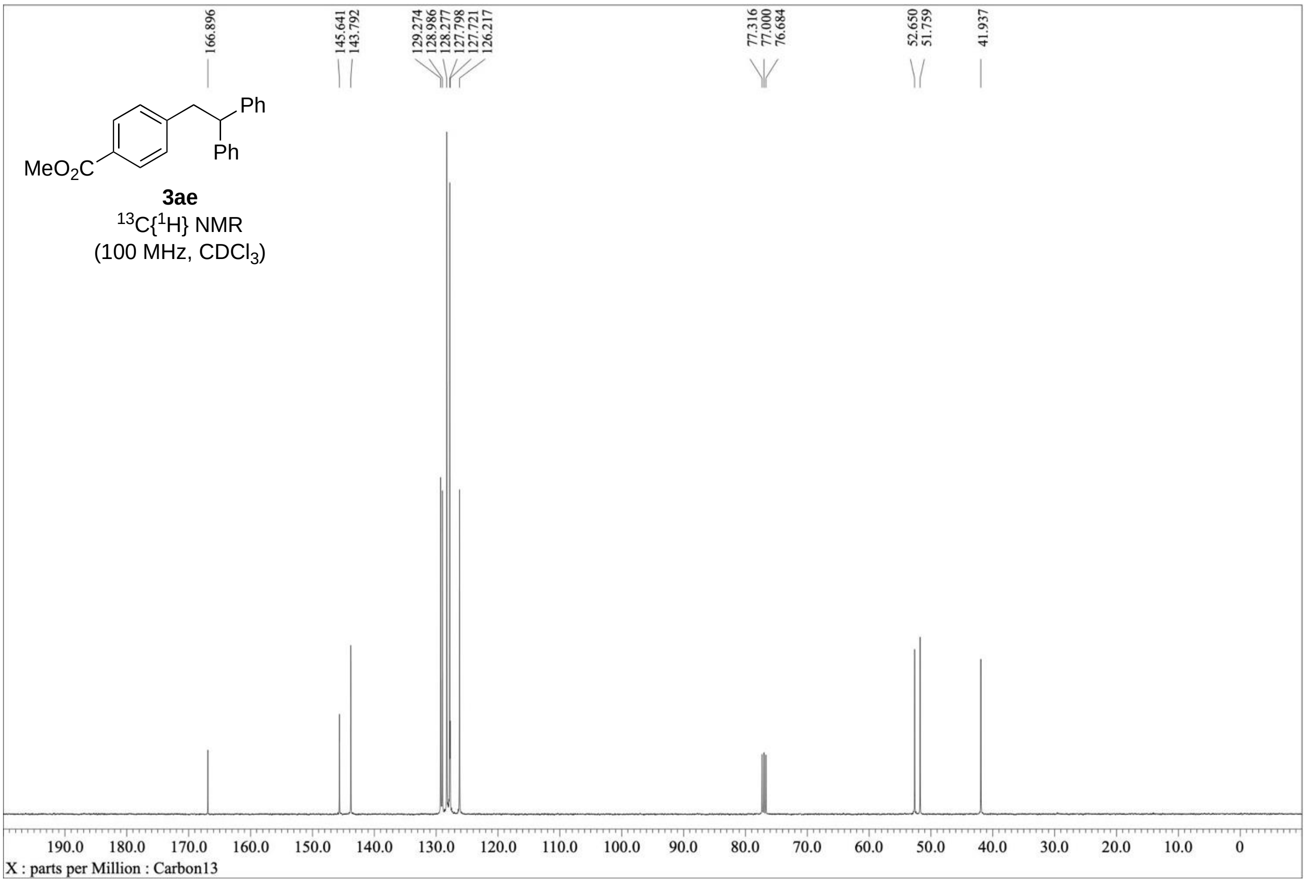
166.896
145.641
143.792
129.274
128.986
128.277
127.798
127.721
126.217
77.316
77.000
76.684
52.650
51.759
41.937
Ph
Ph
MeO2C
3ae
13C{1H} NMR
(100 MHz, CDCl3)
190.0
180.0
170.0
160.0
150.0
140.0
130.0
120.0
110.0
100.0
90.0
80.0
70.0
60.0
50.0
40.0
30.0
20.0
10.0
0
X : parts per Million : Carbon13

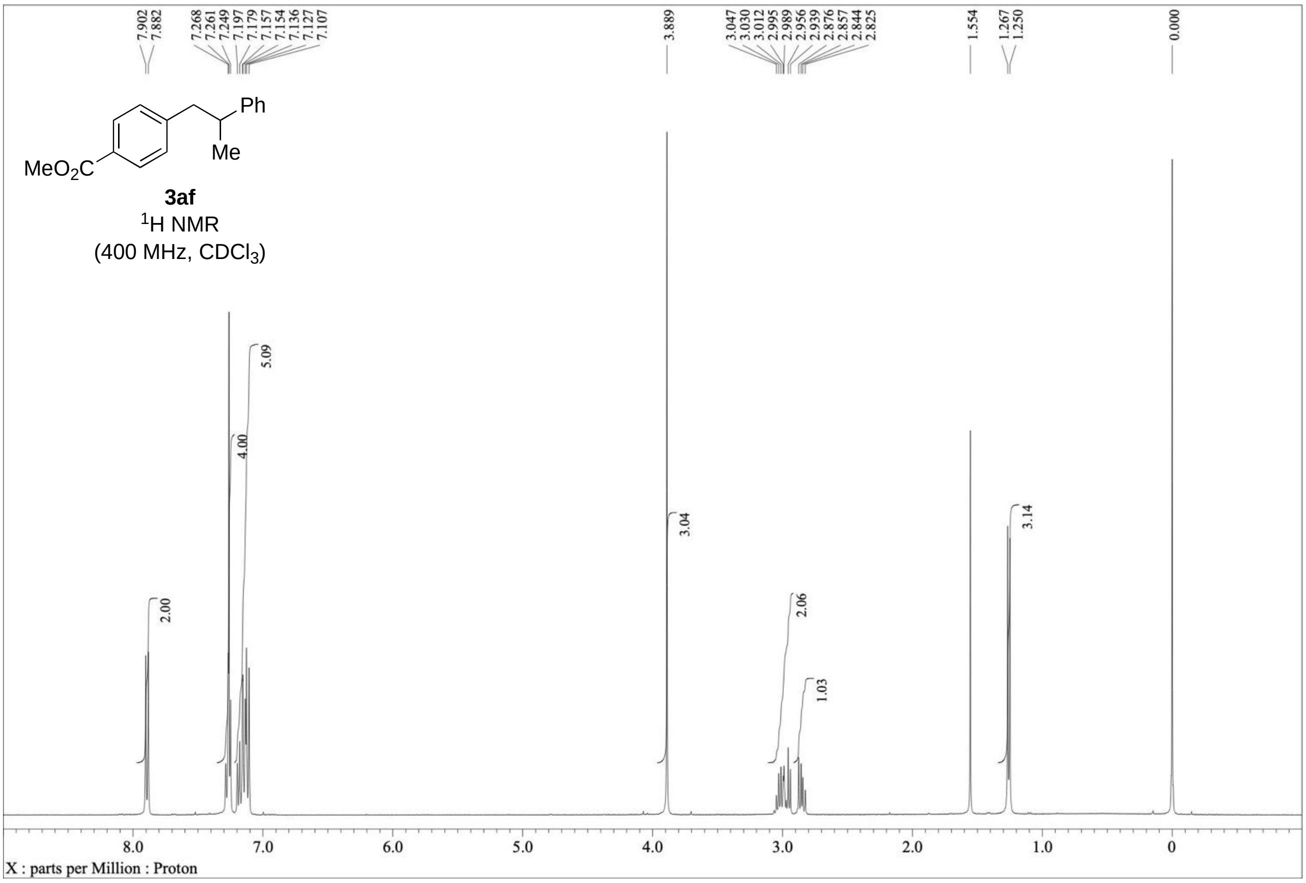
7.902
7.882
7.268
7.261
7.249
7.197
7.179
7.157
7.154
7.136
7.127
7.107
3.889
3.047
3.030
3.012
2.995
2.989
2.956
2.939
2.876
2.857
2.844
2.825
1.554
1.267
1.250
0.000
Ph
Me
MeO2C
3af
1H NMR
(400 MHz, CDCl3)
5.09
4.00
3.04
3.14
2.00
2.06
1.03
8.0
7.0
6.0
5.0
4.0
3.0
2.0
1.0
0
X : parts per Million : Proton

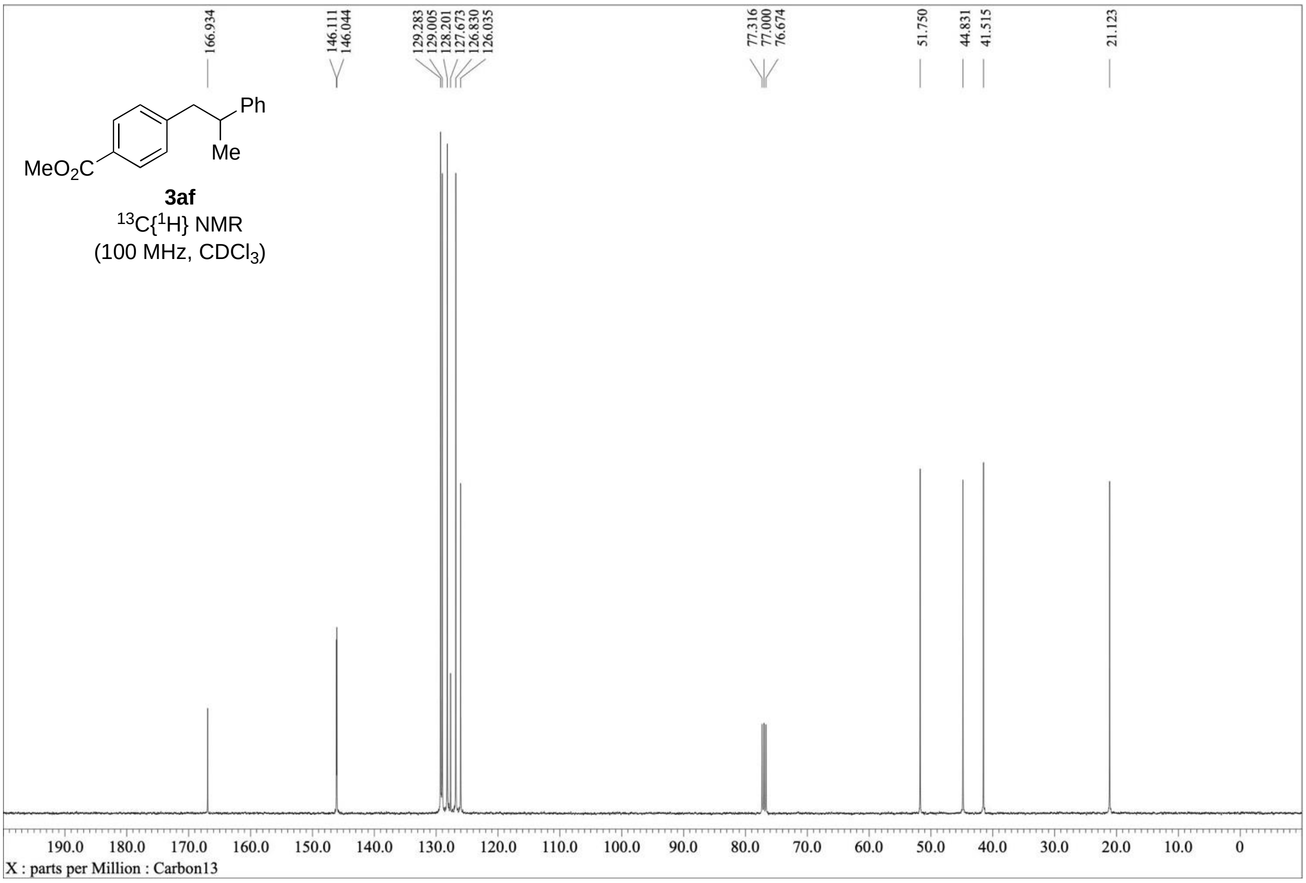
166.934
146.111
146.044
129.283
129.005
128.201
127.673
126.830
126.035
77.316
77.000
76.674
51.750
44.831
41.515
21.123
Ph
Me
MeO2C
3af
13C{1H} NMR
(100 MHz, CDCl3)
190.0
180.0
170.0
160.0
150.0
140.0
130.0
120.0
110.0
100.0
90.0
80.0
70.0
60.0
50.0
40.0
30.0
20.0
10.0
0
X : parts per Million : Carbon13

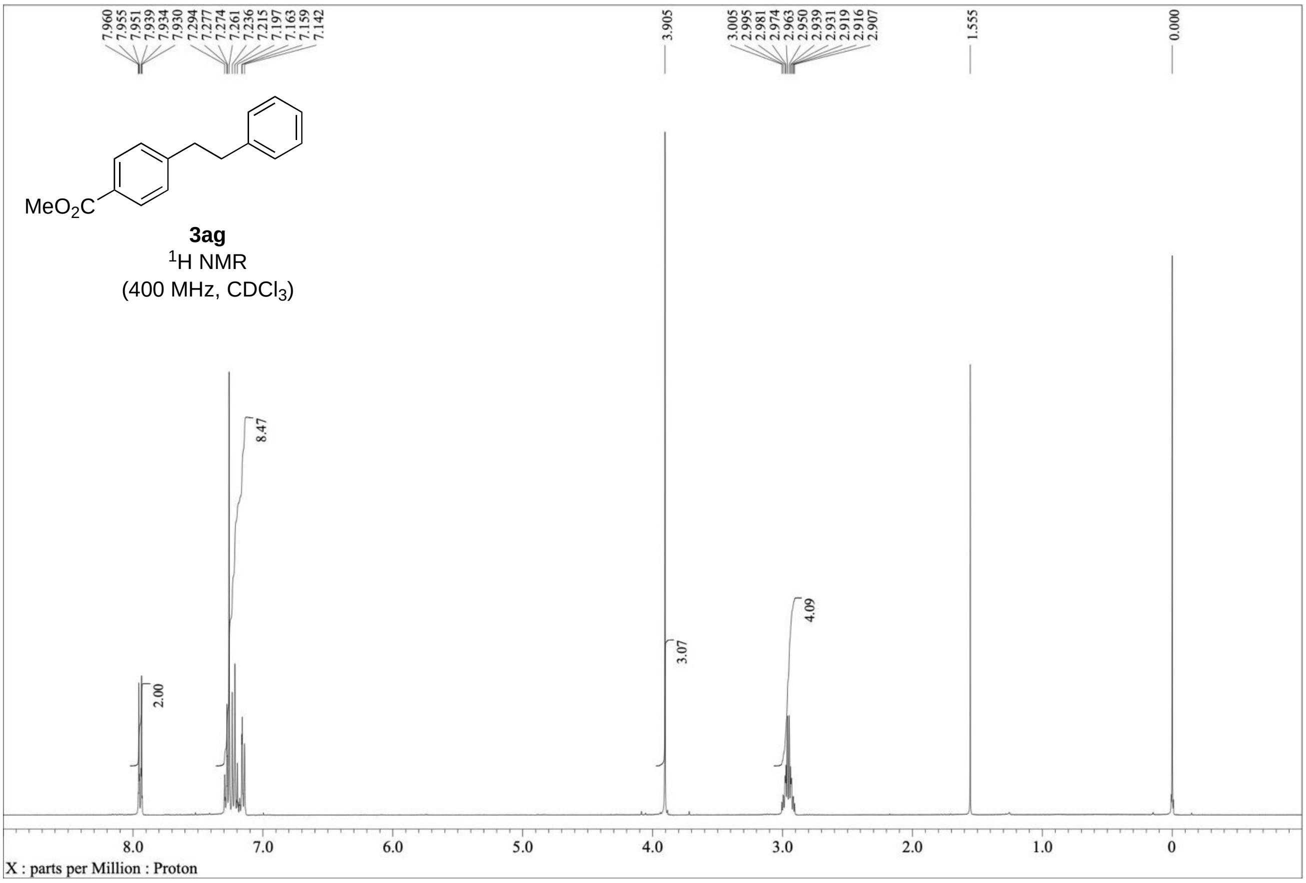
7.960
7.955
7.951
7.939
7.934
7.930
7.294
7.277
7.274
7.261
7.236
7.215
7.197
7.163
7.159
7.142
3.905
3.005
2.995
2.981
2.974
2.963
2.950
2.939
2.931
2.919
2.916
2.907
1.555
0.000
MeO2C
3ag
1H NMR
(400 MHz, CDCl3)
8.47
4.09
3.07
2.00
8.0
7.0
6.0
5.0
4.0
3.0
2.0
1.0
0
X : parts per Million : Proton

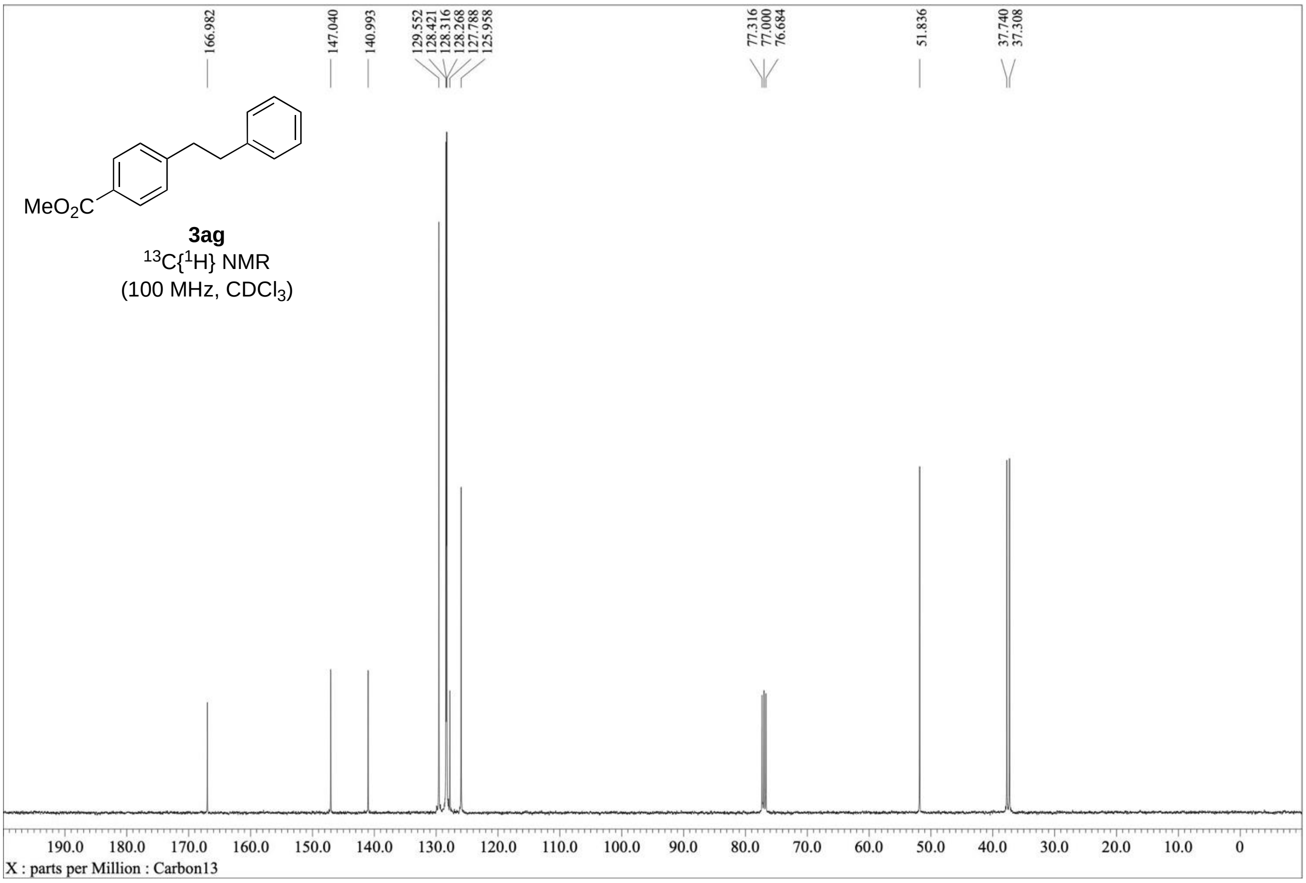
166.982
147.040
140.993
129.552
128.421
128.316
128.268
127.788
125.958
77.316
77.000
76.684
51.836
37.740
37.308
MeO2C
3ag
13C{1H} NMR
(100 MHz, CDCl3)
190.0
180.0
170.0
160.0
150.0
140.0
130.0
120.0
110.0
100.0
90.0
80.0
70.0
60.0
50.0
40.0
30.0
20.0
10.0
0
X : parts per Million : Carbon13

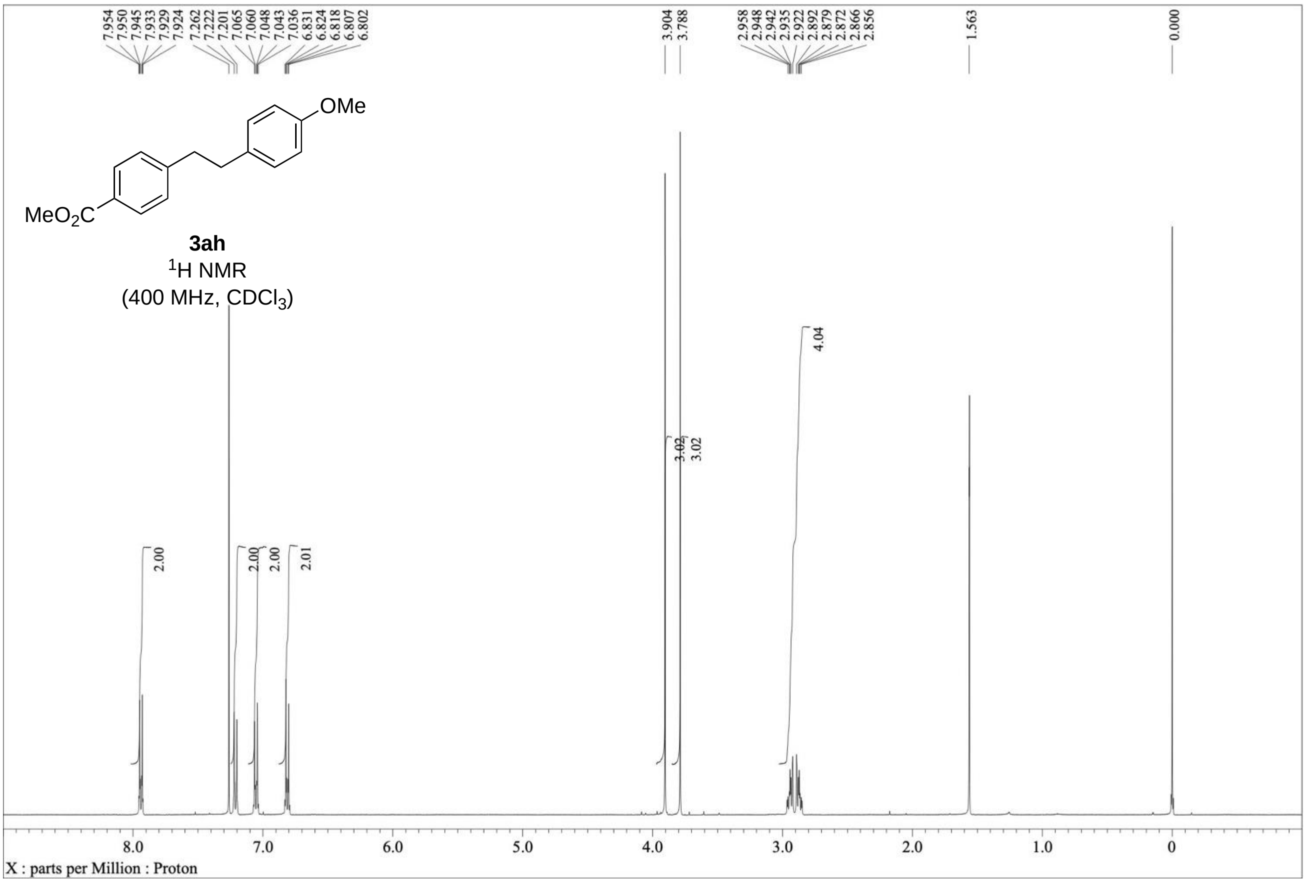
7.954 7.950 7.945 7.933 7.929 7.924 7.262 7.222 7.201 7.065 7.060 7.048 7.043 7.036 6.831 6.824 6.818 6.807 6.802
3.904 3.788
2.958 2.948 2.942 2.935 2.922 2.892 2.879 2.872 2.866 2.856
1.563
0.000
OMe
MeO2C
3ah
1H NMR
(400 MHz, CDCl3)
2.00 2.00 2.00 2.01 3.02 3.02 4.04
8.0 7.0 6.0 5.0 4.0 3.0 2.0 1.0 0
X : parts per Million : Proton

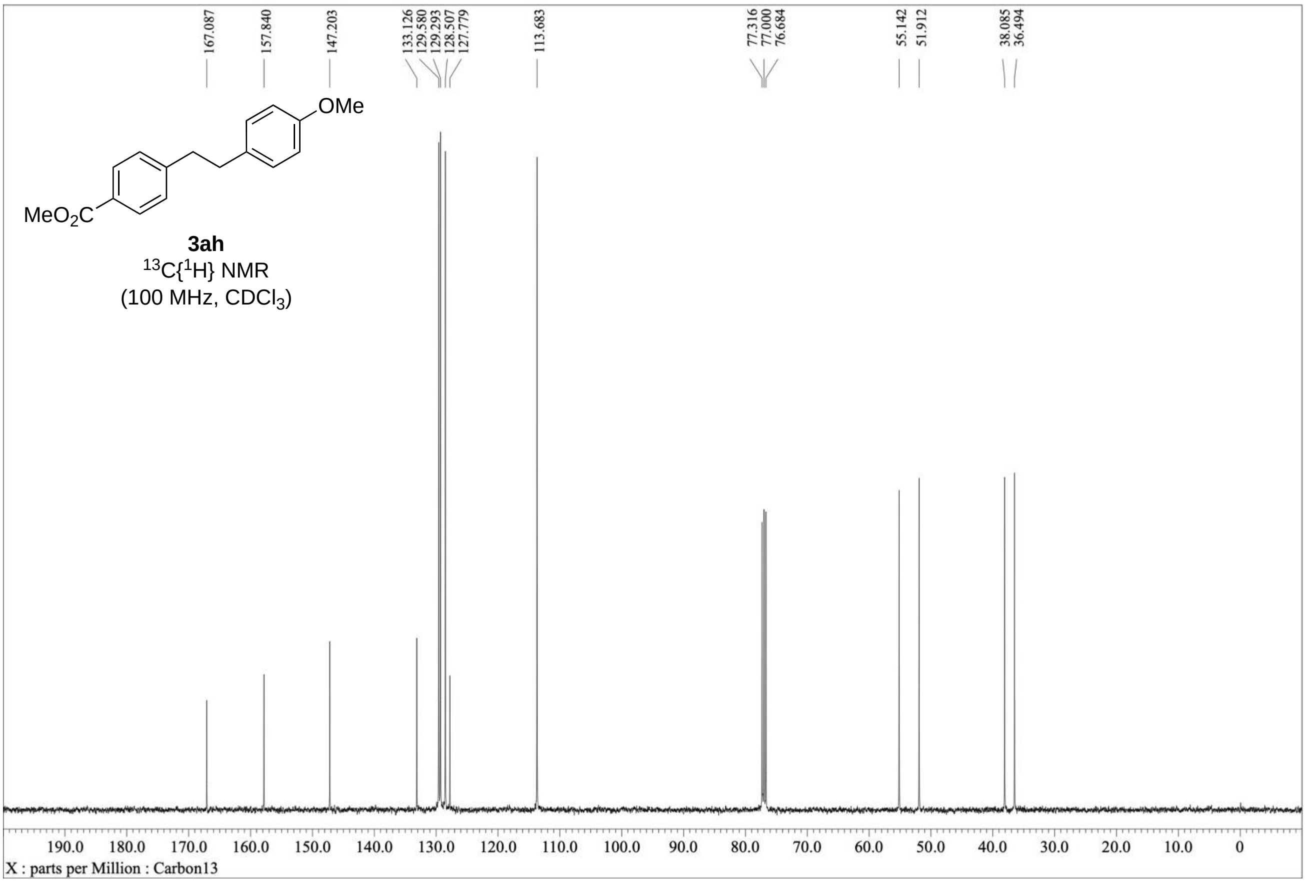
167.087 157.840 147.203 133.126 129.580 129.293 128.507 127.779 113.683 77.316 77.000 76.684 55.142 51.912 38.085 36.494
OMe
MeO2C
3ah
13C{1H} NMR
(100 MHz, CDCl3)
190.0 180.0 170.0 160.0 150.0 140.0 130.0 120.0 110.0 100.0 90.0 80.0 70.0 60.0 50.0 40.0 30.0 20.0 10.0 0
X : parts per Million : Carbon13

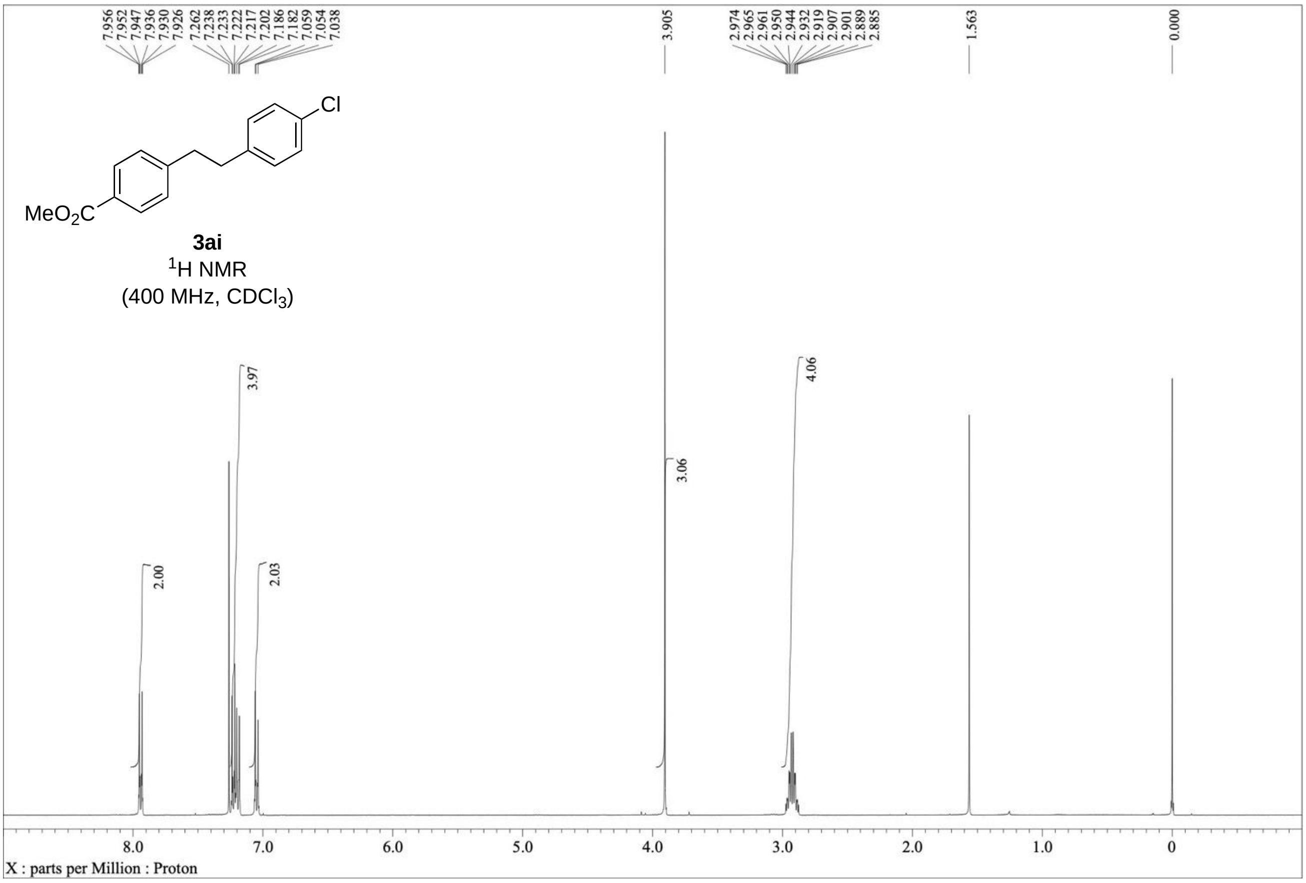
7.956 7.952 7.947 7.936 7.930 7.926 7.262 7.238 7.233 7.222 7.217 7.202 7.186 7.182 7.059 7.054 7.038
3.905
2.974 2.965 2.961 2.950 2.944 2.932 2.919 2.907 2.901 2.889 2.885
1.563
0.000
Cl
MeO2C
3ai
1H NMR
(400 MHz, CDCl3)
2.00
3.97
2.03
3.06
4.06
8.0 7.0 6.0 5.0 4.0 3.0 2.0 1.0 0
X : parts per Million : Proton

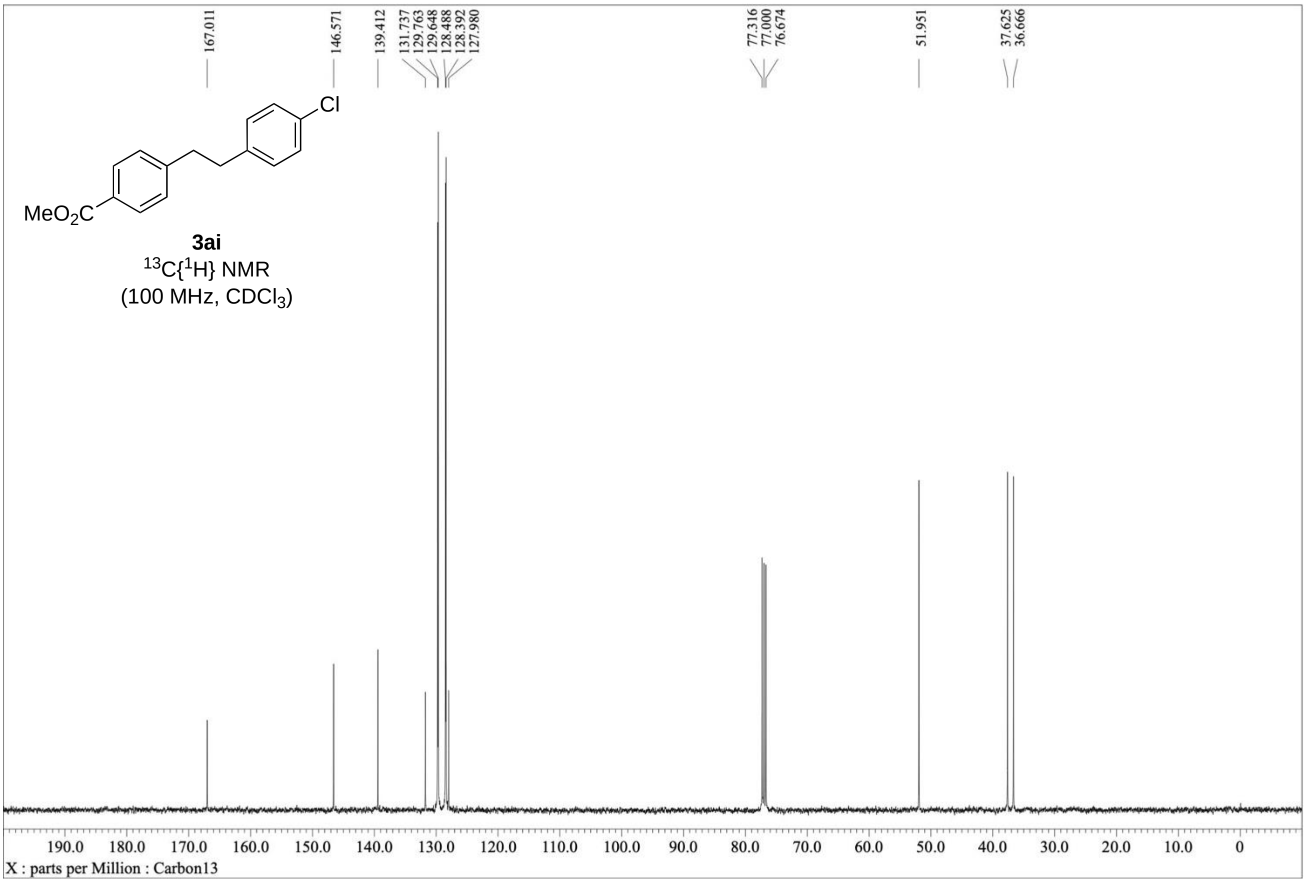
167.011
146.571
139.412
131.737 129.763 129.648 128.488 128.392 127.980
77.316 77.000 76.674
51.951
37.625 36.666
Cl
MeO2C
3ai
13C{1H} NMR
(100 MHz, CDCl3)
190.0 180.0 170.0 160.0 150.0 140.0 130.0 120.0 110.0 100.0 90.0 80.0 70.0 60.0 50.0 40.0 30.0 20.0 10.0 0
X : parts per Million : Carbon13

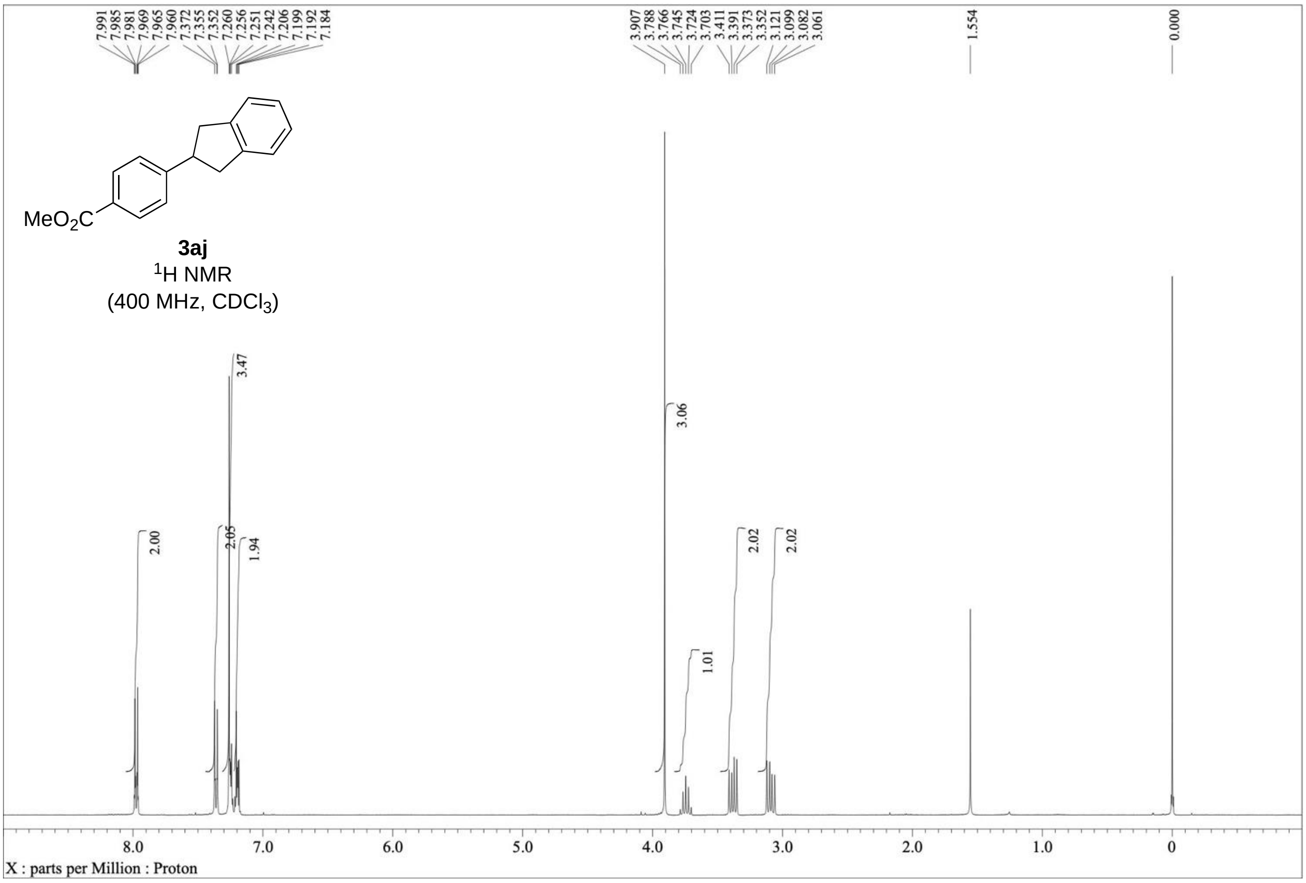
MeO2C
3aj
1H NMR
(400 MHz, CDCl3)
X : parts per Million : Proton

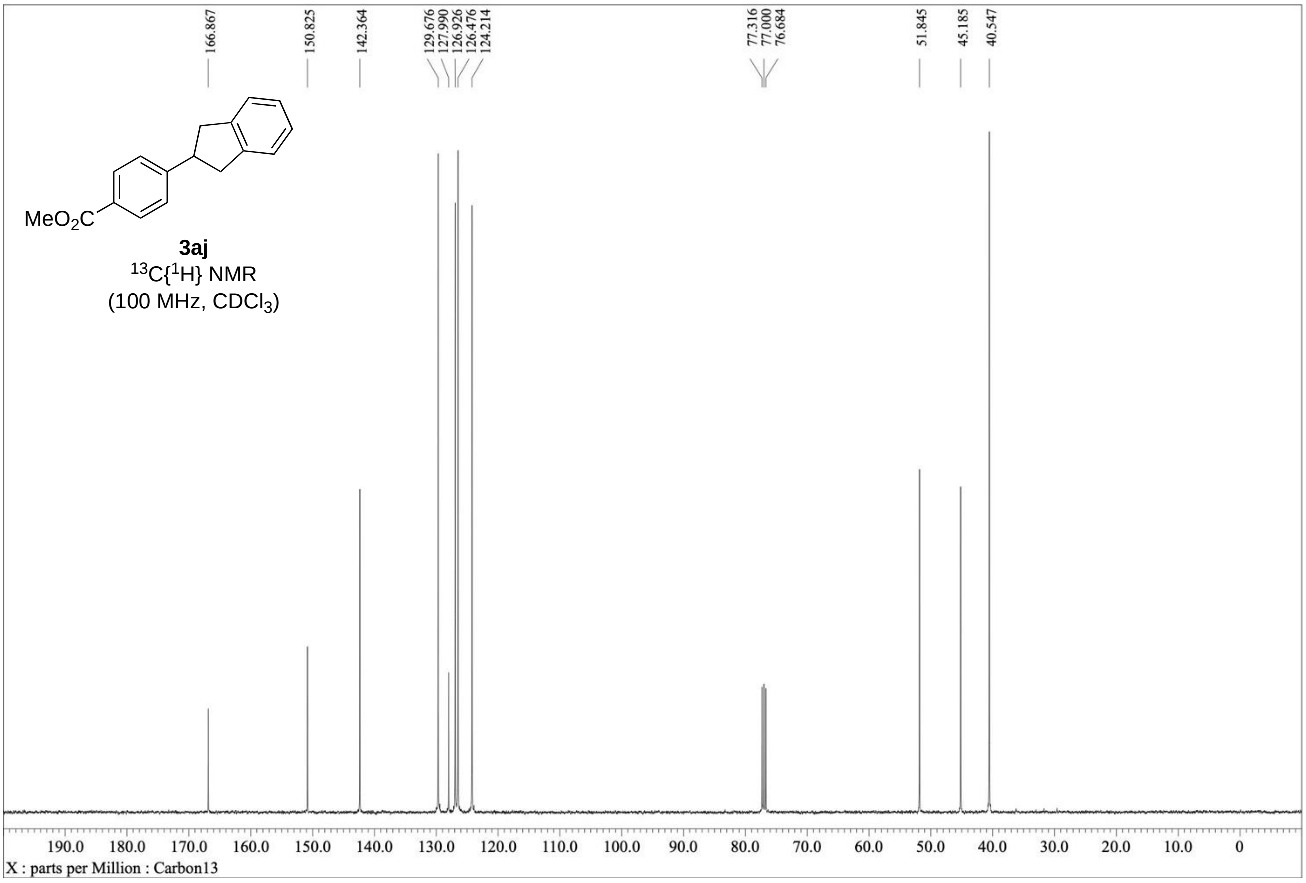
MeO2C
3aj
13C{1H} NMR
(100 MHz, CDCl3)
X : parts per Million : Carbon13

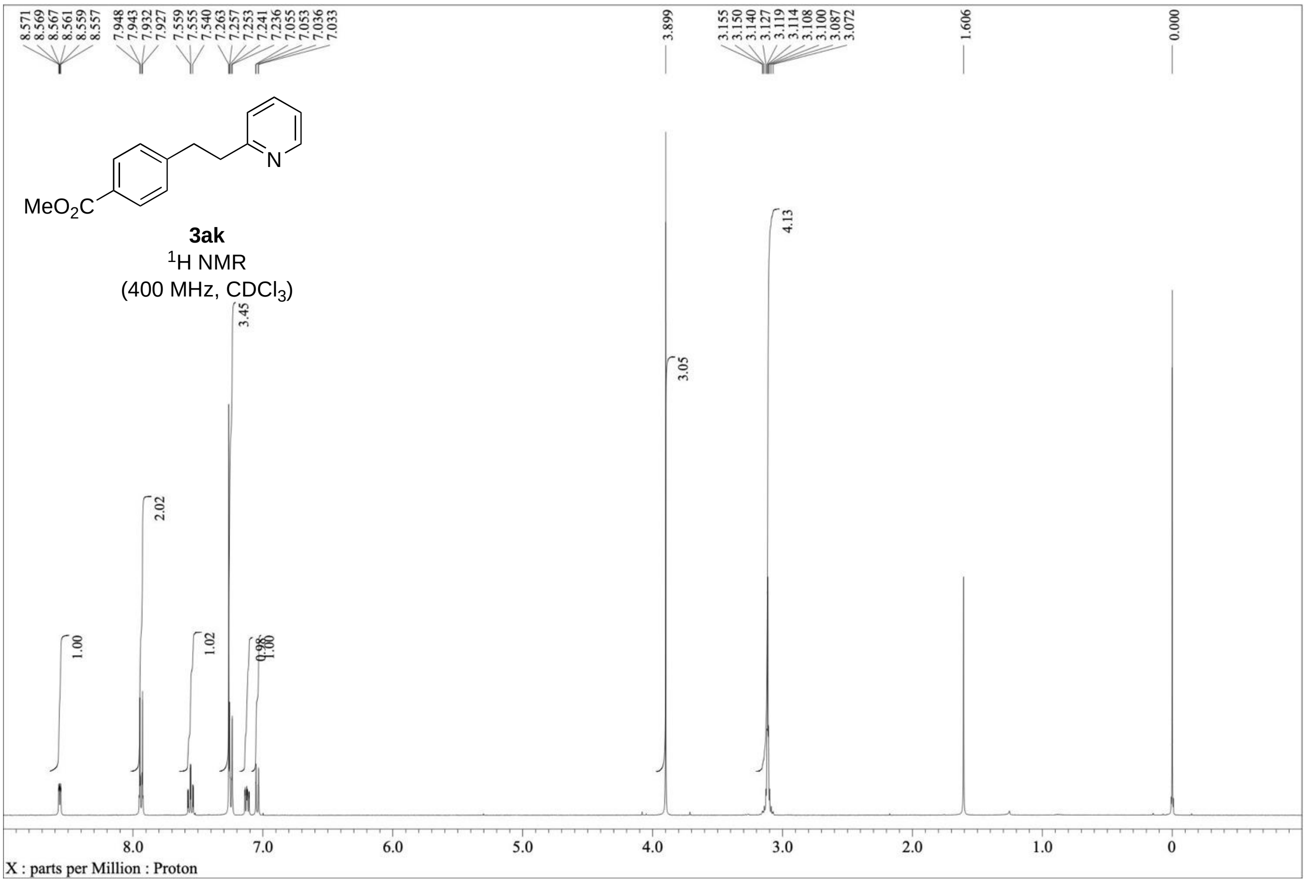
8.571 8.569 8.567 8.561 8.559 8.557 7.948 7.943 7.932 7.927 7.559 7.555 7.540 7.263 7.257 7.253 7.241 7.236 7.055 7.053 7.036 7.033
3.899
3.155 3.150 3.140 3.127 3.119 3.114 3.108 3.100 3.087 3.072
1.606
0.000
MeO2C
N
3ak
1H NMR
(400 MHz, CDCl3)
1.00
2.02
1.02
3.45
0.98
1.00
3.05
4.13
8.0 7.0 6.0 5.0 4.0 3.0 2.0 1.0 0
X : parts per Million : Proton

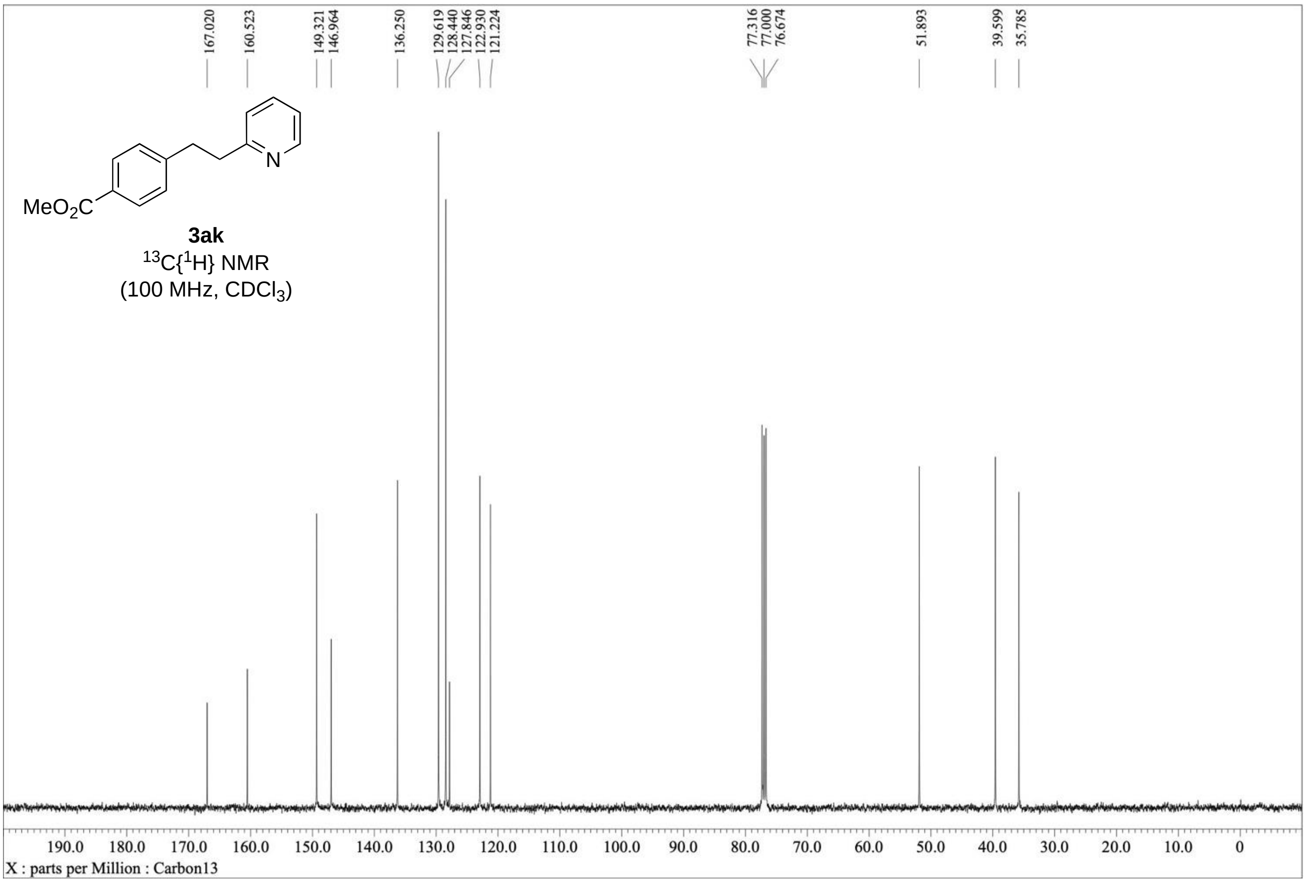
167.020
160.523
149.321 146.964
136.250
129.619 128.440 127.846 122.930 121.224
77.316 77.000 76.674
51.893
39.599 35.785
MeO2C
N
3ak
13C{1H} NMR
(100 MHz, CDCl3)
190.0 180.0 170.0 160.0 150.0 140.0 130.0 120.0 110.0 100.0 90.0 80.0 70.0 60.0 50.0 40.0 30.0 20.0 10.0 0
X : parts per Million : Carbon13

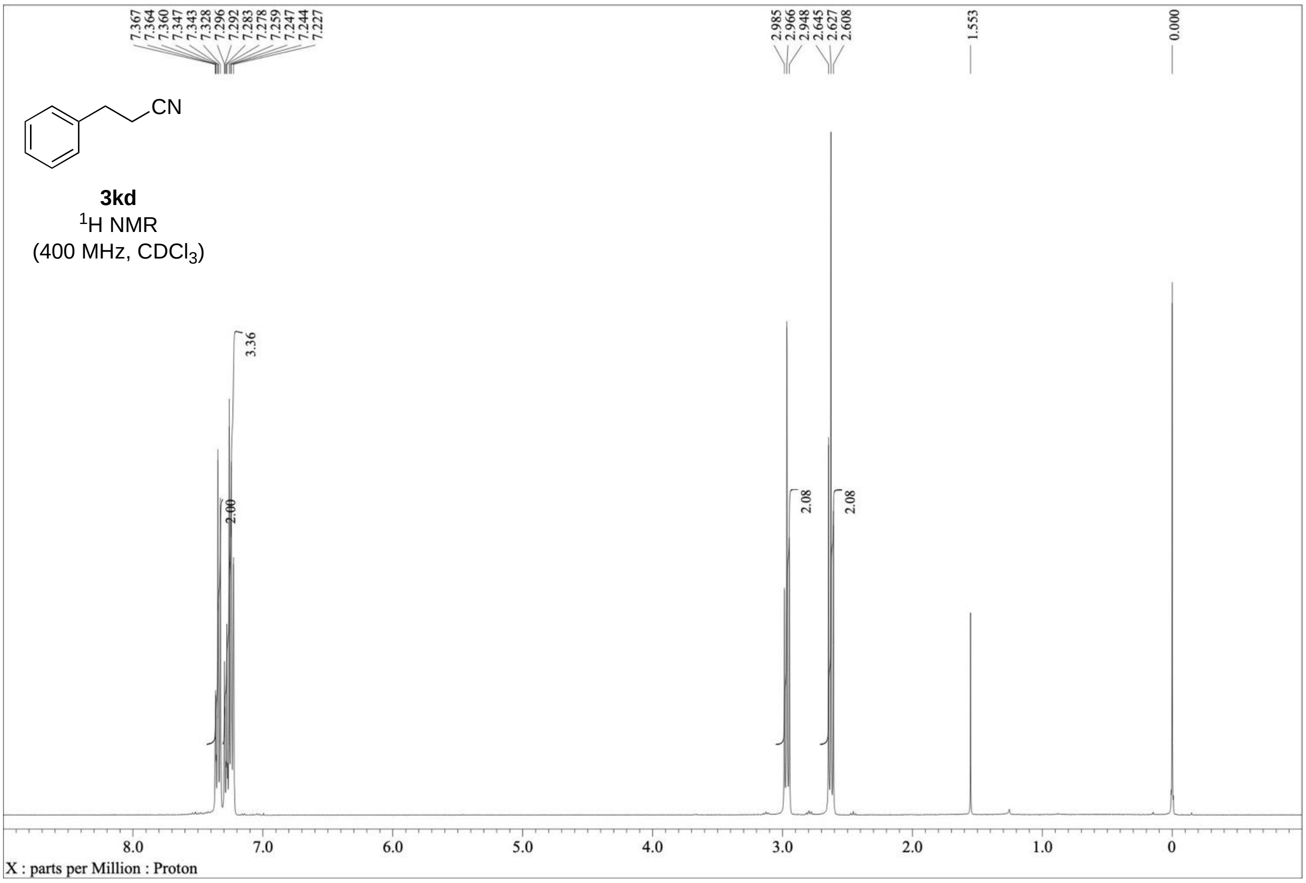
CN
3kd
1H NMR
(400 MHz, CDCl3)
7.367
7.364
7.360
7.347
7.343
7.328
7.296
7.292
7.283
7.278
7.259
7.247
7.244
7.227
2.985
2.966
2.948
2.645
2.627
2.608
1.553
0.000
3.36
2.00
2.08
2.08
8.0
7.0
6.0
5.0
4.0
3.0
2.0
1.0
0
X : parts per Million : Proton

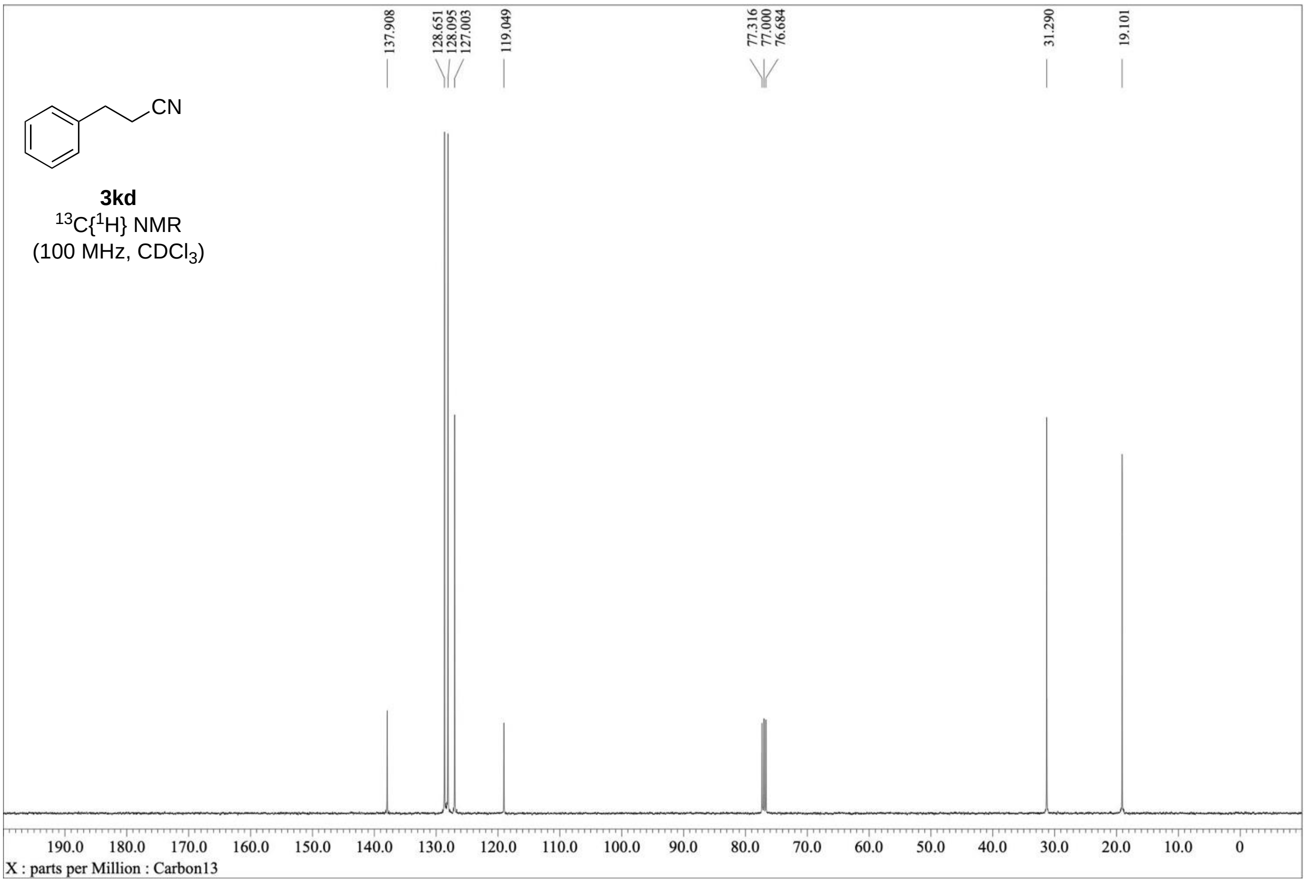
CN
3kd
13C{1H} NMR
(100 MHz, CDCl3)
137.908
128.651
128.095
127.003
119.049
77.316
77.000
76.684
31.290
19.101
190.0
180.0
170.0
160.0
150.0
140.0
130.0
120.0
110.0
100.0
90.0
80.0
70.0
60.0
50.0
40.0
30.0
20.0
10.0
0
X : parts per Million : Carbon13

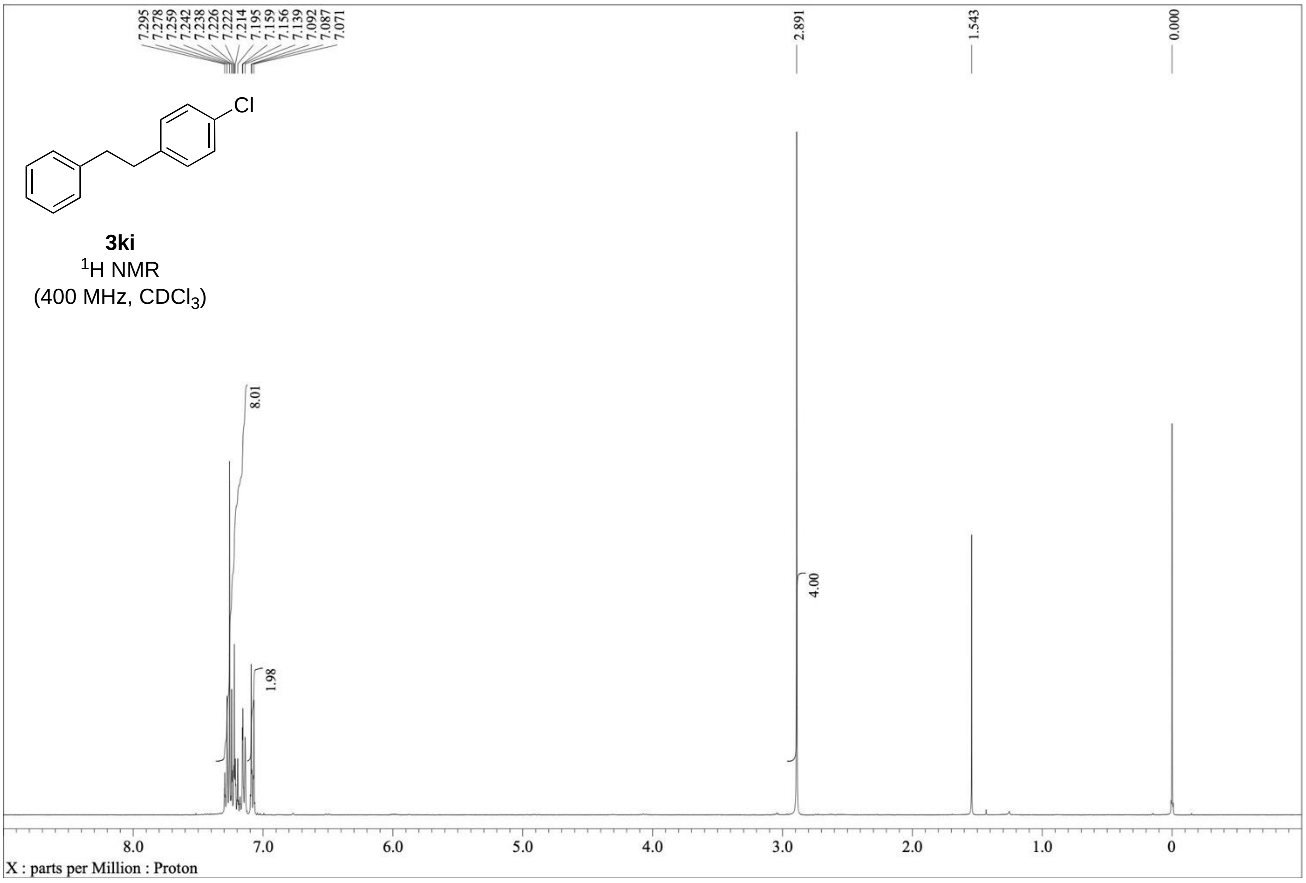
7.295
7.278
7.259
7.242
7.238
7.226
7.222
7.214
7.195
7.159
7.156
7.139
7.092
7.087
7.071
2.891
1.543
0.000
Cl
3ki
1H NMR
(400 MHz, CDCl3)
8.01
1.98
4.00
8.0
7.0
6.0
5.0
4.0
3.0
2.0
1.0
0
X : parts per Million : Proton

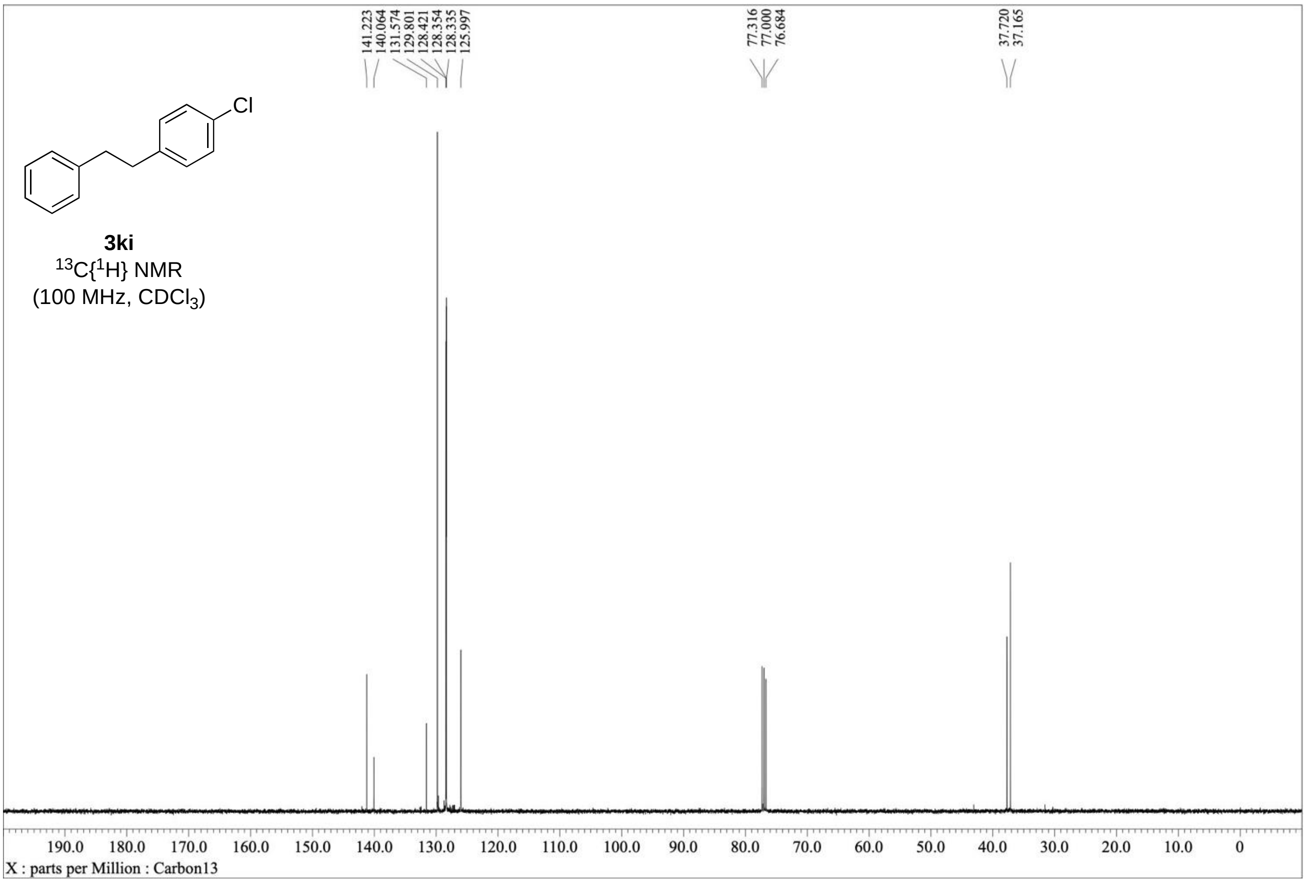
141.223
140.064
131.574
129.801
128.421
128.354
128.335
125.997
77.316
77.000
76.684
37.720
37.165
Cl
3ki
13C{1H} NMR
(100 MHz, CDCl3)
190.0
180.0
170.0
160.0
150.0
140.0
130.0
120.0
110.0
100.0
90.0
80.0
70.0
60.0
50.0
40.0
30.0
20.0
10.0
0
X : parts per Million : Carbon13

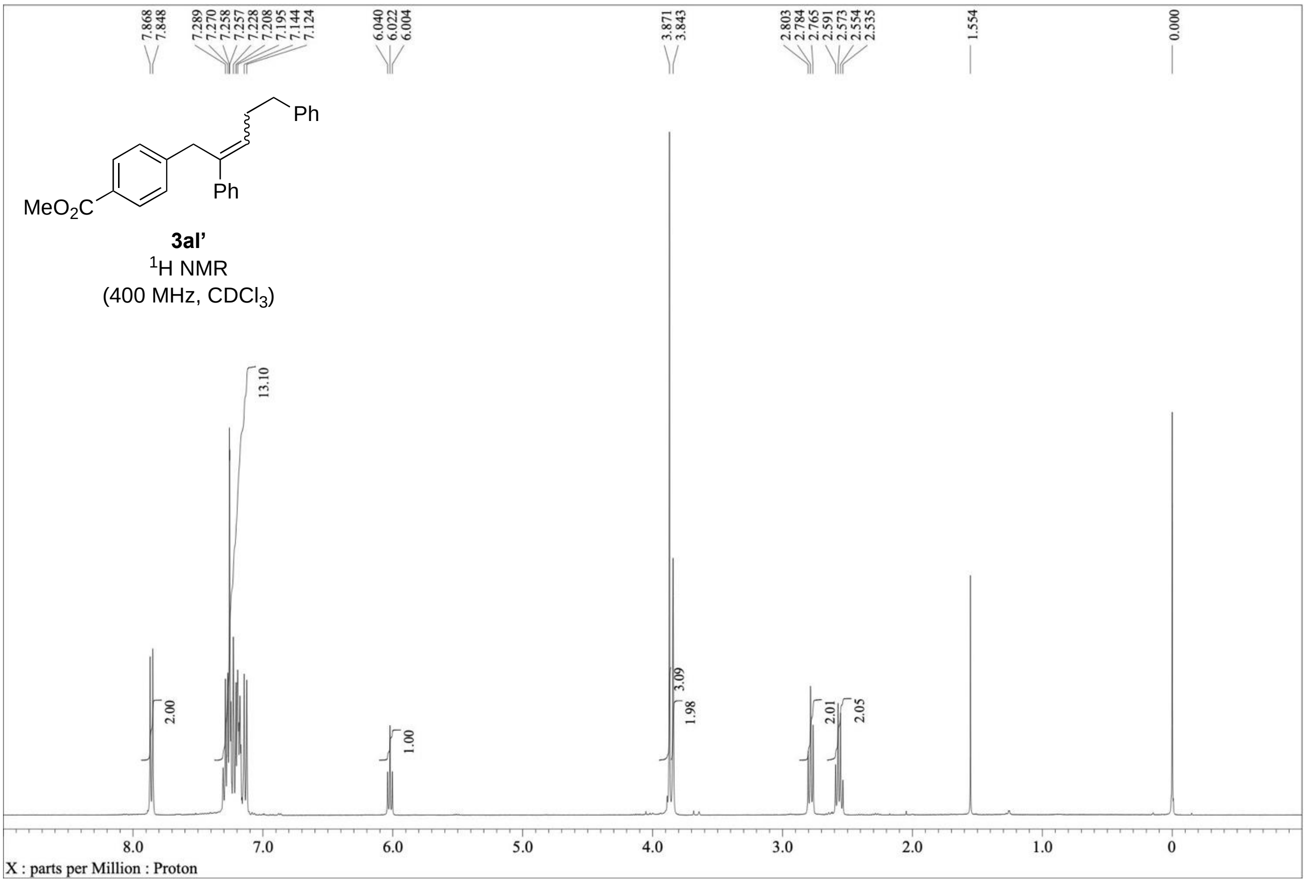
7.868
7.848
7.289
7.270
7.258
7.257
7.228
7.208
7.195
7.144
7.124
6.040
6.022
6.004
3.871
3.843
2.803
2.784
2.765
2.591
2.573
2.554
2.535
1.554
0.000
Ph
MeO2C
Ph
3al'
1H NMR
(400 MHz, CDCl3)
13.10
2.00
1.00
3.09
1.98
2.01
2.05
8.0
7.0
6.0
5.0
4.0
3.0
2.0
1.0
0
X : parts per Million : Proton

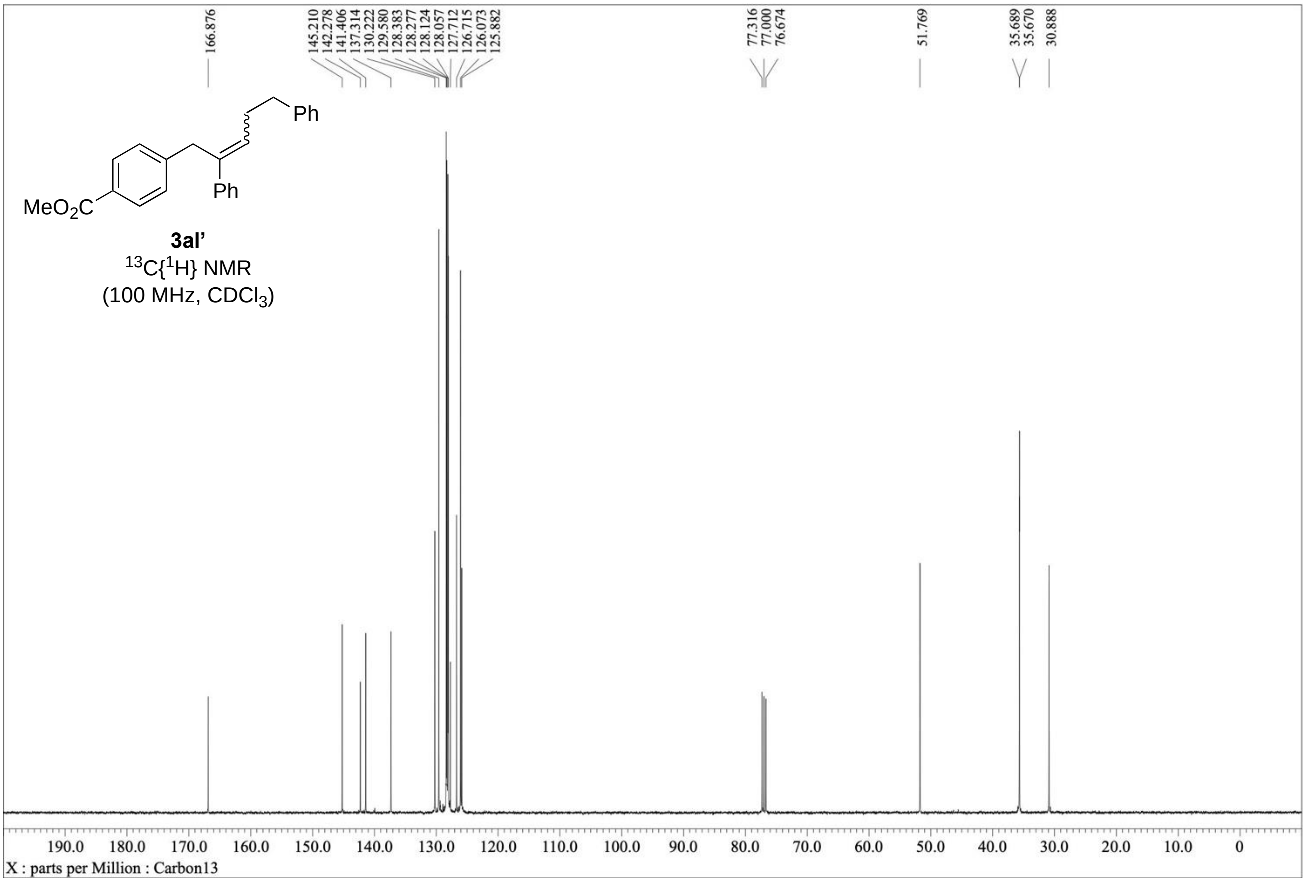
166.876
145.210
142.278
141.406
137.314
130.222
129.580
128.383
128.277
128.124
128.057
127.712
126.715
126.073
125.882
77.316
77.000
76.674
51.769
35.689
35.670
30.888
Ph
MeO2C
Ph
3al'
13C{1H} NMR
(100 MHz, CDCl3)
190.0
180.0
170.0
160.0
150.0
140.0
130.0
120.0
110.0
100.0
90.0
80.0
70.0
60.0
50.0
40.0
30.0
20.0
10.0
0
X : parts per Million : Carbon13

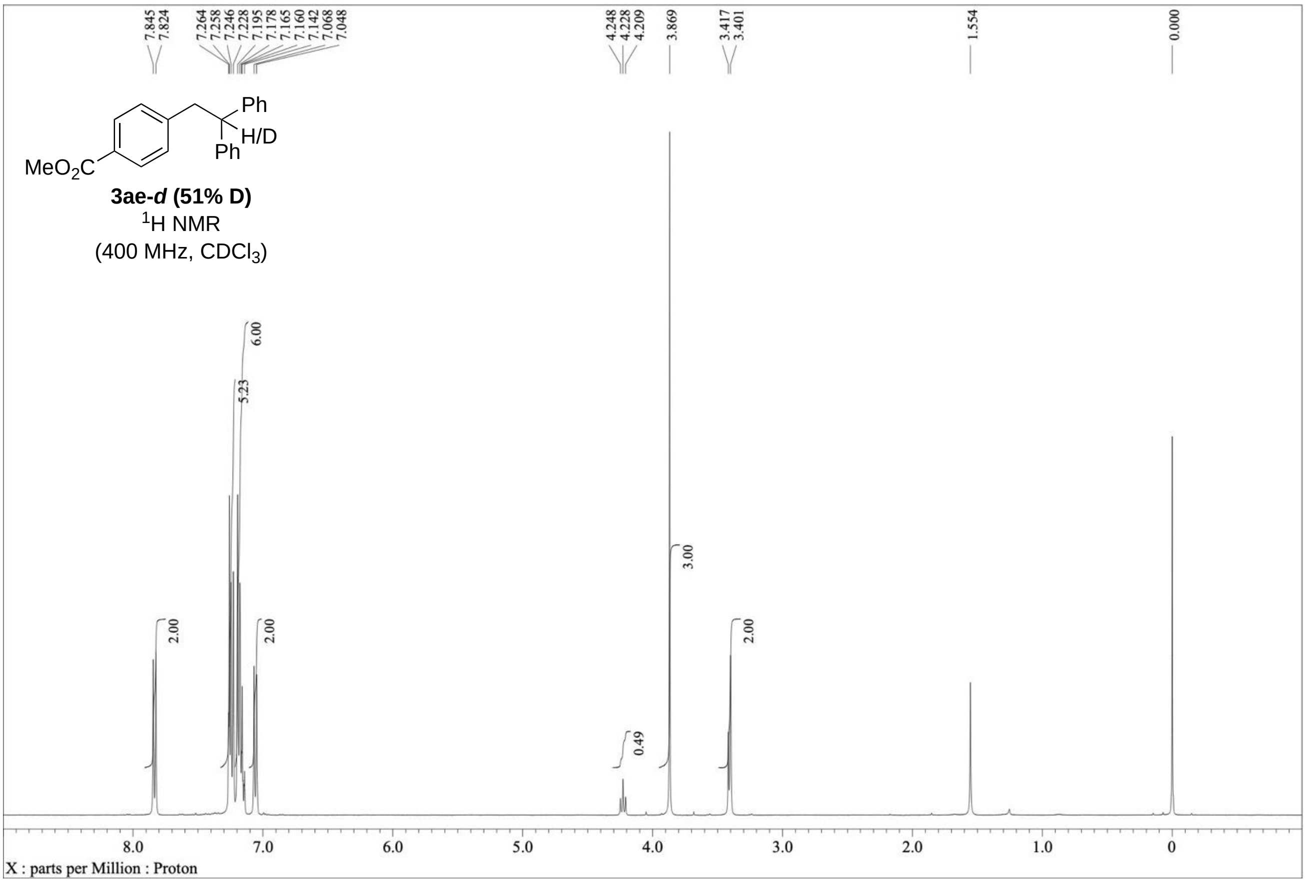
7.845
7.824
7.264
7.258
7.246
7.228
7.195
7.178
7.165
7.160
7.142
7.068
7.048
4.248
4.228
4.209
3.869
3.417
3.401
1.554
0.000
Ph
H/D
Ph
MeO2C
3ae-d (51% D)
1H NMR
(400 MHz, CDCl3)
6.00
5.23
3.00
2.00
2.00
2.00
0.49
8.0
7.0
6.0
5.0
4.0
3.0
2.0
1.0
0
X : parts per Million : Proton

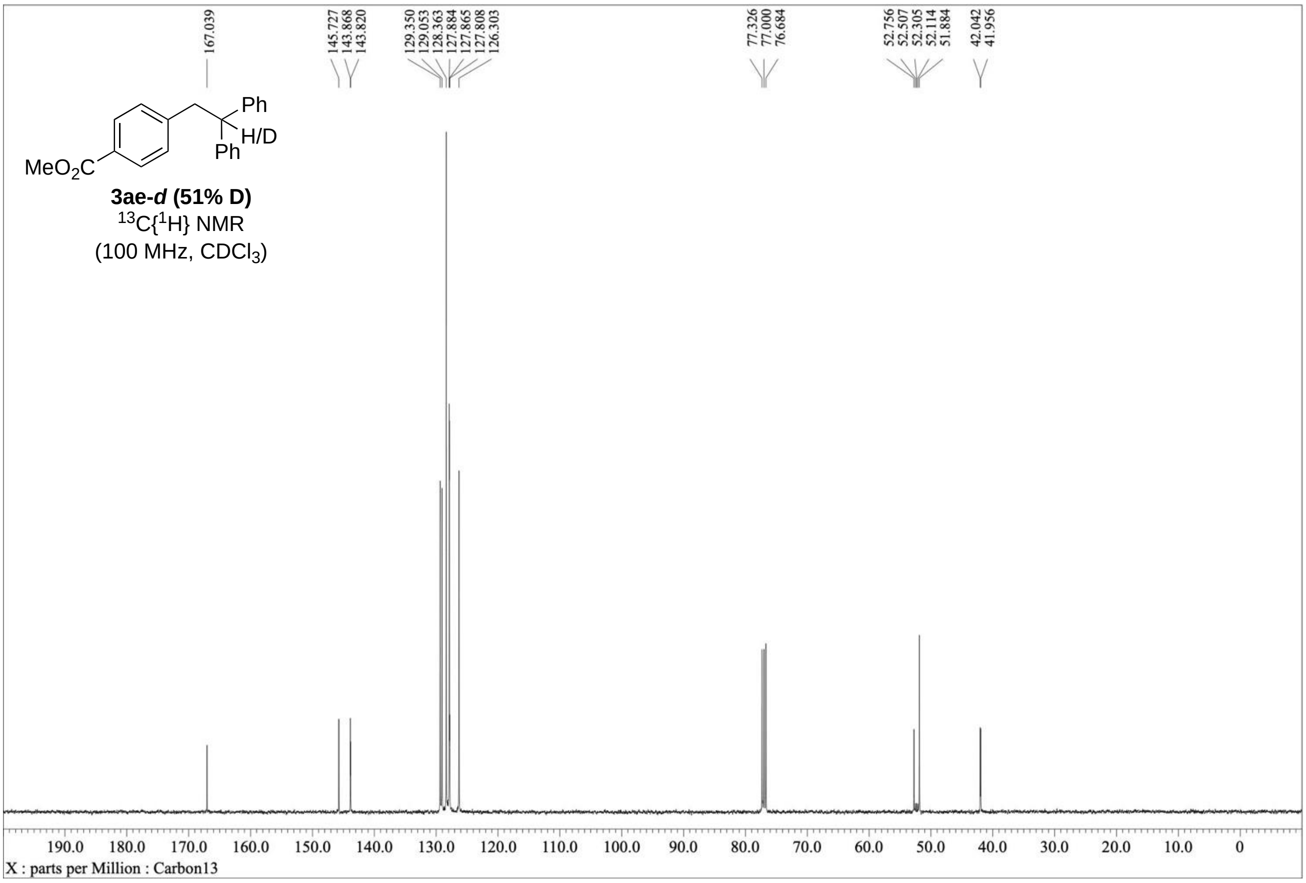
167.039
145.727
143.868
143.820
129.350
129.053
128.363
127.884
127.865
127.808
126.303
77.326
77.000
76.684
52.756
52.507
52.305
52.114
51.884
42.042
41.956
Ph
H/D
Ph
MeO2C
3ae-d (51% D)
13C{1H} NMR
(100 MHz, CDCl3)
190.0
180.0
170.0
160.0
150.0
140.0
130.0
120.0
110.0
100.0
90.0
80.0
70.0
60.0
50.0
40.0
30.0
20.0
10.0
0
X : parts per Million : Carbon13
